# A critical assessment of a protected area conflict analysis based on secondary data in the age of datafication

**DOI:** 10.1038/s41598-023-35067-x

**Published:** 2023-05-17

**Authors:** Marcin Rechciński, Joanna Tusznio, Arash Akhshik, Małgorzata Grodzińska-Jurczak

**Affiliations:** 1 Faculty of Geography and Geology, Institute of Geography and Spatial Management, Jagiellonian University in Krakow, Gronostajowa 7, 30-387 Krakow, Poland; 2 Faculty of Biology, Institute of Environmental Sciences, Jagiellonian University in Krakow, Gronostajowa 7, 30-387 Krakow, Poland; 3 School of Business and Economics, Linnaeus University, Kalmar, Sweden

**Keywords:** Environmental social sciences, Conservation biology

## Abstract

Recently, a global trend towards a broader use of secondary data in social sciences has been reinforced by the COVID-19 pandemic. This evoked doubts about the validity of the results unless restrictive assessment procedures are implemented. To address this need in the field of protected area (PA) conflict analysis, we propose a three-fold approach (theory-, method-, and cross-scale simulation-driven) to assess the usefulness of the utilized state register dataset and the indicator analysis methodology for the multi-level recognition of PA conflict determinants. With the ultimate aim to inform case study selection, we processed 187 relevant indicators from the official Statistics Poland register for a Lesser Poland region. We distinguished five types of PA conflict determinants in Lesser Poland (‘urbanity’, ‘agriculture’, ‘tourism’, ‘small-scale entrepreneurship’, and ‘sprawl’) and respective groups of 15 clusters comprising local-level units. For one cluster, we juxtaposed the obtained results with secondary data from another source (Internet content) and for a specific PA (Tatra National Park). Although the reported conflict issues corresponded to the indicator-derived descriptors of the cluster, in the theory-driven phase of the assessment, the state register failed to address the key prerequisites of PA conflicts. We have demonstrated that, in crisis conditions such as COVID-19, the proposed method can serve as a proxy for a multi-level recognition of PA conflict potentials, provided that it synthesises the results of different methodological approaches, followed by in-person interviews in the selected case studies.

## Introduction

The growing prominence of data-driven inquiries in social sciences has been widely debated for over a decade^1^. Despite the undeniable benefits of the increasing availability of secondary data^2–4^, the widespread datafication process^5^ has raised many ethical^6^ and epistemological^1^ concerns. More recently, the outbreak of the COVID-19 pandemic has further complicated the landscape for scholars across disciplines. All social science researchers, even those who had been working within the pre-Big Data paradigms^1^, have been compelled to adapt their well-established methodologies to new conditions. The availability of respondents decreased and data collection in many studies was moved online^7–9^; however, the adaptation strategies, regarding both study designs and sampling, were often applied independently by individual researchers^10^. Although such methodological innovations are perceived to be beneficial^11^ for qualitative enquiries, the reliability of quantitative social studies became threatened^12^. Navigating the complexities of data overload, pandemic-induced challenges, and misinformation in contemporary social science research requires a thorough understanding of multiple data sources, the use of multi-method approaches, and both data triangulation and subsequent validation of the whole process^13^, often starting from selection of available secondary data through different modes of its processing and analysis.

The challenges become more significant in research fields that require the adoption of a constructionist/constructivist approach to the subject of the study, such as protected area (PA) conflict analysis^14,15^. It has been reported that even the use of direct on-site surveys, consisting of closed-ended statements, can lead to bias in the responses collected on PA perceptions^16^. Furthermore, replacing in-person surveys with the online modes may systematically reduce response rates from certain groups of PA actors, such as elderly residents^17^. At the same time, a concurrent call for a broader use of open and in-depth approaches in conservation social science^16^ cannot be applied effectively to broad quantitative research requiring large samples. Thus, given the multi-level character of PA socio-ecological systems^18^, it is a case study selection stage which becomes the bedrock of any further analysis. Although the selection is often based on criteria derived from existing secondary data, it remains uncertain which specific types of secondary data can be considered suitable for such purposes.

In general terms, the use of secondary data has been accepted in theories of PA conflicts (or, more broadly, conservation conflicts), especially when they are enriched with empirical social data^14,19,20^. However, there are some concerns stemming from the variety of conceptual frameworks. First, most secondary data indicators are assumed to present ‘actual’ measurements of certain conflict properties, with a positivist claim for their objectivity^21,22^. As such, they should never be confused with the stakeholders’ constructions of reality, the clash of which is the actual reason for the conflict emergence^14,19,23^. Apparently, the problem could be addressed by incorporating user-generated big data that are inherently subjective, such as social media content^24,25^. However, some characteristics of these datasets, such as their highly unsystematic nature or loosely defined populations and samples^4^, have profound epistemological implications for their analysts^1,26^. Second, even if a researcher interprets the secondary data indicators solely as a proxy for ‘conflict potential’^20^ recognition, caution must be exercised regarding spatial mismatches of measures representing different groups of conflict determinants^19^. Indeed, some conflict properties, which may be independently represented by different types of secondary data, inherently adhere to different scales^14,18,27^. For example, institutional determinants, which can be addressed by analysing public consultation reports^28^, belong to the managerial, jurisdictional or institutional scales; economic determinants (e.g., inspecting financial operations data^29^) to the network scale, while environmental determinants (e.g., investigating remote sensing data^30^) to the spatial scale. PA management itself relates to different geographical scales, which may overlap on some levels but diverge on others. Specifically, the institutional framework of a PA system (here referred to as ‘a spatio-institutional scale’) may not fully adhere to an administrative division of a country (here: ‘a spatio-administrative scale’). Although it is possible to obtain relevant indicators reduced to one of the scales, most often the researcher faces a trade-off between a multi-determinant spectrum of the available indicators and scale-related consistency of the whole dataset. Arguably, the trade-off is best compromised by using multi-level official statistics^31^, which have already been applied to the field of PA conflicts^32,33^. However, a multi-perspective assessment of neither these datasets nor the whole process of their use in multi-level data triangulation has been carried out in the current state of the art.

To fill this gap, we conducted a regional study, based on secondary data from the official Statistics Poland register, aiming to (1) identify the main types of PA conflict determinants in the selected region, recognise their local-level clusters and their spatial structure that can work as a basis for informed site selection, and (2) assess the usefulness of the analyses for recognising PA conflict determinants and potentials at every stage of the research process (data collection, preparation, analysis, and triangulation). Three perspectives were used for the assessment:

1. theory-driven, to discuss the data and results from the perspective of PA conflict theory;
2. method-driven, to compare various methods of data analysis and to provide heuristics based on the comparison;
3. cross-scale simulation-driven, to verify whether the obtained premises for a case study selection (cross-level analysis of a spatio-administrative scale) correspond with evidence for a specific PA conflict (a level of a single tenure unit, spatio-institutional scale) from another source of secondary data.

The whole process was guided by the general aim of providing a proxy for scholars to study PA conflicts in a multi-level, mixed-mode manner in times of reduced access to PA stakeholders.

## Methods

### Study area

To ensure the best possible balance between the availability of indicators and their spatial consistency, we limited our study to a single country with a unified hierarchy of administrative units. We chose Poland to build on the existing systematic literature review of PA conflicts in this country (for a summary, see Supplementary Information S2 online)^34^. Following the referential theoretical framework for PA conflict analysis^14^, we narrowed the spatial scope of the study to a regional level. Of the 17 NUTS-2 units of Poland^35^, we selected a Lesser Poland voivodeship due to its highest diversity in terms of historical, cultural, physico-geographical, and nature conservation conditions (for a sketch map and broader rationale for the selection, see Supplementary Information 1A online).

### Data collection

**Figure 1:**
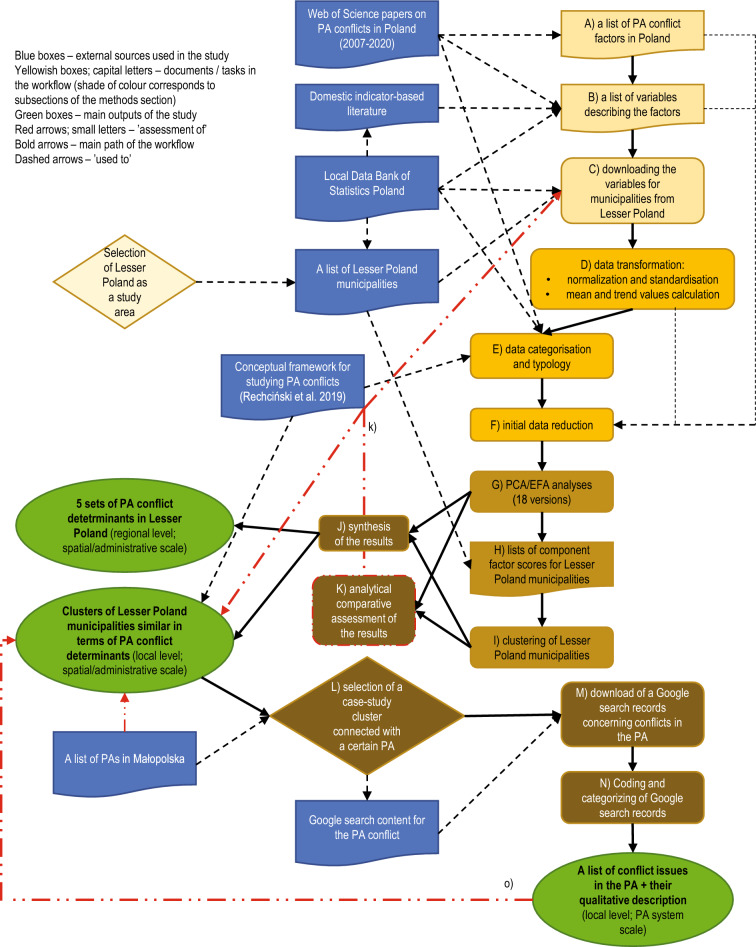
Workflow of the study. The steps of the procedure were marked with successive letters of the Latin alphabet. References to the steps are provided in the text.

The list of conflict factors in Poland that could be presented on an interval measurement scale was prepared based on a systematic review of all relevant manuscripts stored in the Web of Science database published between 2007 and 2020 (Fig. 1, step A; for a complete list of articles, see Supplementary Table S2.1 online)^34^. In the case of a compound character of a reported factor (e.g., socio-economic development), we searched for variables used in the domestic literature to describe such a factor (Fig. 1, step B; a complete list of the factors, selected variables, and sources of their use can be found in Supplementary Table S3.1 online; please note the difference between conflict determinants and conflict factors^14^).

We used secondary data indicators from the Local Data Bank of Statistics Poland^36^. This is the largest Polish database containing more than 40,000 economic, social or environmental data and indicators that describe administrative units in Poland referred to statistical units according to the NUTS nomenclature^37^. The data (variables) are grouped into 33 general categories (e.g. K3 Population, K18 Tourism, K27 Public finance) and subdivided into groups (e.g., G534 Births and deaths. G8 Internal and foreign migrations) and subgroups (e.g., P2167 Live births by singular age of mother, P2346 Gross fertility and reproduction rate)^36^. Not all categories of data are available for every level of an administrative scale, but the dataset has still been widely used as a basis to compare local-level units^38–40^. For each of the specified variables, we checked their availability in the register at the local level (i.e., LAUs, according to Eurostat nomenclature^41^). If existent, we downloaded relevant records for all 182 municipalities in the Lesser Poland voivodeship for a set timeframe (2007–2020). Ultimately, we managed to download data for 187 variables from 80 different LDB subgroups (Fig. 1, step C).

### Data preparation

The data preparation phase was guided by the intention of using principal component/exploratory factor analysis (PCA/EFA) for the initial reduction of the dataset and the exploration of its latent constructs^42,43^. Since neither PA conflicts nor their determinants are time-invariant, we aimed to obtain indicators representing both the substantial and the processual dimensions of a conflict property^14^. We found this possible for most of the collected variables, with the available time series data. Substance indicators calculated for each municipality and variable were arithmetic mean values of all available annual data. In the case of processual indicators, we intended to determine whether a potential conflict property intensified or diminished in a certain locality and what was the rate of the process. Therefore, we used the slope values of the variable trendlines, calculated as follows:

$${I}_{p}=\frac{\sum (x-\overline{x})(y-\overline{y})}{\sum {(x-\overline{x})}^{2}},$$

where x was an ordinal-scale value representing the year of measurement (where ‘1’ being the earliest time section, for which data were available) and y was a municipality-specific value of a variable measured for a particular year. The decision to generate indicators for both conflict dimensions increased the number of variables to 334 (for a full list, see Supplementary Table S3.3 online).

After normalisation or standardisation of the values (Fig. 1, step D), we performed both classification and typology of the variables into four groups of PA conflict determinants: socio-cultural, institutional, economic, and environmental^14^. For the data classification, we looked for the LDB categories into which particular variables were grouped. For data typology, we looked back at the rationale for using certain variables in the reviewed articles on PA conflicts in Poland. As the variables always stemmed from specific conflict factors, we used these factors to make additional assignments, retaining the one already made during the classification process (Fig. 1, step E). Consequently, some variables achieved up to three different conflict determinant descriptors, but there were others still left with only one descriptor (see Supplementary Table S3.1 online).

As the number of variables was too large to meet PCA/EFA assumptions^44^, we constructed correlation tables of all variables (first—normalized, then—standardised) and inspected pairs of variables with correlation coefficients greater than |0,900|. We deleted one of the two variables if (1) in the source papers both variables were referred to the same factor(s) (we excluded the one used less often in the papers), or (2) the substance and processual indicators of the same variable were correlated (we excluded the processual one) (Fig. 1, step F; for a complete list of variables, see Supplementary Table S3.2).

### Data analysis

To control the impact of the analytical method or input data transformation on the final result, we compared the results of 18 separate analyses (Fig. 1, step G). This included combinations of the following sets of assumptions (later referred to by the acronyms in the quotation marks).

1. Principal Component Analysis (PCA) and Exploratory Factor Analysis (EFA) with:
2. Mean and trend values <*μ&a*> vs. only mean values <*μ only*> as input data.
  - (for <*μ&a*> only) normalised values <*01*> vs. standardised values <*z-sc.*> as the input.
3. Predefined groupings of variables based on a theoretical model of conflict determinants <*pre-def.*> vs. no such groupings <*all var.*> 
  - (for <*pre-def*.> only) 1-step vs. 2-step analyses

The use of PCAs and EFAs is quite common in the field of conservation conflicts, with PCAs being used more often to reduce the number of indicators analysed^45,46^ and EFAs to recognise latent constructs behind scale-based statements of interviewed stakeholders^47,48^. We applied both as intended to achieve both goals and the mathematical outputs of the two procedures are not always similar^43^.

All analyses were performed using IBM^©^ SPSS^©^ Statistics version 27. For the <*1-step; pre-def.*> procedures, we performed four separate PCAs, one for each group of determinants. The only difference between <*pre-def. PCAs*> and <*pre-def. EFAs*> was a selection of variables for the analyses. For PCAs, we used classified variables (each could have been used for one analysis only), whereas for EFAs, the same variables could have been repeated across the analyses. This intentionally violated the assumption of the orthogonal character of principal components (PCs)^49^ and allowed us to interpret them as factors (Fs). As the <*1-step*> procedures generated a multiple number of PCs/Fs compared to the other analyses, we also performed <*2-step*> procedures, where the PCs/Fs from the first step were used as input data for other PCAs. When necessary, we performed dimension reduction of the variables until a positive definite correlation matrix and a target KMO ≥ 0.5 were achieved (for more details of the procedures applied, see Supplementary Information S4 online)^48,50^.

To generate a geographical image of PA conflict determinants in Lesser Poland, we performed a set of clustering procedures, using component/factor scores from analyses with KMO values exceeding 0.5 (Fig. 1., step H). We proceeded with a hierarchical cluster analysis using Ward’s clustering method^51^. Each analysis was preceded by a test one, which was performed to determine the desired number of clusters. The target numbers were specified based on the analysis of the dendrograms, and each time, we selected a clustering level at which Kraków, the capital of the region, was left as a separate cluster of one element (Fig. 1., step H; for more arguments for the decision, see Supplementary Information 1A, and for the other approach considered, see Supplementary Information S4 online).

### Format of the results presentation

We applied a synthetic approach for the presentation of results for the first aim of the study and an analytical and case study approach for the results supporting the second aim.

To present a structure of PA conflict determinants in Lesser Poland, we compared all the obtained <*all var.*> and <*2-step pre-def.*> PCs/Fs (Fig. 1, step J). Based on the detected similarities, we distinguished five types of PA conflict determinant sets. We then interpreted and described them by looking at the raw variables or <*1-step pre-def.*> PCs/Fs that most strongly loaded <*all var.*> or <*2-step pre-def.*> PCs/Fs, respectively.

We then collected all the ten hierarchical clustering results, which resulted in spatially informative results. As the number of clusters differed across analyses, we classified them into five universal groups. Subsequently, for each municipality, we calculated the number of assignments for each group of clusters. A municipality was ultimately assigned to a cluster group that had been assigned most often across different versions of the analysis. In the last step, some assignments were refined based on the structure of cluster groups derived from versions of the analyses that yielded a higher number of clusters (Fig. 1, step J).

We assessed the usefulness of the results obtained using a comprehensive conceptual framework to study PA conflicts^14^ as a benchmark. Specifically, we inspected whether the dataset and the output were complete from the perspective of theoretical requirements (Fig. 1, step k). At the analytical stage, we compared all variants in terms of their numeric characteristics (no. of excluded variables; no. of required iterations, KMO values, no. of the determined clusters) and interpretive power. All observations have been presented in the form of lists of advantages and disadvantages of certain methods (Fig. 1, step K).

Finally, for the case study analysis, we selected a cluster of municipalities of similar PA conflict determinants, which we hypothesised to be most affected by a specific PA (Fig. 1, step L). Deliberately, we capitalised on the fact that municipalities and PAs pertain to different spatial scales (the former—to a spatio-administrative scale, while the latter—to an institutional scale^18^; there are examples of municipalities containing a number of PAs, while there are also PAs located in more than one municipality). Responding to the recent trend of using text mining in the field of environmental conflicts^52,53^ and validating data through their triangulation^13^, we used a Google search^©^ engine to search for all Polish websites and PDF/DOC files that included search terms ‘*(name of the PA)* AND conflict* OR dispute’. We downloaded all search results using Octoparse^©^ software (Fig. 1, step M) and coded them in a MAXQDA 2020^©^, using a single search record as a measurement unit. We applied an open coding approach^54^, trying to recognise and name the addressed conflict issues^14^ and then categorised the codes into a hierarchical structure (Fig. 1, step N). Finally, we qualitatively assessed whether an indicator-based description of the relevant cluster (cross-level analysis, spatio-administrative scale) was reflected in the second source of secondary data (Fig. 1, step o), which described a single tenure unit on a spatio-institutional scale. We showcased some of the reported conflicts using the analysed Internet content and indicators that adhere to the latter level and scale.

## Results and discussion

### Determinants of PA conflicts in Lesser Poland based on state register secondary data

The final typology of PA conflict determinants in Lesser Poland was mainly driven by the structure of economic determinants (for a comprehensive presentation of all results, see Supplementary Information S5). This can be partially explained by the predominance of economic variables in the initial dataset. However, economic PCs/Fs were also the strongest in <*pre-def.*> procedures that were performed to counterbalance such disproportions. The 1-step procedures revealed that the top five economic PCs/Fs had counterparts in the social and, to some extent, environmental group of determinants. This was not the case for the institutional group of determinants, which offered only one PC/F that contributed to the final set of cross-determinant PCs/Fs (Table 1).

**Table 1:** Typology of PA conflict determinants in Lesser Poland (see Supplementary Information 1A online for a region-specific explanation).

Types of PA conflict determinants in lesser Poland	Selected <*1-step pre-def*.> principal components/factors and their description			
	Economic	Social	Institutional	Environmental
1. “Urbanity”	Urban economy (well-developed infrastructure, specialised services, high but dropping share of own revenue)	Urban population and society (high population density, high employment, good access to social services)	*No clear association*	Urban environment (urbanised/industrial lands, urban greenery, water pollution)
2. “Agriculture”	Agricultural economy (incl. well-developed but dropping administrative sector)	Ageing society	Large properties	Agricultural lands, high eutrophication
3. “Tourism”	Tourism industry (tourist infrastructure, large number of EU grant applications)	Tourist density	*No clear association*	National parks and forests (private forest removals)
4. “Small-scale entrepreneurship”	*Negative loads* Public sector (low share of subventions and targeted grants, low total revenue per capita)	Working-class society	*Negative loads* large properties	*No clear association*
5. “Sprawl”	Residential investments (incl. specialised services, high entrepreneurship)	Inflow of inhabitants, high standards of living	*No clear association*	*No clear association*

**Figure 2:**
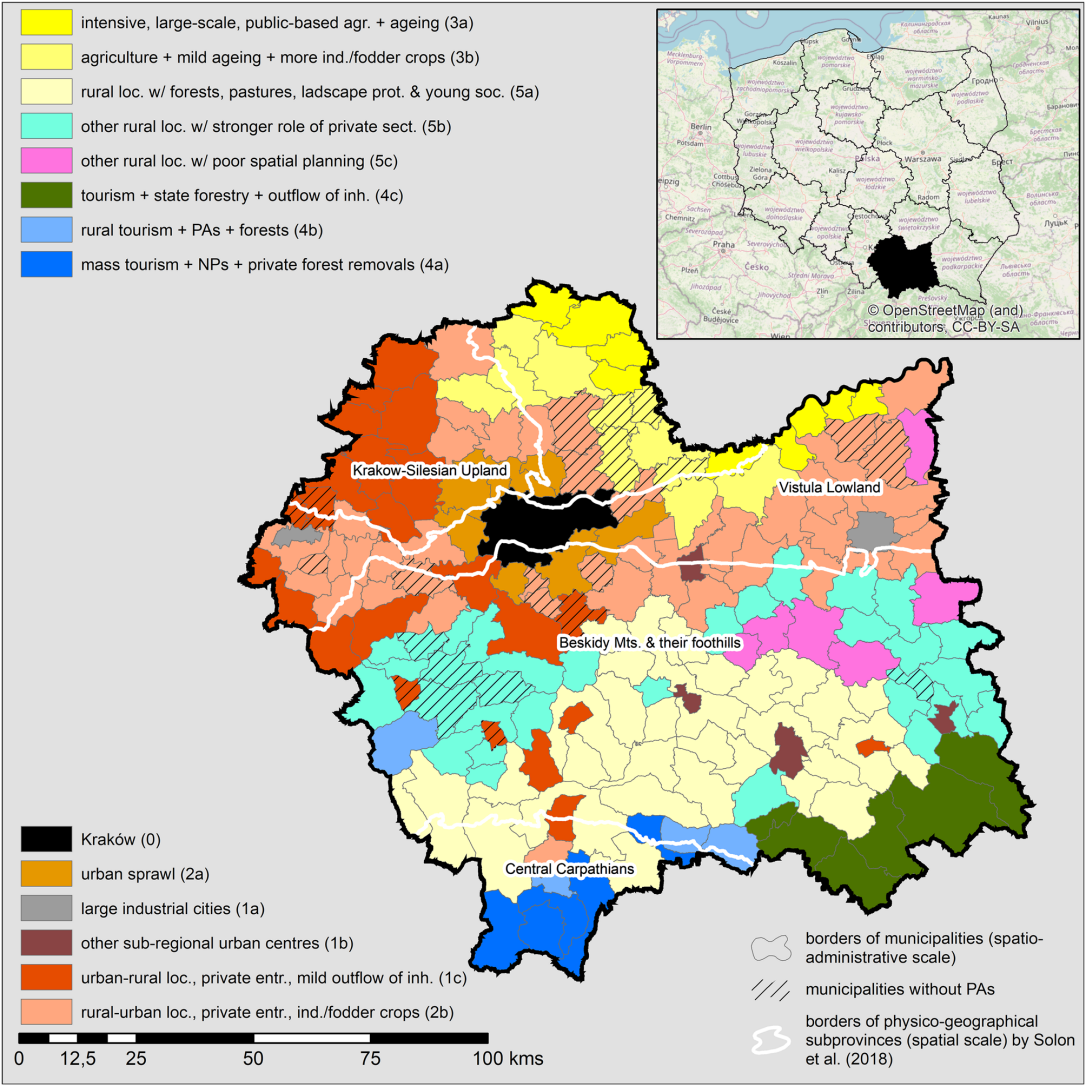
Spatial structure of clusters of municipalities in Lesser Poland similar in terms of their PA conflict determinants. The cluster numbers in brackets are referred to in Table 2.

The five types of PA conflict determinants in Lesser Poland can be broadly referred to as five universal groups of PA conflict clusters: urban, agricultural, tourist, other rural localities, and rural localities under transition. However, the results of <*1-step; pre-def.*> procedures with a larger number of PCs/Fs led to a more diverse and specific depiction of the 14 clusters (Fig. 2 and Table 2). Additionally, the relationship between the types of PA determinants and the resulting clusters was not straightforward, since the clustered municipalities were always characterised by a combination of component/factor scores. In other words, no municipality could be considered an ideal example of a type of PA conflict determinants. For instance, Kraków, the capital of the Lesser Poland voivodeship, the second largest city in the entire country, and the only cluster of one element on the map (Table 2, a cluster ‘0’), differed from the other clusters mainly in terms of the scores of the ‘urban’ component/factor. Simultaneously, it should be noted that^55,56^: (1) approximately 7000 ha of arable land exists within the administrative boundaries of the city (large ‘agricultural’ scores despite remaining outside the ‘3x’ clusters), (2) prior to the COVID-19 pandemic, Kraków welcomed up to 14 million visitors annually (high ‘tourist’ scores, yet outside of the ‘4x’ clusters), (3) over 35% of the city budget consists of public grants and subventions (negative ‘small-scale entrepreneurship’ scores), and (4) in the past two years, almost 20,000 new residential investments have commenced (high ‘sprawl’ scores, yet outside of the ‘2x’ clusters). Finally, the extended set of clusters exposed new groups of determinants and, in some cases, the processual dimension of the determinants. This mainly concerns institutional properties describing a level of spatial planning in a municipality (that is, a number of land development decisions compared to a share of a municipality area covered by valid local spatial development plans; a cluster ‘5c’) and a few environmental factors (increasing water pollution that describes a cluster ‘1a’ or types of cultivated crops/agricultural lands that helped to specify rural clusters).

**Table 2:** List of clusters of municipalities in Lesser Poland, similar in terms of their PA conflict determinants.

No	Merit description	Geographical description
0	**Kraków**	
1	**Urban clusters other than Krakow**	
1a	Large cities with growing industry	Oświęcim and Tarnów only
1b	Subregional urban centres with tourism and residential sprawl less important than in Kraków	Bochnia, Limanowa, Nowy Sącz, Gorlice
1c	Urban–rural localities with strong private entrepreneurship and a mild outflow of inhabitants	North-West part of the region and second-rate subregional urban centres
2	**Rural localities in transition**	
2a	Most intense urban sprawl	A belt of closest municipalities around Kraków
2b	Other rural–urban localities with strong private entrepreneurship and agriculture directed to industrial/fodder crops	Second-rate belt around Kraków and three linear zones toward north, west and east from Kraków
3	**Agricultural clusters**	
3a	The most intense agriculture in large-scale properties and with a strong role of the public sector with an ageing society	Most north-eastern part of the region
3b	Other agricultural localities with a less advanced ageing process and a higher share of industrial/fodder crops	The rest of the north-eastern part
4	**Tourist clusters**	
4a	Mass tourism with national parks and high rate of private forests removals	Tatra NP municipalities and Czorsztyn by the Dunajec River Gorge in Pieniny NP
4b	Rural tourism with forests and protected areas	Zawoja in the Babia Góra NP and remaining Tatra NP and Pieniny NP municipalities
4c	Tourism, forestry, and outflow of inhabitants	Spa tourism municipalities in the south-eastern part of the region
5	**Other rural clusters**	
5a	Localities with forests, pastures, landscape protection and young society	Mountainous municipalities in the central-south
5b	Localities with higher role of private sector	Mountainous municipalities in the central-west and the central-east
5c	Localities with poor spatial planning	Scattered municipalities, mostly in Tarnów subregion

### Usefulness assessment of the analyses and dataset

#### Assessment based on a theory of PA conflicts

Despite the rich character of the results obtained, their application to PA conflict studies encompasses certain limitations that stem from the theory of PA conflict. This is mainly because secondary-data indicators from official data banks do not provide insight into key prerequisites of conflicts^14,23,57^, which are conflicting interests of parties and the mutual perception of these interests. Another prerequisite, the involvement of at least two conflicting parties, is also challenging to be determined based solely on secondary data. However, given an understanding of the general context of PA conflicts in a certain country, it is feasible to identify potential stakeholders that clash with an ‘environmental coalition’^58,59^ for specific clusters. In our study, these could include real estate developers and local authorities^60^ (Table 2., clusters ‘2x’), large-scale agricultural owners^61^ (clusters ‘3x’), State Forest officials^62^ (clusters ‘4c’), small-scale property owners^63^ (clusters ‘5x’), ‘tourist entrepreneurs’ (clusters ‘4x’), or certain types of tourists and private forest owners (cluster ‘4a’—see Sect.  3.3.3. for cross-scale confirmation). Furthermore, the high absolute values of different component/factor scores for Kraków support the claim that the highest potential for complexity of clashing stakeholders and interests arises in large cities^64,65^.

**Figure 3:**
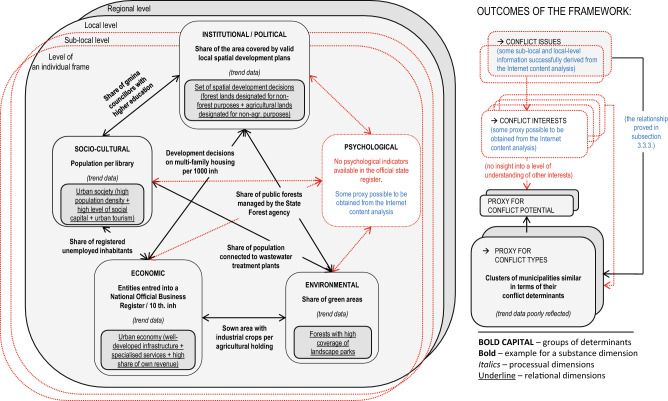
Examples of the indicators analysed and interpretation of results based on a conceptual framework for studying PA conflicts^19^. The shade of boxes indicates a level on a spatial scale (light grey—local level, dark grey—regional level). The red colour indicates elements of the framework that are not reflected in the indicator-driven part of the study. Gaps that can be potentially filled in with the use of secondary data content analysis are shown in blue fonts.

As the dataset does not fully cover the definition of PA conflict, it does not reflect many attributes of the conceptual framework for studying PA conflicts. This mainly concerns attributes that represent the constructionist/constructivist aspect of PA conflict inquiries^14^. Specifically, the dataset used in our study lacks measures of psychological and individual-level determinants of PA conflicts (Fig. 3), despite the growing recognition of these aspects in current conservation conflict studies^66,67.^ Furthermore, variables classified into social or institutional groups of determinants do not represent essential characteristics of PA conflicts, such as social norms^68^, measures of social trust^69^, models of decision-making, or power imbalances^70^. However, the ‘positivist’ properties of conflicts can still be valuable in interdisciplinary conflict analysis, provided they are interpreted solely as potential subjects for further stakeholder recognition^14^.

Our efforts to address the processual dimension of PA conflicts^14^ achieved moderate success, as the ultimate impact of trend values on the final results was not crucial (for more details, see Supplementary Information S5B online). At the same time, the results of all EFAs confirmed the vital role of interactions across different groups of determinants^14^, as the resulting factors were always loaded with variables from all groups. Finally, we addressed the need for data-driven PA conflict typology^14^. In our case, the results of both PCAs/EFAs and cluster analyses can serve as a proxy for such typology, however, we find the latter to be more informative, as clusters feature a more ‘realistic’ combination of conflict properties (e.g. not restricted by the assumption of orthogonality of PCs) and offer a cross-level perspective of the subject (Fig. 3).

Some sources of database incompleteness stem from our conservative approach to the selection of input data. As a trade-off, some important PA conflict properties that can be presented in the form of interval-scale secondary data were not included in the analysis (Table 3; for an extended version, see Supplementary Table S3.2 online).

**Table 3:** Selection of interval-scale PA conflict data missing in the analysis.

Data availability	Examples and evidence for their role in PA conflicts in Poland (for more references, see Supplementary Information S4 online)
Not in the Local Data Bank (partially achievable in other datasets)	Biological diversity^54^
	Tourism-related indicators (number of one-day visitors, structure of tourism and tourist infrastructure—for example, length of ski lifts)^55,57^
Available in LDB but only for higher levels of spatial/administrative scale	Share of Natura 2000 size in a municipality^10,58^
	Operational data of State Forests^53,54^
	Hunting data^57^
Available in LDP for a local level but only in short time sections	Most of agricultural data^56^
	Water management investments^66^
	Land use data^67^

As PA conflicts are intrinsically related to the existence of PAs^19^, we inspected the role of PA-related variables in types of PA conflict determinants and stemming clusters. The PAs proved to be the most important for a ‘tourist’ type and respective clusters (‘4a’—national parks; ‘4b’, ‘4c’—across various legal designations). Furthermore, the rural cluster ‘5a’ was partially connected to protected landscape areas (for more details on the legal designations of PAs in Poland, see Supplementary Table S1.1 online). However, the overall impact of these variables on the results was low, as evidenced by the map of clusters; 24 municipalities without a PA did not form a separate ‘non-PA’ cluster, but were assigned to four different clusters (Fig. 2). There are at least three possible explanations for this finding. First, the share of PAs of different types was poorly correlated with other determinants of PA conflicts described in the literature. In other words, although the coexistence of a PA and other conflict-inducing determinants fuels particular PA conflicts at the local level, the relationship might not be general at the regional level. Second, the perception of certain conflict properties may loosely correspond to their ‘positivist’ measures in the data bank, which can be verified with a constructionist/constructivist approach to PA conflict analysis. Finally, the absence of Natura 2000 data in the dataset (Table 3), considered as crucial conflict determinants by numerous authors^28,63,71^, may alter the overall result, although there is evidence that relationships between the presence of Natura 2000 and several socio-economic indicators in Poland are meaningful only when a processual dimension is well addressed^72^.

#### Comparison of the methods

Despite apparent similarities among all the approaches used, their specific results may differ^43^, which has been demonstrated in the PA conflict study (see Supplementary Information S5 online). After analysing all the differences, we identified various advantages and disadvantages of all the applied approaches (Table 4).

**Table 4:** An overview of the advantages and disadvantages of all the methods and input data approaches used in this study.

Advantages	Disadvantages
**Mean + trend data <μ&a> (vs. mean data only <μ only>)**	
More comprehensive and complete dataset that reflects two PA conflict dimensions (substance and processual) More feasible interpretation of resulting components or factors Faster discrimination of a big city in cluster analysis, better reflection of functional connectivity of the city with its impact zone	Usually, contains a large set of data that requires, often arbitrary, data reduction measures prior to their use for PCAs/EFAs Lower pairwise correlations between trend and mean data affect the KMO measure and suitability of a whole dataset for PCA/EFA Trend data, when presented as both positive and negative values, are vulnerable to a scaling method
**Standardisation <z-sc.> (vs. normalisation <01>) of data**	
The variability of data after the scaling procedure is more similar to the original one Geographic output is more clustered, and more useful for regionalisation procedures	Negative trend values affect linear correlations with mean data (and KMO values of a whole dataset) More arbitrary process of determination of PCs/ Fs (large difference in eigenvalues of first PCs/Fs and the rest ones). Difficulties in interpretation of less evident PCs/Fs. Smaller number of eventual PCs/Fs included for clustering Less explanatory character of the final results due to a lower share of total variance explained by the used PCs/Fs
**Predefined variables <pre-def.> vs. all variables <all var.> **	
In many cases, the only way to perform the analysis (all-variables approach required arbitrary deletion of numerous variables without much gain on overall KMO value) Allows for balancing the impact of all determinants on the final result (especially in case of large disproportion in number of variables across the group of determinants) Offers a deeper insight into the structure of results, Better connect a dataset with a theoretical framework	Requires a clear rationale behind assignments of variables to certain categories In case of 1-step approach, interpretation of resulting clusters may be demanding, as they are described by scores of many PCs/Fs
**Principal component analysis <PCA> vs. Exploratory factor analysis <EFA>**	
The most effective method of data reduction (in our study, no arbitrary deletion of variables required) Mathematically more suitable for further use in cluster analysis (component scores calculated, not estimated) More discriminative method (smaller number of eventual clusters)	Requires disjunctive assignment of variables to categories which is problematic in case of conflict properties (according to the PA conflict conceptual framework, they often refer to interactions of many determinants) The total KMO values are lower Less useful for exploring the underlying structure of conflict determinants (in our study, sharp separation of variables assigned to different categories) Consequently, offers less in-depth interpretation of components (e.g., the ‘ageing’ factor turned out to be related to lower employment in the construction sector and better standards of living, which were represented by variables classified as ‘economic’ in the source data register)

In summary, our most general heuristics are as follows:

-  <*z-sc.*> versions of the analysis did not largely contribute to the interpretation of the overall results; we suggest skipping them if the dataset contains variables with positive and negative values.
- If the number of variables is not too high, it is necessary to perform <*μ&a*> analyses. As PA conflicts are defined as processes^19^, their processual dimensions are required to be included.
-  <*μ only*> analyses should be performed to verify the coherence of the entire dataset.
-  <*pre-def.*> analyses should be performed in cases of visible imbalance in the number of variables across groups of determinants. In addition, they allow for better insight into the structure of the results and, if decided, to generate more specific results.
-  <*all var. EFA*> is suggested to verify the underlying structure of the whole dataset.
- For <*pre-def. EFA*> we suggest using PCA with variables not restricted to only one group of determinants. As this results in a non-orthogonal character of <*1-step*> PCs, we suggest further performance of <*2-step pre-def. EFA*> to obtain reliable factor scores.
- For a classic <*pre-def. PCA*> , if only the results of <*1-step*> version are explicable,  <*2-step PCA*> can be skipped, as it only reduces the total explained variance.
- Our approach to unit clustering, i.e., synthesising results from all the cluster analyses, seems to be the most objective, particularly when the results are intended to work as a basis for case study selection.

#### Cross-scale case study simulation assessment

For a case study analysis, we sought a cluster in which a specific legal designation of Polish protected areas played a crucial role in the cluster’s data-driven description. This was only the case for the cluster ‘4a’ (Table 2), which was characterised primarily by a large coverage of national parks in the clustered municipalities, variables related to mass tourism and a high rate of private forest removals. Within the cluster there are two national parks, but only the Tatra National Park intersects four out of five clustered municipalities (for a sketch map, see Supplementary Fig. S1.2 online). Thus, it was selected as a single tenure unit for validation triangulation. The Park protects the only high-mountain range in Poland^73^—the Tatra Mountains—its natural processes, specific habitats and species (including endemic and relict ones), and remnants of human-nature relationships, such as pastoral glades and manufactured legacies^74^.

Based on the characteristics of the cluster, PA conflict analysts could expect at least two groups of conflicts around Tatra National Park. Both conflict potentials are reflected in public statistics collected within a parallel spatio-institutional scale of analysis: the Park ranks first in terms of the number of visitors per year (4.8 million visitors in 2022^75^, 26% of the total for all 23 Polish NPs in 2020)^76^ and contains ca. 15% of non-state-owned land, mainly forests^74^, which is unusual for Polish national parks (for more details, see Supplementary Information 1C online). Triangulation validation based on Internet content analysis not only confirmed that these potentials translate into actual conflict issues around the Tatra NP, but also revealed that the first group of conflicts (tourism-related ones) was the most frequently reported in this secondary data reference source (for a complete list of codes, see Supplementary Table S6.1 online). The content of the Google search^©^ also allowed us to explore the conflicts and recognise the diversity of the tourism stakeholders involved. For 2007–2020, these were, among others:

- Alpine skiers and skiing industry (33% of all relevant records)

Most of these conflicts concern the functioning of the cable car and a network of ski runs in the core of the TNP strict protection zone—Kasprowy Wierch (see Supplementary Fig. S1.2 online). For decades, there has been pressure to develop the complex, which is opposed by the NP managers and environmentalists. In recent years, the following actions were postulated^77^: increase cable car capacity, opening the slopes for off-track skiing (both finally accepted under certain conditions), building a tunnel across Kasprowy Wierch, and building a water reservoir which would allow for snowing the ski runs. In addition, there were disputes over privatisation of the state-owned cable car and the property rights of the space that it traverses.

- Polish Tourist and Sightseeing Society (abbreviated *PTTK*) (12%)

PTTK is a legal heir of the Tatra Society (later, Polish Tatra Society), which fought for establishment of TNP from the end of the nineteenth century. Among others, they purchased the most valuable land for conservation purposes. Furthermore, for decades, PTTK has supervised and gained profits from the leasing of mountain huts located in the TNP. In fact, not all the huts were located on PTTK properties, while the Society remained co-owners of approximately 5% of TNP lands even after its ultimate establishment in 1955 (see Supplementary Fig. S1.2 online). The prolonged dispute ended in 2020 with an agreement between PTTK and TNP on the exchange of properties.

- Providers of fiacre transport services for visitors to TNP (9%)

Fiacre transport services are allowed^78^ on the most popular 8-km tourist road in TNP leading to Morskie Oko, the largest lake in the Tatra Mountains (see Supplementary Fig. S1.2 online). It is supposed to maintain a tradition of past horse transport in the Tatras and to provide maintenance for several local families providing the services^79^. It also allows access to Morskie Oko for those who are unable to reach the lake on foot. However, in recent years, a few horses working on the road collapsed, which sparked intense protests from animal rights activists^79^. Currently, the idea of equipping horse-drawn vehicles with electric support is being considered; however, it is still not embraced by all stakeholders.

- Climbers (5%)

Although climbing is allowed in the eastern part of the TNP (High Tatras), its western part (Western Tatras; see Supplementary Fig. S1.2 online) is almost entirely off limits for climbers^74^. The two Tatra subregions differ in terms of their geological structures and the prevalent genetic types of relief, which aggravates the climbers’ pressure on the Western Tatras (for example, the long limestone rock walls are located only in the Western Tatras). At the same time, the high geological diversity of the Western Tatras is reflected in their exceptional biodiversity, which is assessed as one of the highest in the country^80^. This is used as an argument for the TNP managers against opening the Western Tatras for climbing.

- Ski touring practitioners (3%)

Ski touring is allowed in TNP only along the hiking trails or within a ski complex of Kasprowy Wierch^74^. In the first case, the rule is often violated as skiers tend to choose unmarked slopes for downhill skiing. This, in turn, puts a negative pressure on the fauna of the Tatras. Conversely, conflicts on Kasprowy Wierch engage skiers ascending the slope and those using the slope for descents only, as the space available for both groups is restricted by the TNP^81^.

- Event tourists and organisers (2%)

For the last few years, one of the main New Year’s Eve events held by the public broadcaster Polish Television has been organised in the town of Zakopane, ~ 2 km from the borders of TNP. However, in 2019, the concert was originally planned to be moved to a ski jumping hill of Wielka Krokiew, which is located adjacent to the park borders (see Supplementary Fig. S1.2 online). The TNP managers opposed this plan, arguing for the welfare of local fauna. Finally, the event was held at the original location.

In case of the second group of conflict potentials—related to private forest removals—the Google search^©^ content confirmed their existence, but also revealed a low impact of this issue on the overall Tatra NP conflict image (2% of all relevant records). At the same time, the qualitative insight into the analysed Internet content allowed for better addressing this conflict potential. Although from a legal perspective, TNP managers supervise all forests within the park borders, 16% of these forests are managed by the Forest Community of 8 Legitimate Villages in Witów (see Supplementary Fig. S1.2 online)^74^. The practice of forest management on the community’s land remains questionable, and its complexes work as timberlands rather than protected forests^82^. The most visible difference in forest treatment between the two properties was observed after extensive treefalls in the TNP in 2013. While the community removed dead wood and clear-cut the disturbed surfaces on its land, TNP managers preferred to leave treefall remnants for natural forest succession processes (please note similarities with the conflict over other Polish Man-Biosphere Białowieża Forest^58^). For a few years after the treefall, total forest removals in TNP have remained the highest of all Polish national parks^83^, while private forest management practises in TNP have negatively impacted the perception of the landscape of these lands^82^. At the same time, the head of TNP publicly declares that there is no conflict with the community. This suggests that the proposed indicator-driven analysis allows not only for the identification of open conflicts (tourist conflicts in the Tatra NP), but also the ‘underlying conflict’^84^ layers (a conflict potential over excessive private forest removals).

### Applications and limitations of the approach

Our study offers a universal method of integrating official statistics into the multi-level process of PA conflict analysis. The ultimate application of the approach is to serve as a basis for informed case study selection for further in-depth enquiries. Responding to the cross-disciplinary problem of clashing epistemologies in the era of datafication^1,26^, we proposed a rigorous three-fold method of secondary data assessment and triangulation validation that can serve as a benchmark in many fields of social sciences. Also, we have shown how to reduce a multivariate set of PA conflict determinants^14^ while maintaining their spatial consistency^18,19^. We believe this approach is particularly useful in the context of global policy ambitions to increase the number of new PA designations, expand existing ones^85^, and promote inclusive conservation^86^. Based on experiences with the implementation of the Natura 2000 network^63,87,88^, such processes may pose new challenges on policy-makers and managers working at the scale of a PA network who already face difficulties with limited access to PA stakeholders and misinformation^89^. Their access to reliable data from the conflict identification and analysis stages is a necessary input for effective conservation conflict management^90^, and our approach helps to maintain the reliability of the data provided even in crisis conditions.

At the same time, we acknowledge that the regional scale of our study implies certain limitations that researchers in other geographical contexts may face. First, PA conflicts are context-specific^14,84,91^ and the list of conflict factors relevant for indicator analysis may differ across locations. To address this, we suggest applying the entire procedure to the other research contexts, including a systematic review of PA conflict literature (Fig. 1, step A) to identify regionally relevant conflict factors. Second, the results of a clustering procedure always depend on the data availability, which differs across the state registers. In terms of data coverage and openness, the database selected for this study is considered one of the best in the world^92^. In addition, Statistics Poland is part of the European Statistical System, which obliges it to collect and provide data that are comparable at the EU level^93^. At the same time, despite global efforts to broaden the range of indicators that can describe human well-being^94^, individual-level psychological measures are still largely missing across state registers. Furthermore, a detected bias toward a greater availability of economic data compared to social and environmental statistics is observed worldwide^95^. This makes our warnings against using such registers to address PA conflicts per se even stronger and more general. To address the data availability challenges, we suggest conducting a thorough theory-driven inspection of a dataset (Fig. 1, step k) before using it to inform the selection of case studies. As the assessment results may differ depending on the choice of the reference conceptual framework, we recommend the use of integrative and multidimensional models.

Our method of data reduction and clustering has already been used in the field of conservation and land-use conflicts, both for survey^96,97^ and for spatio-temporal analysis^98^. However, the procedures involve some arbitrary decisions^43,99^ that always affect the resulting outcomes. Our approach of synthesising the results of separately conducted procedures is one way of mitigating this effect and fits into the recent trend in socio-ecological system analysis^100^. However, most indicator-driven approaches to conservation conflict analysis are too reductionist for the purpose of our study, as they simply juxtapose biodiversity with socio-economic indicators^101^ or do not use them to classify local-level units^102,103^. Quantitative approaches to case selection require independent recognition of the diversity of all relevant variables^104^ and our method not only allows for a systematic reduction of these variables, but it also preserves information about their spatial diversity. Although we used a purposive criterion for our case selection, the clustering results enable the application of cross-case methods, such as case similarity (a set of PAs from the same cluster) or diversity (a set of PAs intersecting more than one cluster)^104^.

Finally, proper interpretation of the last part of our assessment approach requires understanding of the relationships between the two sets of data analysed. Our triangulation validation assumed juxtaposing the indicator-based results with secondary data that not only adhere to different scales of analysis (spatio-institutional and spatio-administrative) but also belong to different epistemologies. As such, the results of these two distinctive data sources should never be directly compared. Rather, the purpose of the validation phase was to check whether the conflict potentials derived from the description of the cluster were reflected in any way in the conflict reports. The selected case study proved useful for the validation, as the Tatra National Park is widely covered in the Polish media. However, even if the validation results for another PA were not so straightforward, this would not preclude the usefulness of the clustering approach for in-depth analysis. Some conflicts may remain latent and not widely reported^84^, but can still be explored using cluster descriptions, which we managed to present for private forest removals in the TNP. Alternatively, some conflict can be captured using another constructivist type of secondary data for validation purposes, such as user-generated social media content^24,105^.

## Conclusions

In some research fields, such as PA conflict analysis, the use of constructionist methodologies is necessary to uncover the truth claims of multiple stakeholders^21^. However, during crisis conditions, such as the recent COVID-19 pandemic, proxy approaches must maintain a scientific response to ongoing socio-environmental challenges. With a myriad of secondary data available in the age of datafication^6^, careful data triangulation^13^ requires a thorough insight into the data characteristics, including their theoretical, scalar, and epistemological coherence, to ensure the validity of the results. Our multi-faceted assessment of the official state statistics register has demonstrated its potential as a proxy for a multi-level analysis of PA conflict determinant, conflict potential identification, and case study selection. However, there are a number of lessons learnt from this study that researchers and practitioners should be aware of:

1. Data that do not contain direct input from conflict stakeholders^14^ should not be interpreted as conflict-related data per se. Therefore, a minimum necessary insight into the perception frames of stakeholders must be retained, even in crisis conditions such as the COVID-19 pandemic.
2. The usefulness assessment of secondary data should always be guided by a comprehensive conceptual framework that can be applied across a range of methodological approaches and techniques. This approach helps to evaluate the completeness of the dataset and legitimises the interpretation of the data.
3. The results of public statistics indicator analysis are sensitive to the applied analytical methods. Iterations of the process using different analytical approaches should be standard practise, while a synthetic approach is recommended to achieve greater objectivity in the selection of case studies.
4. To validate the process, the results of indicator-driven analyses should always be related to other types of secondary data (e.g., media reports available on the Internet).

## Supplementary Information

Supplementary Information.

## Data Availability

The datasets generated during and/or analysed during the current study are partially presented in the Supplementary Information online. The remaining datasets are available from the corresponding author on reasonable request.
