# Supplementary material for: A critical assessment of a protected area conflict analysis based on secondary data in the age of datafication
Source: Sci Rep. 2023 May 17;13:8028. doi: 10.1038/s41598-023-35067-x (PMC10191084; doi:10.1038/s41598-023-35067-x)
Supplement: Supplementary file 1 — Supplementary Information. [file 41598_2023_35067_MOESM1_ESM.pdf]

Does secondary data suffice? Protected area conflict analysis in the era of the COVID-19 pandemic

Marcin Rechciński[1], Joanna Tusznio, Arash Akhshik, Małgorzata Grodzińska-Jurczak

[1] corresponding author; Jagiellonian University in Krakow, Faculty of Geography and Geology; Institute of Geography and Spatial Management; Gronostajowa 7, 30-387 Krakow, Poland, marcin.rechcinski@uj.edu.pl

Jagiellonian University in Krakow, Faculty of Biology; Institute of Environmental Sciences, Gronostajowa 7, 30-387 Krakow, Poland,

Supplementary Information 1

A. LESSER POLAND as a study area

A complete rationale for the selection of the region (see also Fig. S1.1):

- Lesser Poland is one of the only three voivodeships of Poland located in two biogeographical regions (continental and alpine one; European Commission 2022), with the highest number of national parks (6) in a whole country (Generalna Dyrekcja Ochrony Środowiska 2022)
- Protected areas of all eight legal designations from the Nature Conservation Act (2004) (see tab. S.1.1. below for their description) are located in the region (Generalna Dyrekcja Ochrony Środowiska 2022), which diversifies conditions for potential PA conflicts,
- The landscape of Lesser Poland is the most diverse in a whole country and consists of two uplands of different geological structures, Subcarpathian river basin of Vistula, highlands, and high mountains of the Carpathians (Solon et al. 2018),
- Four cultural lands of different past state belongingness and ethnic continuity can be distinguished in Lesser Poland (Plit 2015), which still diversifies many socio-cultural, institutional, and economic determinants of PA conflicts.

Many socio-economic characteristics of the region are still shaped by its complex geopolitical history. During the period of Partitions of Poland (1795-1918), Lesser Poland was divided into two parts belonging to Tsarist Russia (norther part) and Austrian (later – Austro-Hungarian) Empire (southern part of the region, formerly named as Galicia) (Śleszyński, Gibas and Sudra 2020). In both partitions, it was emancipation of peasants which started parcellation of large agricultural properties; however, in Galicia the process was not accompanied with any rules of land turnover (Kieniewicz 1969). This led to several subdivisions of already small mountainous holdings, making them unable to provide any subsistence. This induced the entrepreneurship of inhabitants, forced to seek non-agricultural sources of income (Rosner and Stanny 2017). The situation has not changed much during the Communist period, as the region successfully defended against the then-performed land collectivisation (Bucała-Hrabia 2018). However, a large share of scattered private lands contributed to many past conflicts connected with establishment of protected areas, as the processes were often preceded by compulsory land expropriation for conservation purposes (Grodzinska-Jurczak and Cent 2011, Brown et al. 2015).

The city of Kraków is largely different from the rest of Lesser Poland. This is the only city in Lesser Poland classified as a separate NUTS 3 unit (Statistics Poland 2022) with more than seven times more inhabitants than the second most populated one (Statistics Poland 2021).

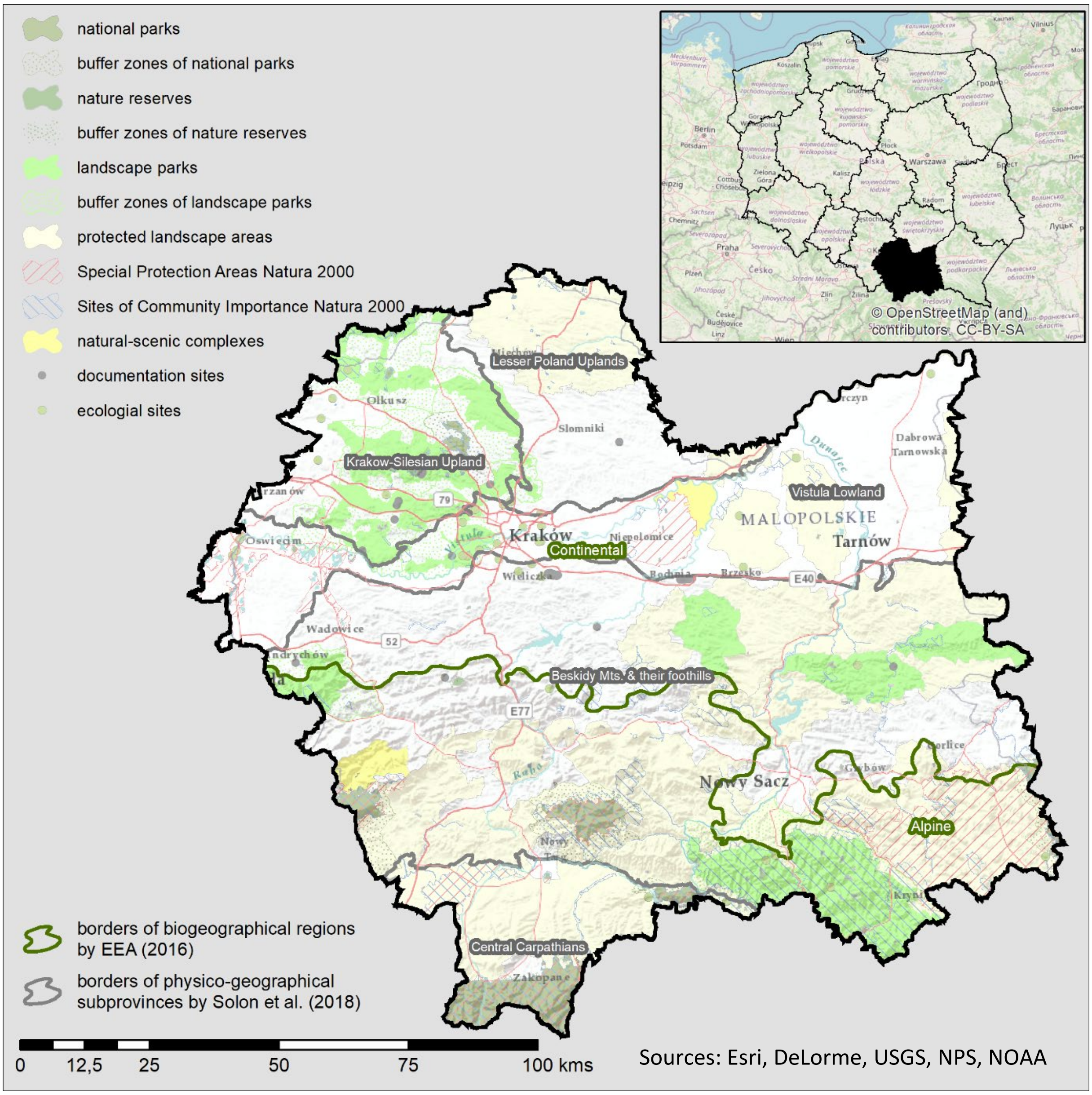

Fig. S1.1 An overview map of a Lesser Poland voivodeship (NUTS-2) with indicated a system of protected areas, borders of physico-geographical subprovinces and borders of biogeographical regions

B.

Tab. S1.1. Legal designations of protected areas in Lesser Poland, according to the Nature Conservation Act (2004)

**Legal designations**	**Conservation regime**	**IUCN cat. (UNEP-WCMC and IUCN 2022)**	**Level on a jurisdictional scale, according to Cash et al. (2006)**	**Level on a jurisdictional scale, according to Cash et al. (2006)**	**Number of PAs in a Lesser Poland voivodeship (Regionalna Dyrekcja Ochrony Środowiska w Krakowie 2022)**
National parks	High	Mostly II	National	Regional	6
Nature reserves	High	IV	National - regional	Landscape / patch	86
Landscape parks	Moderate	V	Regional	Regional	11
Protected Landscape Areas	Low	Not Assigned	Regional	Regional	10
Natura 2000 sites	Mixed	Not Reported	Multi-level (European - local)	Mixed (regional - patch)	2 PLC, 9 PLB, 86 PLH
Documentation sites	Low	Not Reported	Local	Ecological patch	80
Ecological sites	Low	Not Reported	Local	Ecological patch	43
Nature-scenic complexes	Low	Not Reported	Local	Landscape	6

C. Tatra National Park municipalities as a case study

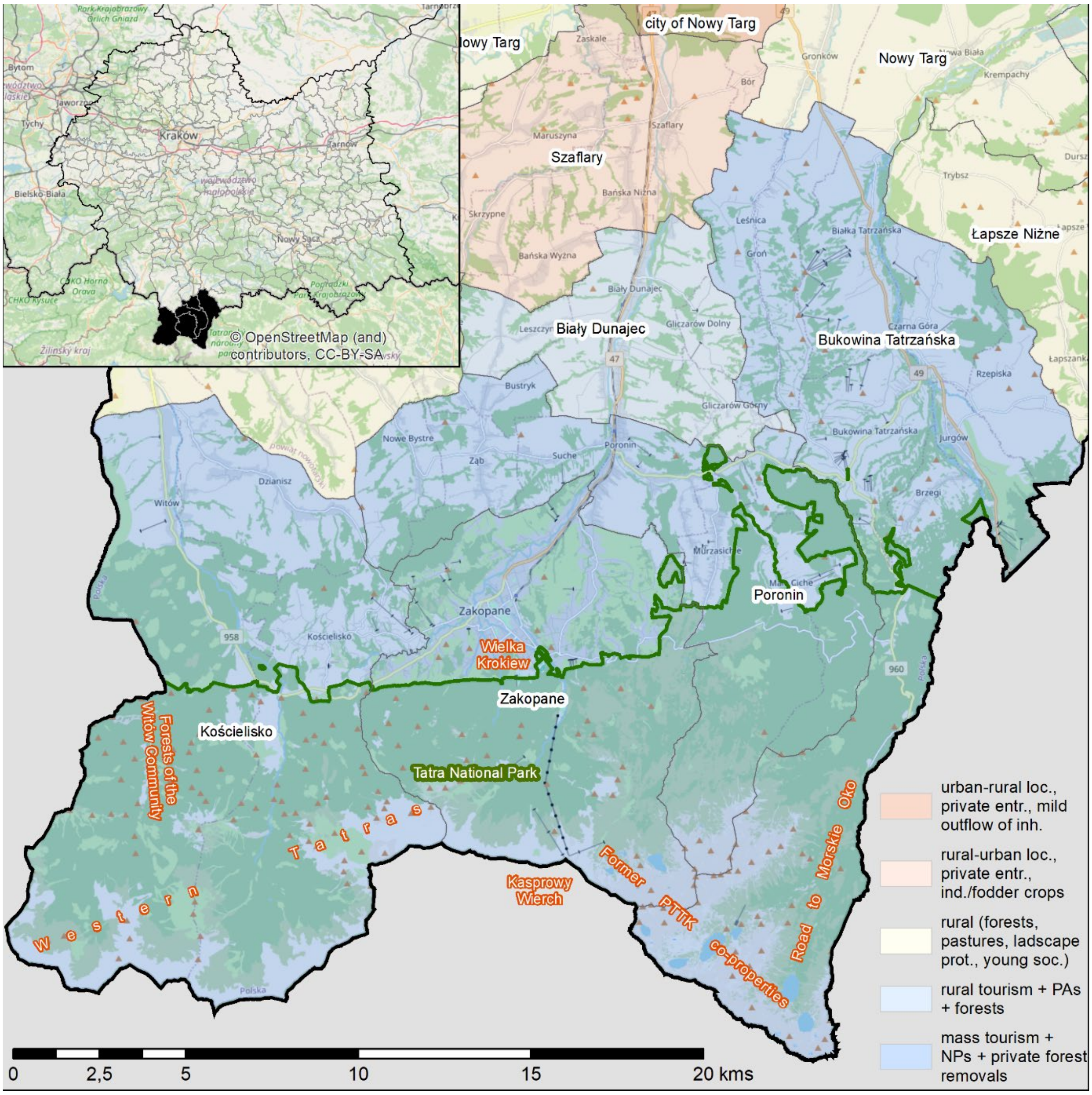

Fig. S1.2 An overview map of the Tatra poviat (NUTS-3), selected for a case study assessment. The poviat was clurested as a whole into 'tourist' clusters (4a – Kościelisko, Zakopane, Poronin, Bukowina Tatrzanska; 4b – Biały Dunajec). Red labels represent selected sub-local conflict issues that were addressed in the analysed material.

Tatra National Park contains ca. 15% of non-state treasury land, which are mainly forests. This is unusual for Polish national parks, even though a total share of private lands in Polish national parks seems similar (13%, Sejm Committees' Bureau 2020): the total share is strongly influenced by a structure of lands in the largest national park in Poland (Biebrza NP, outside of Małopolska), where 36% of lands, mostly agricultural, are private (Biebrzański Park Narodowy 2019).

Supplementary Information 2 – systematic literature review on PA conflicts in Poland

For the purposes of this study, we used and expanded a search from (Rechciński, Grodzińska-Jurczak and Tusznio 2018). The search was performed in Web of Science – ISI Web of Knowledge, using an Advanced Search option and the following set of search keywords:

TS =(conservation OR biodiversity OR wildlife OR human-wildlife OR „protected area*" OR „national park*" OR „reserve" OR „reserves" OR landscape* OR Natura 2000 OR complex* OR ecologic* OR site*)

AND TS =(conflict* OR dispute*)

AND TS =Poland

The results were limited to the time frame of 2007-2020. As the search was aimed at capturing only PA conflicts, all the abstracts were scanned in search of papers that referred to any legal designation of the Polish PAs. Finally, most of the search results (188) were classified as non-relevant. The final list of manuscripts used for further analysis is presented in Tab. S2.1.

Tab. S2.1. List of scientific articles on protected area conflicts in Poland, published between 2007 and 2020 in scientific journals of international recognition

No.	Authors and year of publication	Title of the publication	Journal title (IF in year of publication)	Spatial level of analysis declared by authors
1	(Grodzinska-Jurczak and Cent 2011)	Expansion of Nature Conservation Areas: Problems with Natura 2000 Implementation in Poland?	*Environmental Management* (1,503)	national (based on case studies)
2	(Olko et al. 2011)	Cooperation in the Polish national parks and their neighborhood in a view of different stakeholders - a long way ahead?	*Innovation: The European Journal of Social Science Research* (0,326)	local (case studies)
3	(Niedziałkowski, Paavola and Jędrzejewska 2012b)	Participation and protected areas governance: the impact of changing influence of local authorities on the conservation of the Bialowieza Primeval Forest, Poland	*Ecology and Society* (2,516)	local (a case study)
4	(Kubacka 2012)	The Role of Local Association of Communes in Environmental Management Systems: Selected Case Studies in the Wielkopolska Region	*Polish Journal of Environmental Studies* (0,508)	regional (based on case studies)
5	(Niedziałkowski, Paavola and Jędrzejewska 2012a)	Governance of biodiversity in Poland before and after the accession to the EU: the tale of two roads	*Environmental Conservation* (2,341)	national (based on case studies)
6	(Niedziałkowski et al. 2014)	Why is it difficult to enlarge a protected area? Ecosystem services perspective on the conflict around the extension of the Bialowieza National Park in Poland	*Land Use Policy* (3,134)	local (a case study)
7	(Bielecka and Różyński 2014)	Management conflicts in the Vistula Lagoon area	*Ocean & Coastal Management* (1,769)	local (a case study)
8	(Chmielewski and Głogowska 2015)	Implementation of the Natura 2000 Network in Poland - an Opportunity or a Threat to Sustainable Development of Rural Areas? Study on Local Stakeholders' Perception	*Eastern European Countryside* (0,062)	national

9	(Jankowiak et al. 2015)	Patterns of occurrence and abundance of roosting geese: the role of spatial scale for site selection and consequences for conservation	*Ecological Research* (1,296)	national
10	(Paloniemi et al. 2015)	Public Participation and Environmental Justice in Biodiversity Governance in Finland, Greece, Poland and the UK	*Environmental Policy and Governance* (1,614)	international
11	(Brown et al. 2015)	Cross-cultural values and management preferences in protected areas of Norway and Poland	*Journal for Nature Conservation* (1,646)	international (based on case studies)
12	(Kubacka and Macias 2016)	The functioning of Natura 2000 areas in the opinion of different groups from the local community: A case study from Poland	*Society & Natural Resources* (1,758)	local (a case study)
13	(Bernat 2016)	Analysis of Social Conflicts in Poland's Soundscape as a Challenge to Socio-Acoustics	*Archives of Acoustics* (0,661)	national
14	(Piwowarczyk and Wróbel 2016)	Determinants of legitimate governance of marine Natura 2000 sites in a post-transition European Union country: A case study of Puck Bay, Poland	*Marine Policy* (2,453)	local (a case study)
15	(Logmani et al. 2017)	Customizing elements of the International Forest Regime Complex in Poland? Non-implementation of a National Forest Programme and redefined transposition of NATURA 2000 in Bialowieza Forest	*Forest Policy and Economics* (1,982)	national (based on a case study)
16	(Wiątkowski, Rosik-Dulewska and Tomczyk 2017)	Hydropower structures in the Natura 2000 site on the River Radew: an analysis in the context of sustainable water management	*Rocznik Ochrona Srodowiska* (0,705)	local (a case study)
17	(Maczka et al. 2019)	The ecosystem services concept as a tool for public participation in management of Poland's Natura 2000 network	*Ecosystem Services* (6,330)	national
18	(Wasilewski, Szulczewska and Giedych 2019)	Visitors' Perception of Urban Nature Reserves in Poland	*Sustainability* (2,576)	national
19	(Borkowski et al. 2019)	High density of keystone herbivore vs. conservation of natural resources: Factors affecting red deer distribution and impact on vegetation in Słowiński National Park, Poland	*Forest Ecology and Management* (3,170)	local (a case study)

20	(Wilkaniec et al. 2020)	Urbanisation processes in Puszcza Zielonka landscape park in Poland – and its buffer zone in the context of protection of natural structures	*Applied Ecology and Environmental Research (0,711)*	local (a case study)
21	(Zawilińska 2020)	Residents' Attitudes Towards a National Park Under Conditions of Suburbanisation and Tourism Pressure: A Case Study of Ojców National Park (Poland)	*European Countryside (0,460)*	local (a case study)
22	(Strzelecka et al. 2020)	Resident Perceptions of Distribution, Recognition and Representation Justice Domains of Environmental Policy-Making: The Case of European Ecological Network Natura 2000 in Poland	*Society & Natural Resources (2,821)*	local (case studies)
23	(Kot-Niewiadomska and Pawłowska 2020)	The Possibilities of Open-Cast Mining in Landscape Parks in Poland—A Case Study	*Resources (0,480)*	multi-level (European – national – local)

[1] The full list of authors has been included in the references.

Supplementary Information 3 – PA conflicts in Poland: factors and variables

Tab. S3.1. Factors of PA conflicts accompanied by local-level variables representing the factors obtained from the Local Data Bank of Statistics Poland [accessed July-September 2021]

PA conflict factors	Source for the factor choice	No.[1]	Local-level variables[2]	Source for the variables choice[2]	Data subgroup. Code in LDB	Comments
Socio-economic development of a municipality	(Grodzinska-Jurczak and Cent 2011, Olko et al. 2011, Niedziałkowski, Paavola and Jędrzejewska 2012b, Bielecka and Różyński 2014, Chmielewski and Głogowska 2015)<br><br>Economic development only (Niedziałkowski, Paavola and Jędrzejewska 2012a, Niedziałkowski et al. 2014, Strzelecka et al. 2020)	1.	Share of own revenues of gmina budgets in the total revenue of gmina budgts	(Józefowicz and Michniewicz-Ankiersztajn 2020)	2622/2621	
		10.	Expenditures of gminas budgets per capita	(Ociepa-Kicińska 2019)	2644	
		28.<br>29.	persons using:<br>• water supply system<br>• sewage system<br>in % of total population	(Chrzanowska and Drejerska 2016)	2433	
		31.	Natural persons conducting economic activity per 100 thous. persons of working age	(Józefowicz and Michniewicz-Ankiersztajn 2020, Chrzanowska and Drejerska 2016)	2419	
		32.	Entities entered in the REGON register per 10 thous. population.	(Ociepa-Kicińska 2019)	2419	
		33.	foundations, associations and social organizations per 10 thous. population	(Chrzanowska and Drejerska 2016)	2419	
		51.	private sector - commercial companies without foreing capital participation per 1000 persons	(Chrzanowska and Drejerska 2016)	((3822 – 2822)/2914) * 1000 inh	
		52.	private sector - commercial companies with foreign capital participation per 1000 persons	(Chrzanowska and Drejerska 2016)	(2822/2914) * 1000 inh	
		59.	dwellings completed per 10 thous. population	(Chrzanowska and Drejerska 2016)	3820	
		85.	Total net migration per 1000 population	(Ociepa-Kicińska 2019)	1350	

		87.	Demographic dependency ratio (post-working age population per 100 persons of working age)	(Chrzanowska and Drejerska 2016)	2426	
		90.	natural increase per 1000 population	(Ociepa-Kicińska 2019)	3428	
		97.	Persons over 65 in % of total population	(Ociepa-Kicińska 2019)	3447	
		102.	beneficiaries of social assistance per 10 thous. of population	(Józefowicz and Michniewicz-Ankiersztajn 2020)	3870	
		103.	Share of the registered unemployed persons in the population in the working age	(Józefowicz and Michniewicz-Ankiersztajn 2020, Chrzanowska and Drejerska 2016, Ociepa-Kicińska 2019)	2670	
		107.	Share of children aged 3-5 years covered by preschool education	(Chrzanowska and Drejerska 2016)	2958	
Agricultural development of a municipality	(Grodzinska-Jurczak and Cent 2011, Niedziałkowski et al. 2014)	2.	Share of agricultural tax in own revenue of gmina budgets	(Majchrzak 2008)	2622/2622	No data on agricultural conditions of private farms for their size groups in the time section<br>No data on average area of private farms in the time section<br>There are no precise data on the structure of income in private farms in the time section<br>No data on combines at the local level<br>No data on employment in agriculture at the local level<br>No data on structure of expenditures in agriculture at the local level
		3.	Agricultural tax per capita	(Szmytkie and Tomczak 2016)	2622/2914	
		149.	Tractors in private farms per 100 ha agricultural lands	(Majchrzak 2008)	(3024/3020)* 100 ha	
		161.	Share of income from agricultural activities in a total income of private farms	(Majchrzak 2008)	3359	
		162.	Share of income from retirements and other pensions in a total income of private farms	(Majchrzak 2008)	3359	
		141.	Share of private farms in an area group of 15 ha and more in a total of private farms	(Majchrzak 2008)	3019	
		123.	Forest cover in per cent	(Majchrzak 2008)	1591	
		124.	Agricultural lands per 1 inhabitant	(Majchrzak 2008)	2779/2914	

		125.	Share of agricultural lands in a total area of a municipality	(Majchrzak 2008, Szmytkie and Tomczak 2016)	2779	No data on farmer education<br>No data on commodity agriculture at the local level<br>No data referring to areas of agricultural land were included due to errors found in the dataset<br>No longitudinal data on variables 141, 149, 157, 158<br>The data refer to agricultural holdings based in the gmina (with their lands possibly located outside)
		144.	Share of agricultural lands in a total area of private farms	(Majchrzak 2008)	3020	
Wealth of a locality/region	(Niedziałkowski et al. 2014, Grodzinska-Jurczak and Cent 2011, Bielecka and Różyński 2014, Kubacka and Macias 2016, Strzelecka et al. 2020)	1.	Share of own revenues of gmina budgets in the total revenue of gmina budgts	(Kozera, Głowicka-Wołoszyn and Wysocki 2015, Hok 2015)	2622/2621	No GDP per capita data at the local level
		4.	Share of targeted grants and general subventions in a total revenue of gmina budgets	(Kozera et al. 2015, Hok 2015)	(2624 + 2623) / 2621	
		6.	Own revenue of gminas per capita	(Binda 2020, Kozera et al. 2015, Hok 2015)	2627	
		8.	share of investment expenditure in total gminas expenditure	(Kozera et al. 2015, Hok 2015)	2633	
		11.	public debt servicing per capita	(Hok 2015)	2919/2914	
Financial support of national government to local authorities	For sustainable development -- (Olko et al. 2011, Niedziałkowski et al. 2014, Niedziałkowski et al. 2012b)	5.	Targeted grants received from the state budget for own tasks per capita		2624/2914	
Wealth of a community, economic well-being asymmetries of wealth	(Paloniemi et al. 2015, Piwowarczyk and Wróbel 2016, Grodzinska-Jurczak and Cent 2011, Olko et al. 2011, Bielecka and Różyński 2014)	7.	Revenue per capita	(Michalska-Żyła 2016, Kędzierska et al. 2013, Miłek 2018)	2627	No data on asymmetries of wealth within municipalities<br>No data on persons entitled to unemployment benefit at the local level
		9.	Share of expenditure in public roads in the total expenditure	(Miłek 2018)	3305	
		12.	share of gminas expenditures on health care in total gminas expenditures	(Michalska-Żyła 2016)	2920	

		13.	share of gminas expenditures on municipal economy and environmental protection in total gminas expenditures	(Michalska-Żyła 2016)	2920	
		19.	No of dwellings in gminas' stock with arrears with payments over 3 months per total number of dwellings in gmina	(Kędzierska et al. 2013)	1731/2166	
		20.	Number of dwellning allowances paid to the users per 100 inh	(Kędzierska et al. 2013)	(2173/2914) * 100 inh	
		21.	Average useful floor area per 1 person	(Kędzierska et al. 2013, Miłek 2018)	2430	
		22.	dwelling stock per 1000 population	(Miłek 2018)	2430	
		23.<br>24.<br>25.<br>26.<br>27.	Share of dwellings fitted with installations:<br>• Water supply system<br>• Flushing lavtory<br>• Bathroom<br>• Central heating<br>• Gas from gas supply system	(Kędzierska et al. 2013)	2431	
		28.<br>29.<br>30.	persons using:<br>• water supply system<br>• sewage system<br>• gas system<br>in % of total population	(Miłek 2018)	2433	
		32.	Entities entered in the REGON register per 10 thous. population.	(Miłek 2018)	2419	
		78.	population per library	(Miłek 2018)	2381	
		79.	public libraries borrowers per 1000 population	(Miłek 2018)	2381	
		80.	library loans per borrower in vol.	(Miłek 2018)	2381	
		85.	Total net migration per 1000 population	(Miłek 2018)	1350	

		87.	Demographic dependency ratio (post-working age population per 100 persons of working age	(Miłek 2018)	2426	
		91.	Live births per 1000 population	(Miłek 2018)	3428	
		92.	Deaths per 1000 population	(Miłek 2018)	3428	
		98.	health out-patient departments per 10 thous. population	(Miłek 2018)	2011	
		99.	generally available pharmacies per 10 thous. population	(Miłek 2018)	2434	
		100.	Share of children aged under 17 years, for which the parents shall receive family allowances in the total number of children in this age	(Kędzierska et al. 2013, Miłek 2018)	2992	
		101.	beneficiaries of social assistance under criterion of income per 10 thous. of population	(Miłek 2018)	(3538/2914)* 10 000 inh	
		102.	beneficiaries of social assistance per 10 thous. of population	(Michalska-Żyła 2016, Miłek 2018, Kędzierska et al. 2013)	3870	
		103.	Share of the registered unemployed persons in the population in the working age	(Kędzierska et al. 2013, Miłek 2018)	2670	
		104.	Employed persons per 1000 population	(Michalska-Żyła 2016, Miłek 2018)	3469	
		105.	Gross education ratio – primary schools	(Kędzierska et al. 2013, Miłek 2018)	2387	
		106.	Pupils per section in primary schools	(Miłek 2018)	3463	
		107.	share of children aged 3-5 years covered by preschool education	(Michalska-Żyła 2016, Miłek 2018)	2958	
		108.	places in nursery schools per 1000 children aged from 3 to 6 years	(Miłek 2018)	3534	
		183.	Treated wastewater per 1000 population	(Miłek 2018)	(1700/2914)* 1000 inh	

		184.	Share of population connected to wastewater treatment plants	(Miłek 2018)	1702/2914	
		187.	share of parks, lawns and green areas of the housing estate areas in the total area	(Miłek 2018)	3303	
Metropolitan areas	(Kubacka 2012)	6.	Own revenue of gminas per capita	(Smętkowski, Jałowiecki and Gorzelak 2008)	2627	Official data not available in the dataset. Only criteria for delimitation of metropolitan areas are available at the local level.<br><br>No data on the average distance to the capital of the metropolitan area
		52.	private sector - commercial companies with foreign capital participation per 1000 persons	(Smętkowski et al. 2008)	(2822/2914) * 1000 inh	
		51.	private sector - commercial companies without foreign capital participation per 1000 persons	(Smętkowski et al. 2008)	((2822 – 2822)/2914) * 1000 inh	
		85.	Total net migration per 1000 population	(Smętkowski et al. 2008)	1350	
Public investments	In infrastructure --- (Olko et al. 2011, Niedziałkowski et al. 2012a, Bielecka and Różyński 2014, Chmielewski and Głogowska 2015, Kubacka and Macias 2016)	9.	Share of expenditure in public roads in the total expenditure	(Śleszyński et al. 2018)	3305	No data on average plot size of the investments
		117.	decisions on the location of public purpose investment per 1000 population	(Śleszyński et al. 2018)	(2851/2914)* 1000 inh	
Priorities of authorities	On infrastructure development -- (Grodzinska-Jurczak and Cent 2011, Niedziałkowski et al. 2012a)<br><br>On economic development -- (Olko et al. 2011, Niedziałkowski et al. 2012b, Niedziałkowski et al. 2012a)	14.	Share of gminas expenditures on transport and communication		2920	Only proxy data concerning infrastructure available
Rise in costs of nature protection	(Grodzinska-Jurczak and Cent 2011, Paloniemi et al. 2015, Bernat 2016, Strzelecka et al. 2020)	15.	Share of municipality expenditures on nature protection		2920	
Experience in acquiring the EU funds for the N2000 programme implementation	(Grodzinska-Jurczak and Cent 2011, Kubacka and Macias 2016)	16.	No of grant applications within the OPIE per 10 000 inhabitants		(3660/2914) * 10 000 inh	No precise data on the funds allocation

Availability of the EU funds	For infrastructure development - (Grodzinska-Jurczak and Cent 2011, Niedziałkowski et al. 2012a)<br>For participatory solutions - (Strzelecka et al. 2020)	17.<br>18.	A value of grant contracts (community funds and EIB) within<br>a) OPIE<br>b) OPKED<br>Per inhabitant		3682/2914	No precise data on the funds allocation
Infrastructure development	(Grodzinska-Jurczak and Cent 2011, Olko et al. 2011, Niedziałkowski et al. 2012b, Niedziałkowski et al. 2012a, Chmielewski and Głogowska 2015, Wilkaniec et al. 2020)<br><br>Flood defence infrastructure -- (Grodzinska-Jurczak and Cent 2011)<br><br>Sewage system -- (Grodzinska-Jurczak and Cent 2011, Kubacka and Macias 2016, Wiatkowski, Rosik-Dulewska and Tomczyk 2017)<br><br>Water treatment facilities -- (Bielecka and Różyński 2014)<br><br>Harbour infrastructure -- (Bielecka and Różyński 2014, Piwowarczyk and Wróbel 2016)<br><br>Forest infrastructure -- (Logmani et al. 2017)<br><br>Fishing infrastructure -- (Maczka et al. 2019)	28.<br>29.<br>30.	persons using:<br>• water supply system<br>• sewage system<br>• gas system<br>in % of total population	(Kołodziejczyk 2014, Kałuża and Kałuża 2017, Dziekański 2016)	2433	Based on the context of references to infrastructure in the source papers, the variable has been limited to technical infrastructure only<br><br>No data on harbour/ forest/ fishing infrastructure<br><br>No data on flood embankments, river regulation, and streaming water area for the same time section
		21.	Average useful floor area per 1 person	(Dziekański 2016)	2430	
		184.	Share of population connected to wastewater treatment plants	(Dziekański 2016)	1702/2914	
Water quality	(Bielecka and Różyński 2014, Jankowiak et al. 2015, Wiatkowski et al. 2017)	29.	persons using sewage system in % of total population	(Affek 2013)	2433	Only proxy data available
		178.<br>179.<br>180.<br>181.<br>182.	Pollutant loads in wastewater discharged into waters or into the ground per 1 ha:<br>• biochemical oxygen demand<br>• chemical oxygen demand<br>• total suspension<br>• total nitrogen<br>• total phosphorus		(1979 + 1699)/ 1410	

		184.	Share of population connected to wastewater treatment plants		1702/2914	
		185.	Wastewater treated biologically, chemically and with increased biogene removal in % of wastewater requiring treatment	(Affek 2013)	1984	
Energy infrastructure development	(Grodzinska-Jurczak and Cent 2011)<br>Gas system -- (Kubacka and Macias 2016)<br>Wind farms -- (Bernat 2016)<br>*Hydropower plants* -- (Wiatkowski et al. 2017)	30.	persons using gas system in % of total population	(Kałuża and Kałuża 2017, Dziekański 2016)	2433	Data on electricity consumers refer only to urban citizens; hence, they were not included<br><br>No data on poweplants available
Business activity	(Grodzinska-Jurczak and Cent 2011, Olko et al. 2011, Chmielewski and Głogowska 2015, Kubacka and Macias 2016, Piwowarczyk and Wróbel 2016, Wilkaniec et al. 2020, Niedziałkowski et al. 2014, Bielecka and Różyński 2014, Strzelecka et al. 2020)<br><br>Carpentry -- (Niedziałkowski et al. 2014)<br><br>Guesthouse tourism -- (Bernat 2016)	31.	Natural persons conducting economic activity per 100 thous. persons of working age	(Sołtys and Dorocki 2016)	2419	
		32.	Entities entered in the REGON register per 10 thous. population.	(Barczyk-Ciuła et al. 2018, Szmytkie and Tomczak 2016)	2419	
		38.	A share of entered entities into a 16th division of the NACE rev. 1.2 (PKD 2007) in a total number of the entities entered to the NACE rev. 1.2.		2809	
		61.	Tourist accommodation establishments per 10 th population:<br>• guesthouses<br>(mean data only)		(2017/2914)*<br>10 000 inh	
Tourism development of a municipality	(Grodzinska-Jurczak and Cent 2011, Niedziałkowski et al. 2014, Bielecka and Różyński 2014)	42.	A share of entered entities into a section I of the NACE rev. 1.2 (PKD 2007) in a total number of the entities entered to the NACE rev. 1.2.	(Szmytkie and Tomczak 2016)	2809	No data on employment in tourism at the local level
		62.	Defert's index - Total number of accommodated tourists / 1 km2	(Synówka-Bejenka 2017, Gonia and Podgórski 2019, Warczewska 2017)	2017/1410	

		63.	Schneider's index - Total number of accommodated tourists per 100 inhabitants	(Synówka-Bejenka 2017, Gonia and Podgórski 2019, Warczewska 2017)	(2017/2914)* 100 inh	
		64.	Baretje-Defert's index – Total number of bed places per 100 inhabitatns	(Synówka-Bejenka 2017, Gonia and Podgórski 2019, Warczewska 2017, Szmytkie and Tomczak 2016)	(2017/2914)* 100 inh	
		65.	Total number of bed places / 1 km2	(Synówka-Bejenka 2017, Gonia and Podgórski 2019)	2017/1410	
		66.	Chavart index - Total number of nights spent in tourist accommodation / 100 inhabitatns	(Gonia and Podgórski 2019, Warczewska 2017)	(2017/2914)* 100 inh	
Development of other sectors in a municipality	Industry, fishing -- (Grodzinska-Jurczak and Cent 2011)<br>Transportation -- (Bielecka and Różyński 2014)<br>Energy -- (Bernat 2016)<br>Mineral Industry - (Kot-Niewiadomska and Pawłowska 2020)	36.<br>37.<br>37a<br>39.<br>41.	A share of entered entities into certain sections and divisions of the NACE rev. 1.2 (PKD 2007) in a total number of the entities entered to the NACE rev. 1.2.<br>• Fishing – section A, division 03<br>• Industry – section C (B-F)<br>• Energy – section D<br>• Transportation – section H<br>• Mineral industry – Section B, division 08	(Malinowski et al. 2009)	2809	No data on employment in certain sectors at the local level
Economic sectors	Agriculture -- (Grodzinska-Jurczak and Cent 2011, Niedziałkowski et al. 2012b)<br><br>Fishing -- (Bielecka and Różyński 2014, Piwowarczyk and Wróbel 2016, Wiatkowski et al. 2017, Maczka et al. 2019)<br><br>Tourism -- (Bielecka and Różyński 2014, Piwowarczyk and Wróbel 2016, Grodzinska-Jurczak and Cent 2011, Logmani et al. 2017)	34.<br>35.<br>36.<br>37.<br>39.<br>41.<br>42.	A share of entered entities into certain sections and divisions of the NACE rev. 1.2 (PKD 2007) in a total number of the entities entered to the NACE rev. 1.2.:<br>• Agriculture – section A , division -01<br>• Forestry – section A, division 02	(Śleszyński 2009)	2809	No data on employment in certain sectors at the local level

	Forestry -- (Niedziałkowski et al. 2012b, Logmani et al. 2017, Olko et al. 2011, Niedziałkowski et al. 2014, Bielecka and Różyński 2014)<br><br>Timber market -- (Olko et al. 2011, Niedziałkowski et al. 2012b)<br><br>Transport -- (Bielecka and Różyński 2014, Piwowarczyk and Wróbel 2016)<br><br>Industry -- (Kubacka and Macias 2016, Niedziałkowski et al. 2012b)<br><br>Energy -- (Bernat 2016)		• Fishing – Section A, division 03<br>• Industry – section B-F<br>• Tourism – section I<br>• Transport – section H<br>• Energy – section D			
Employment in certain sectors / engagement in certain activities	Public administration -- (Grodzinska-Jurczak and Cent 2011, Olko et al. 2011, Piwowarczyk and Wróbel 2016, Chmielewski and Głogowska 2015, Niedziałkowski et al. 2014, Paloniemi et al. 2015, Niedziałkowski et al. 2012a)<br><br>Nature conservation -- (Olko et al. 2011, Chmielewski and Głogowska 2015, Niedziałkowski et al. 2012b, Niedziałkowski et al. 2014)<br><br>Agriculture -- (Olko et al. 2011, Grodzinska-Jurczak and Cent 2011, Niedziałkowski et al. 2012a, Jankowiak et al. 2015)<br><br>Forestry -- (Olko et al. 2011, Niedziałkowski et al. 2012b, Maczka et al. 2019, Chmielewski and Głogowska 2015, Niedziałkowski et al. 2014, Paloniemi et al. 2015)<br><br>Timber industry -- (Niedziałkowski et al. 2014)	34.<br>35.<br>36.<br>38.<br>40.<br>41.<br>42.<br>43.<br>44.<br>45.<br>46.<br>47.<br>48.<br>49.	A share of entered entities into certain sections and divisions of the NACE rev. 1.2 (PKD 2007) in a total number of the entities entered to the NACE rev. 1.2.:<br>• Agriculture and hunting – section A, division -01<br>• Forestry – section A, division 02<br>• Fishing – Section A, division 03<br>• Public administration – Section O<br>• Constructions – section F<br>• Timber Industry – section C, division 16<br>• Tourism – section I<br>• Transportation – section H<br>• Real estate – section L<br>• NGOs – section S, division 94<br>• Science – section M, division 72<br>• Media industry – section J		2809	No data on local media available<br><br>No data on employment in certain sections, only proxy data on number of entities

	Fishing -- (Piwowarczyk and Wróbel 2016, Maczka et al. 2019, Wiatkowski et al. 2017, Bielecka and Różyński 2014)<br><br>Tourism industry -- (Olko et al. 2011, Bernat 2016, Bielecka and Różyński 2014)<br><br>Real estate, development -- (Grodzinska-Jurczak and Cent 2011, Bielecka and Różyński 2014, Niedziałkowski et al. 2012a)<br><br>Hunting -- (Olko et al. 2011)<br><br>Construction -- (Niedziałkowski et al. 2012a)<br><br>NGOs -- (Niedziałkowski et al. 2012a, Maczka et al. 2019, Chmielewski and Głogowska 2015, Paloniemi et al. 2015, Niedziałkowski et al. 2012b, Niedziałkowski et al. 2014, Piwowarczyk and Wróbel 2016, Logmani et al. 2017)<br><br>Science -- (Piwowarczyk and Wróbel 2016, Paloniemi et al. 2015, Niedziałkowski et al. 2012b, Niedziałkowski et al. 2014, Niedziałkowski et al. 2012a)<br><br>Transport -- (Bernat 2016)<br><br>Media industry -- (Niedziałkowski et al. 2012a)<br><br>Education -- (Bielecka and Różyński 2014)		• Education – section P<br>• Nature conservation – section R, division 91			
		33.	foundations, associations and social organizations per 10 thous. population		2419	
Economic role of SHF (employment, revenues)	(Niedziałkowski et al. 2014, Niedziałkowski et al. 2012b, Olko et al. 2011)	50.	Share of entered public sector entities in the section A of the NACE rev. 1.2 in a total number of national economy entities in gmina		2809	Proxy data on public sector entities only.
Private sector	(Piwowarczyk and Wróbel 2016, Maczka et al. 2019)	53.	Share of private sector entities in a total number of entities of		2822	

			the national economy in a gmina (only mean data)			
Investments (individual and private-sector)	(Chmielewski and Głogowska 2015)<br><br>In coastal infrastructure -- (Bielecka and Różyński 2014, Piwowarczyk and Wróbel 2016)<br><br>In renewable energy (wind farms, biogas plants), in shell gas prospecting -- (Chmielewski and Głogowska 2015, Bernat 2016)<br><br>In fish farming -- (Wiatkowski et al. 2017)<br><br>Housing and recreational -- (Wilkaniec et al. 2020, Kubacka 2012, Zawilińska 2020)<br><br>Building, reconstructing the house -- (Olko et al. 2011, Grodzinska-Jurczak and Cent 2011, Zawilińska 2020)	54.	New one-dwelling residential buildings not adapted to permanent residence per 1000 population		(3812/2914) * 1000 inh	No data on average plot size of the investments<br><br>No data on specific types of private investments<br><br>No data on reconstructions at the local level
		58.	New residential buildings per 1000 population		3820	
		114.	total area of forest land, which were designated in local spatial development plans for non-forest purposes	(Śleszyński et al. 2018)	2847/1410	
		115.	total area of agricultural land, which were designated in local spatial development plans for non-agricultural purposes	(Śleszyński et al. 2018)	2847/1410	
		118.<br>119.	Land development decisions total per 1000 inhabitants:<br>• Single-family housing<br>• Multi-family housing + service buildings<br>Other buildings	(Hajduk 2018, Śleszyński et al. 2018)	(2851/2914)* 1000 inh	
		120.	Requiring to change designation of agricultural land for non-agricultural purposes in Study of conditions and directions of spatial management	(Śleszyński et al. 2018)	3178/1410	
No. of car parks	(Piwowarczyk and Wróbel 2016)	60.	No of park and ride car parks per 1000 inh (mean data only)		(3602/2914)* 1000 inh	
Agrotourism development, employment in agrotourism, agrotourism investments	(Grodzinska-Jurczak and Cent 2011, Olko et al. 2011)	67.	No of agrotourism lodgings per 100 km2	(Pałka 2007)	(2017/1410)* 100	No data on the structure of PROW grants at the local level
		68.	No of bed places in agricultural lodgings per 1000 inhabitants in gmina	(Zawadka 2013, Męczekalski, Dubownik and Rudnicki 2017)	(2017/2914)* 1000 inh	

		69.	Share of all-year opened agricultural lodgings in a total of agricultural lodgings	(Zawadka 2013, Męczekalski et al. 2017)	2017	No data on the number of (domestic or foreign) tourists in agrotourism lodgings<br><br>No data on nights spent in agrotourism lodgings<br><br>No data on the number of agricultural farms for a time section for which data on agrotourism lodgings are available
Tourism infrastructure development	(Grodzinska-Jurczak and Cent 2011, Bielecka and Różyński 2014, Zawilińska 2020)<br><br>Big hotels, ski lifts, infrastructure for massive tourism, infrastructure for hiking tourism (shelters), trails -- (Olko et al. 2011, Brown et al. 2015, Zawilińska 2020)<br><br>Marinas, hotels, beach bars, restaurants -- (Piwowarczyk and Wróbel 2016, Zawilińska 2020)<br><br>Recreational facilities -- (Wasilewski, Szulczewska and Giedych 2019)	64.	Baretje-Defert's index – Total number of bed places per 100 inhabitatns	(Warczewska 2017)	(2017/2914)* 100 inh	No data on marinas and ski lifts at the local level<br><br>Only proxy data available for bars and restaurants – limited to units in tourist establishments<br><br>Recreational facilities reduced to outdoor gyms (based on the context in the source paper)
		70.<br>71.	Tourist accommodation establishments per 10 th population:<br>• Hotels<br>• Shelters	(Warczewska 2017)	(2017/2914)* 10 000 inh	
		74.<br>75.	Catering units in tourist accomodation establishments:<br>• Restaurants<br>• Bars and cafeterias<br>per 10 th population		(3607/2914)* 10 000 inh	
		84.	Outdoor gyms per 10 th inhabitatns		3881	
Structure of tourism	Leisure, recreational tourism -- (Kubacka 2012, Bielecka and Różyński 2014, Bernat 2016, Piwowarczyk and Wróbel 2016, Niedziałkowski et al. 2014, Wilkaniec et al. 2020, Wasilewski et al. 2019, Brown et al. 2015, Zawilińska 2020)<br><br>One-day tourism -- (Bielecka and Różyński 2014)<br><br>International tourism -- (Bielecka and Różyński 2014)	72.	Share of number of bed places open all year in a total number of bed places in a gmina	(Miedziński 2015)	2017	Proxy data only for recreational tourism (share of recreational lands in a total gmina land)<br><br>No precise data on structure of tourism
		73.	Share of international tourists in a total number of accomodated tourists in gmina		2017	
		126.	Share of recreational areas		2779	

	Water tourism -- (Bielecka and Różyński 2014, Piwowarczyk and Wróbel 2016)<br><br>Summer tourism -- (Bielecka and Różyński 2014, Borkowski et al. 2019)					
Intensity of agricultural production	(Niedziałkowski et al. 2012a, Jankowiak et al. 2015, Wilkaniec et al. 2020)	150.	Hectares of Agricultural Land per 1 tractor	(Lorencowicz, Mazurek and Kocira 2017)	3020/3024	No data on mechanical pulling force are available at the local level<br><br>No data on crops from 1 ha are available at the local level<br><br>No data on milk or egg production available at the local level<br><br>No trend data on:<br>• size of agricultural holdings available at the local level<br>• in fallow areas<br>• hectares per tractor<br>• livestock per agricultural land<br>• consumption of mineral fertilisers<br>• Structure of sawn areas<br>The data above refer to agricultural holdings based in the gmina (with their lands possibly located outside)
		151.<br>152.<br>153.	Livestock per 100 ha of agricultural lands:<br>• Cattle<br>• Pigs<br>• Poultry	(Lorencowicz et al. 2017)	(3028/3020)* 100 ha	
		158.	Consumption of mineral fertilizers per 1 ha of agricultural land	(Lorencowicz et al. 2017)	3182	
		142.<br>143.	Share of agricultural holdings:<br>• 10 ha and more<br>• Up to 1 ha	(Lorencowicz et al. 2017)	3019	
		147.<br>163.<br>164.<br>165.<br>166.<br>167.<br>168.<br>169.<br>170.	Sown area per agricultural holding types:<br>• Cereals<br>• Maize for grain<br>• Potatoes<br>• Industrial crops<br>• Sugar beets<br>• Rapeseed<br>• Edible pulses for grain<br>• Field vegetables<br>• Fallows areas	(Lorencowicz et al. 2017)	3416<br>3020	
Tourism intensity (no of tourists)	(Bernat 2016, Logmani et al. 2017, Borkowski et al. 2019, Bielecka and Różyński 2014)	63.	Schneider's index - Total number of accommodated tourists per 100 inhabitants	(Gonia and Podgórski 2019)	(2017/2914)* 100 inh	
		66.	Chavart index - Total number of nights spent in tourist accommodation / 100 inhabitatns	(Gonia and Podgórski 2019)	(2017/2914)* 100 inh	

Level of social capital	(Niedziałkowski et al. 2012b)	33.	foundations, associations and social organizations per 10 thous. population	(Działek 2011, Dominiak and Konecka-Szydłowska 2020, Wojewódzka-Wiewiórska 2014)	2419	
		76.	Members of sports clubs per 10 000 inhabitants	(Działek 2011)	(2155/2914)* 10000 inh	
		77.	No of events organized by centres of culture, cultural centres and establishments, clubs and community centres per 10 000 inhabitants	(Dominiak and Konecka-Szydłowska 2020)	(2284/2914)* 10000 inh	
		81.	members of artistic groups per 10 000 inhabitants	(Działek 2011, Dominiak and Konecka-Szydłowska 2020)	(2792/2914)* 10000 inh	
		82.	members of groups (clubs/sections) per 10 000 inhabitatns	(Działek 2011, Dominiak and Konecka-Szydłowska 2020)	(2793/2914)* 10000 inh	
		83.	groups (clubs/sections) per 10 000 inhabitnts	(Dominiak and Konecka-Szydłowska 2020)	(2793/2914)* 10000 inh	
Population density	(Niedziałkowski et al. 2014, Kubacka and Macias 2016, Bielecka and Różyński 2014)	86.	population per 1 km2 (<u>mean data only</u>)		2425	
Urbanization rate (urban/rural residents)	(Grodzinska-Jurczak and Cent 2011, Wasilewski et al. 2019, Bielecka and Różyński 2014, Strzelecka et al. 2020)	88.	Urban population in % of total population		2463	
Total population	(Grodzinska-Jurczak and Cent 2011, Olko et al. 2011, Niedziałkowski et al. 2014, Bielecka and Różyński 2014, Kubacka and Macias 2016, Wiatkowski et al. 2017)	89.	Total population (<u>mean data only</u>)		2914	
Gender structure	(Niedziałkowski et al. 2014, Paloniemi et al. 2015, Brown et al. 2015)	93.	Feminity ratio (<u>mean data only</u>)		3429	

Age structure	(Niedziałkowski et al. 2014, Wasilewski et al. 2019, Brown et al. 2015, Zawilińska 2020)	94.<br>95.<br>96.<br>97.	Share of population in the group sections:<br>• 19-24<br>• 25-44<br>• 45-64<br>• 65+		3447	
Number of people living on the social security	(Olko et al. 2011)	102.	beneficiaries of social assistance per 10 thous. of population		3870	
Unemployment rate	(Grodzinska-Jurczak and Cent 2011, Olko et al. 2011, Niedziałkowski et al. 2014, Bielecka and Różyński 2014, Piwowarczyk and Wróbel 2016)	103.	Share of the registered unemployed persons in the population in the working age		2670	
Representation of foresters in a municipal board	(Niedziałkowski et al. 2012b)	112.	Share of skilled agricultural, forestry and fishery workers in a total number of gmina councillors		1314	
Level of education	(Niedziałkowski et al. 2012b, Piwowarczyk and Wróbel 2016, Wasilewski et al. 2019)<br><br>Vs. local elites -- (Niedziałkowski et al. 2012b)	113.	Share of gmina councillors with higher education		1316	Only proxy data on the level of education of gmina councillors available at the local level
Planning permissions	For residential buildings -- (Grodzinska-Jurczak and Cent 2011, Zawilińska 2020)	55.	permits and registrations with project for residential buildings per 1000 population		(3818/2914)* 1000 inh	
	For non-residential building -- (Bielecka and Różyński 2014)	56.<br>57.	Permits granted for construction of non-residential and civil engeneering works:<br>- Hotels and similar buildings<br>- Civil engeneering workds<br>Per 1000 inhabitants		(3819/2914)* 1000 inh	

Land ownership	State forests -- (Olko et al. 2011, Niedziałkowski et al. 2014, Borkowski et al. 2019)<br><br>Private forests -- (Grodzinska-Jurczak and Cent 2011, Niedziałkowski et al. 2014)<br><br>Nationalisation of private lands for PA establishment -- (Strzelecka et al. 2020, Grodzinska-Jurczak and Cent 2011, Niedziałkowski et al. 2014, Brown et al. 2015)<br><br>Private lands in PAs -- (Grodzinska-Jurczak and Cent 2011, Paloniemi et al. 2015, Kubacka and Macias 2016, Strzelecka et al. 2020):<br>- In the buffer zone of a PA -- (Olko et al. 2011)<br><br>State lands in PAs -- (Olko et al. 2011, Piwowarczyk and Wróbel 2016)<br><br>Private farms -- (Grodzinska-Jurczak and Cent 2011, Borkowski et al. 2019, Strzelecka et al. 2020)<br>- Meadows -- (Grodzinska-Jurczak and Cent 2011)<br><br>Private developed lands -- (Grodzinska-Jurczak and Cent 2011, Olko et al. 2011)<br><br>Private lands designated for development -- (Grodzinska-Jurczak and Cent 2011, Wilkaniec et al. 2020, Niedziałkowski et al. 2014)<br><br>Road ownership -- (Olko et al. 2011)	109.<br>110.<br>111.	Share of:<br>• Public forests owned by State Treasury managed by State Forests<br>• Public forests owned by State Treasury – National Parks<br>• Private forests<br>In a total gmina surface area		1408/1410	No data on structure of land properties within PAs are available at the local level – only proxy data on public forests in NPs<br><br>No data on the structure of lands outside of forests – only proxy data for farmlands<br><br>Data on agricultural lands of private farms refer to base of agricultural holdings based in a gmina (with their lands located possibly outside). Longitudinal data are not available at the local level.
		145.<br>146.	Share of agricultural lands of private farms in a total gmina surface area<br>• Share of meadows in private farms in a total gmina surface area		3020/1410	

Land designated for development in physical development plans	(Grodzinska-Jurczak and Cent 2011)<br><br>In study of conditions and directions of spatial development -- (Wilkaniec et al. 2020)	115.	total area of agricultural land, which were designated in plans for non-agricultural purposes		2847/1410	Only proxy data available at the local level
		114.	total area of forest land, which were designated in plans for non-forest purposes		2847/1410	
		120.	requiring to change designation of agricultural land for non-agricultural purposes		3178/1410	
		121.	requiring to change designation from forest land for non-forest purposes		3178/1410	
Municipal physical development plans	(Grodzinska-Jurczak and Cent 2011, Olko et al. 2011, Bielecka and Różyński 2014, Chmielewski and Głogowska 2015, Zawilińska 2020)	116.	share of the area covered by valid local spatial development plans in total area		2847	
Land fragmentation	(Grodzinska-Jurczak and Cent 2011, Niedziałkowski et al. 2012a, Wasilewski et al. 2019)	140.	The average area of total agricultural land	(Sroka 2018)	3016	No data on economic power of agricultural holdings at the local level<br><br>No data on the number of plots and their average area within an agricultural holding at the local level<br><br>Data on average area of agricultural land are imprecise as it refers to agricultural holdings based in the gmina (with their lands possibly located outside). No longitudinal data available.
		154.<br>155.<br>156.<br>157.	Area of farms in agricultural holdings:<br>• Up to 1 ha<br>• 1-5 ha<br>• 5-10 ha<br>• 10 ha or more	(Sroka 2018, Janus and Taszakowski 2013)	3030	
Wood/timber production, logging	In private forests --- (Grodzinska-Jurczak and Cent 2011)	122.	Removals (timber) in private forests per 1 km2		1483/1410	Data only for private forests

	In state forests -- (Olko et al. 2011, Niedziałkowski et al. 2012b, Logmani et al. 2017, Niedziałkowski et al. 2014, Chmielewski and Głogowska 2015)					
Transport infrastructure development	(Piwowarczyk and Wróbel 2016)<br><br>Road infrastructure/network -- (Grodzinska-Jurczak and Cent 2011, Niedziałkowski et al. 2012a, Chmielewski and Głogowska 2015, Jankowiak et al. 2015, Bernat 2016)<br><br>*Network of airports* -- (Bernat 2016)	127.	Share of road areas		2779	No data on road density at the local level – only proxy data available (share of road areas in a total area of a municipality)<br><br>No data on airports at the local level
		128.	Share of other transport areas		2779	

Land use and its change	(Grodzinska-Jurczak and Cent 2011, Olko et al. 2011, Niedziałkowski et al. 2012b, Kubacka and Macias 2016, Maczka et al. 2019, Wilkaniec et al. 2020)<br><br>Agricultural lands -- (Niedziałkowski et al. 2012a, Niedziałkowski et al. 2014, Kubacka and Macias 2016, Maczka et al. 2019, Borkowski et al. 2019, Strzelecka et al. 2020, Jankowiak et al. 2015)<br>- Meadows -- (Borkowski et al. 2019, Wilkaniec et al. 2020)<br>- Arable lands -- (Borkowski et al. 2019, Wilkaniec et al. 2020, Grodzinska-Jurczak and Cent 2011, Kubacka and Macias 2016, Jankowiak et al. 2015)<br>- Permanent crops -- (Wilkaniec et al. 2020)<br>- Pastures - (Jankowiak et al. 2015, Wilkaniec et al. 2020)<br><br>Forests -- (Niedziałkowski et al. 2014, Kubacka and Macias 2016, Borkowski et al. 2019, Grodzinska-Jurczak and Cent 2011, Olko et al. 2011, Niedziałkowski et al. 2012b, Bielecka and Różyński 2014, Logmani et al. 2017, Maczka et al. 2019, Wasilewski et al. 2019, Wilkaniec et al. 2020)<br><br>Urbanized areas -- (Kubacka and Macias 2016, Jankowiak et al. 2015)<br>- Building developments -- (Kubacka 2012, Grodzinska-Jurczak and Cent 2011, Piwowarczyk and Wróbel 2016, Maczka et al. 2019, Wilkaniec et al. 2020)<br>- Recreational areas (Wilkaniec et al. 2020, Wasilewski et al. 2019)<br>- Industrial areas -- (Wilkaniec et al. 2020)	125.<br>126.<br>127.<br>129.<br>130.<br>131.<br>132.<br>133.<br>134.<br>135.<br>136.<br>137.<br>138.<br>148.	Share of:<br>• Agricultural lands<br>• Arable lands<br>• Meadows<br>• Pasutures<br>• Permanent crops<br>• Forests<br>• Urbanized areas<br>• Built-up areas<br>• Industrial areas<br>• Minerals<br>• Recreational areas<br>• Road areas<br>• Flowing waters<br>• Standing waters<br>• Wastelands		2779<br>3020/2779	No data available on wetlands<br><br>Longitudinal data is not available for permanent crops. The data refer to agricultural holdings based in the gmina (possibly located outside)

	- Minerals -- (Kot-Niewiadomska and Pawłowska 2020)<br>- Roads -- (Wilkaniec et al. 2020, Grodzinska-Jurczak and Cent 2011, Niedziałkowski et al. 2012a, Jankowiak et al. 2015, Bernat 2016)<br><br>Water bodies -- (Kubacka and Macias 2016, Borkowski et al. 2019, Maczka et al. 2019, Bielecka and Różyński 2014, Bernat 2016, Wiatkowski et al. 2017)<br><br>Flowing water -- (Kubacka and Macias 2016, Wiatkowski et al. 2017)<br><br>Wetlands -- (Wiatkowski et al. 2017, Borkowski et al. 2019, Wilkaniec et al. 2020, Jankowiak et al. 2015)<br><br>Wastelands -- (Wilkaniec et al. 2020)					

Urbanization pressure	(Brown et al. 2015, Wasilewski et al. 2019, Wilkaniec et al. 2020, Zawilińska 2020)	139.	PA areas per urbanized areas in a gmina	(Chmielewski et al. 2018)	1659/2779	
Use of fertilizers in agriculture	(Bielecka and Różyński 2014)	159.<br>160.	Use of nitrogenous and phosphatic fertilizers in agricultural holdings per 100 ha of a gmina		(3182/1410)* 100 ha	Longitudinal data are not available at the local level. The data refers to agricultural holdings based in a gmina (with their lands possibly located outside)
Structure of crops	(Jankowiak et al. 2015, Borkowski et al. 2019)	163.<br>164.<br>168.	Sown area per agricultural holding types:<br>• Cereals<br>• Maize for grain<br>• Rapeseed		3416	
Biodiversity loss	(Niedziałkowski et al. 2014, Paloniemi et al. 2015, Brown et al. 2015, Borkowski et al. 2019, Wilkaniec et al. 2020, Niedziałkowski et al. 2012b, Strzelecka et al. 2020)	171.	Share of PAs in a total gmina surface	(Śmietanka 2018)	(1659/1410)* 100	Only proxy data are available<br><br>Data on area of farms in agricultural holdings not included due to errors in the dataset
		186.	Share of green areas in a total gmina surface	(Śmietanka 2018)	(2596/1410)* 100	
Protected areas in a municipality	(Grodzinska-Jurczak and Cent 2011, Niedziałkowski et al. 2012b, Niedziałkowski et al. 2012a, Niedziałkowski et al. 2014, Chmielewski and Głogowska 2015, Brown et al. 2015, Kubacka and Macias 2016, Bernat 2016, Piwowarczyk and Wróbel 2016, Logmani et al. 2017, Wiatkowski et al. 2017, Wasilewski et al. 2019, Borkowski et al. 2019, Wilkaniec et al. 2020, Strzelecka et al. 2020)<br><br>- national parks (Grodzinska-Jurczak and Cent 2011, Olko et al. 2011, Niedziałkowski et al. 2012a, Niedziałkowski et al. 2012b, Niedziałkowski et al. 2014, Brown et al. 2015, Bernat 2016, Logmani et al. 2017, Borkowski et al. 2019, Strzelecka et al. 2020, Zawilińska 2020)<br>- nature reserves (Niedziałkowski et al. 2012a, Niedziałkowski et al. 2012b, Bernat 2016, Wasilewski et al. 2019)	171.<br>172.<br>173.<br>174.<br>175.	Share of PAs in a total gmina surface:<br>- NPs<br>- LPs<br>- PLAs<br>- Nature reserves		(1659/1410)* 100	No data on N2000 are available at the local level<br><br>No data on PA buffer zones available

	- landscape parks -- (Niedziałkowski et al. 2012a, Bielecka and Różyński 2014, Wilkaniec et al. 2020, Strzelecka et al. 2020, Kubacka and Macias 2016, Kot-Niewiadomska and Pawłowska 2020)<br>- Natura 2000 -- (Grodzinska-Jurczak and Cent 2011, Niedziałkowski et al. 2014, Bielecka and Różyński 2014, Chmielewski and Głogowska 2015, Jankowiak et al. 2015, Paloniemi et al. 2015, Brown et al. 2015, Kubacka and Macias 2016, Piwowarczyk and Wróbel 2016, Logmani et al. 2017, Wiatkowski et al. 2017, Maczka et al. 2019, Wilkaniec et al. 2020, Strzelecka et al. 2020)<br>- Areas of protected landscape (Bernat 2016, Wilkaniec et al. 2020)<br>- NP buffer zone -- (Olko et al. 2011, Bernat 2016, Zawilińska 2020)<br>- LP buffer zone -- (Wilkaniec et al. 2020)					
Water treatment plants	(Wiatkowski et al. 2017, Bielecka and Różyński 2014)	176.<br>177.	Water treatment plants with increased biogene removals:<br>• Capacity per 1000 population<br>• Share of population connected		(1670/2914)* 1000 inh	
Number of boats	(Bielecka and Różyński 2014, Bernat 2016)<br><br>Harbour fees -- (Bielecka and Różyński 2014)					Data available only for marine transport, which is not relevant to the studied gminas

[1] The colour indicates the assignment of a variable to a group of PA conflict determinants in the data classification phase. Blue – economic, red – socio-cultural, violet – institutional, green – environmental

[2] The colour indicates additional assignment(s) of a variable to a group of PA conflict determinants at the phase of data typology (description of colours – the same as above).

Tab. S3.2. Factors of PA conflicts not represented by local-level variables in the Local Data Bank of Statistics Poland (some of them are available only for higher levels of spatio-jurisdictional scale)

Group of det.	PA conflict factor	Source for the factor choice	Comments
Soc	Ethnic structure	(Niedziałkowski et al. 2012b)	No data on ethnic structure of the population is available at the local level
Inst	Municipal strategic plans	(Grodzinska-Jurczak and Cent 2011, Chmielewski and Głogowska 2015)	No data on strategic development plans are available in the database
Soc/ Inst/ Econ	Public transport	(Bielecka and Różyński 2014, Piwowarczyk and Wróbel 2016)	No data on:<br>• number of passengers of public transport<br>• number of cars per 1000 inhabitants<br>• average number of public transport trips<br><br>are available at the local level<br><br>Data on passengers in marine transport are available but were not used as they are not applicable to the case study region.
Econ	Rise in prices/supply of /demand for certain assets	Wood - (Niedziałkowski et al. 2014)<br>Properties - (Grodzinska-Jurczak and Cent 2011, Bernat 2016, Kubacka 2012)<br>Tourist facilities (Piwowarczyk and Wróbel 2016, Bielecka and Różyński 2014)	Data on prices are not available at the local level
Econ	(sources of) Individual income (e.g., tourism)	(Niedziałkowski et al. 2014, Bielecka and Różyński 2014, Kubacka and Macias 2016, Maczka et al. 2019)	No data on individual income is available at the local level
Econ	Road accessibility, road quality	(Bielecka and Różyński 2014)	Data on road quality or road accessibility are not available at the local level
Econ	Road traffic	(Olko et al. 2011, Niedziałkowski et al. 2012a, Bernat 2016)	Data on road traffic are not available at the local level
Econ	Harbour fees	(Bielecka and Różyński 2014)	No relevant data available
Econ	Food production	(Jankowiak et al. 2015, Wilkaniec et al. 2020)	No data on food production available at the local level
Econ/ Soc	Structure of fuels used in households	(Niedziałkowski et al. 2014)	There are no data on the structure of fuels in the households used available at the local level
Soc/ Env	Hunted animals	(Olko et al. 2011, Borkowski et al. 2019)	No hunting data are available at the local level
Soc/ Econ/ Env	Noise pollution	(Bernat 2016)	Data on noise levels are not available
Inst/ Econ/ Env	Agri-environmental schemes	(Strzelecka et al. 2020)	Data on agri-environmental schemes are not available at the local level
Econ/ Env	Environmental Impact assessments	(Niedziałkowski et al. 2012a, Bielecka and Różyński 2014, Bernat 2016, Kot-Niewiadomska and Pawłowska 2020)	Data on the number of EIAs are not available in the database

Econ/ Env	Fishing intensity	(Bielecka and Różyński 2014, Piwowarczyk and Wróbel 2016, Maczka et al. 2019)	No relevant data available at the local level
Env	Age of tree stands	(Niedziałkowski et al. 2012b, Paloniemi et al. 2015)	Data on age of tree stands are not available at the local level
Env	Species structure of tree stands	(Niedziałkowski et al. 2012b, Paloniemi et al. 2015, Borkowski et al. 2019)	Data on tree stands are not available at the local level
Env	Invasive species	(Wiatkowski et al. 2017)	Data on invasive species are not available
Env	Groundwater level	(Wiatkowski et al. 2017, Borkowski et al. 2019)	Data on groundwater level are not available
Env	Endangered /protected habitats/species	(Olko et al. 2011, Niedziałkowski et al. 2012b, Niedziałkowski et al. 2014, Bielecka and Różyński 2014, Grodzinska-Jurczak and Cent 2011, Kubacka and Macias 2016, Piwowarczyk and Wróbel 2016, Logmani et al. 2017, Wiatkowski et al. 2017, Maczka et al. 2019, Borkowski et al. 2019, Strzelecka et al. 2020)	Data on wild species are not available at the local level
Env/ Econ	Animal damages in agriculture	(Chmielewski and Głogowska 2015, Jankowiak et al. 2015, Maczka et al. 2019, Borkowski et al. 2019, Olko et al. 2011, Niedziałkowski et al. 2012b, Niedziałkowski et al. 2014, Zawilińska 2020)<br><br>Refunds for the damages -- (Olko et al. 2011)	No relevant data available
Env	Emission of air pollutants from plants	(Kot-Niewiadomska and Pawłowska 2020)	No data available at the local level

Tab. S.3.3. A complete list of the variables used in the study. For an explanation of the shortcuts, please refer to tab S3.1.

No.	Code name of a variable (no, mean or trend, time section, shortcut decription)	Rationale for exclusion of varaibles prior to actual analyses (if applicable)[1]
1	1_2007_20_own_revenue_share	
2	1sl_2007_20_own_revenue_share	
3	2_2007_20_agric_tax_share	
4	2sl_2007_20_agric_tax_share	
5	3_ 2007_20_agric_tax_capita	Correlation coefficient over 0,9 with var. 2
6	3sl_2007_20_agric_tax_capita	
7	4_2007_20_grants_subv_share	
8	4sl_2007_20_grants_subv_share	
9	5_2007_20_gov_grants_capita	
10	5sl_2007_20_gov_grants_capita	
11	6_2007_20_own_revenue_capita	Correlation coefficient over 0,9 with var. 1
12	6sl_2007_20_own_revenue_capita	
13	7_2007_20_revenue_capita	
14	7sl_2007_20_revenue_capita	Correlation coefficient over 0,9 with var. 10sl
15	8_2007_20_invest_exp	
16	8sl_2007_20_invest_exp	
17	9_2007_20_road_exp	

18	9sl_2007_20_road_exp	
19	10_2007_20_exp_capita	
20	10sl_2007_20_exp_capita	
21	11_2008_20_debt_capita	
22	11sl_2008_20_debt_capita	Correlation coefficient below -0,9 with var. 11
23	12_2008_20_health_exp	
24	12sl_2008_20_health_exp	
25	13_2008_20_env_exp	
26	13sl_2008_20_env_exp	
27	14_2008_20_transport_exp	
28	14sl_2008_20_transport_exp	
29	15_2008_20_nature_exp	
30	15sl_2008_20_nature_exp	
31	16_2017_20_env_UE_grants_app	
32	16sl_2017_20_env_UE_grants_app	
33	17_2016_20_infr_UE_funds	
34	17sl_2016_20_infr_UE_funds	
35	18_2016_20_social_UE_funds	
36	18sl_2016_20_social_UE_funds	Correlation coefficient over 0,9 with var. 18 (only normalized variables)
37	19_2007_15_dwellings_with_debts	
38	19sl_2007_15_dwellings_with_debts	
39	20_2007_20_dwelling_all	
40	20sl_2007_20_dwelling_all	
41	21_2007_20_floor_area	
42	21sl_2007_20_floor_area	
43	22_2007_20_dwellings_capita	
44	22sl_2007_20_dwellings_capita	
45	23_2007_19_dwellings_water	
46	23sl_2007_19_dwellings_water	
47	24_2007_19_dwellings_fl_lavatory	Correlation coefficient over 0,9 with var. 25
48	24sl_2007_19_dwellings_fl_lavatory	
49	25_2007_19_dwellings_bathroom	
50	25sl_2007_19_dwellings_bathroom	
51	26_2007_19_dwellings_centr_heat	Correlation coefficient over 0,9 with var. 25
52	26sl_2007_19_dwellings_centr_heat	
53	27_2007_19_dwellings_gas	
54	27sl_2007_19_dwellings_gas	
55	28_2007_20_water_system	
56	28sl_2007_20_water_system	
57	29_2007_20_sewage_system	
58	29sl_2007_20_sewage_system	
59	30_2007_20_gas_system	
60	30sl_2007_20_gas_system	

61	31_2007_20_econ_activ	Correlation coefficient over 0,9 with var. 32
62	31sl_2007_20_econ_activ	
63	32_2007_20_REGON	
64	32sl_2007_20_REGON	Correlation coefficient over 0,9 with var. 31sl
65	33_2007_20_foundations	
66	33sl_2007_20_foundations	
67	34_2009_20_REGON_agric	
68	34sl_2009_20_REGON_agric	
69	35_2009_20_REGON_forest	
70	35sl_2009_20_REGON_forest	
71	36_2009_20_REGON_fish	
72	36sl_2009_20_REGON_fish	
73	37_2009_20_REGON_industry_min	
74	37sl_2009_20_REGON_industry_min	
75	38_2009_20_REGON_timber_indust	
76	38sl_2009_20_REGON_timber_indust	
77	39_2009_20_REGON_energy	
78	39sl_2009_20_REGON_energy	
79	40_2009_20_REGON_construct	
80	40sl_2009_20_REGON_construct	
81	41_2009_20_REGON_transport	
82	41sl_2009_20_REGON_transport	
83	42_2009_20_REGON_tourism	
84	42sl_2009_20_REGON_tourism	
85	43_2009_20_REGON_media	
86	43sl_2009_20_REGON_media	
87	44_2009_20_REGON_real_estate	
88	44sl_2009_20_REGON_real_estate	
89	45_2009_20_REGON_science	
90	45sl_2009_20_REGON_science	
91	46_2009_20_REGON_admin	
92	46sl_2009_20_REGON_admin	
93	47_2009_20_REGON_educ	
94	47sl_2009_20_REGON_educ	
95	48_2009_20_REGON_cult_nature	
96	48sl_2009_20_REGON_cult_nature	
97	49_2009_20_REGON_org	
98	49sl_2009_20_REGON_org	
99	50_2009_20_public_A	
100	50sl_2009_20_public_A	
101	51_2007_20_commerce_domest	
102	51sl_2007_20_commerce_domest	
103	52_2007_20_commerce_foreign	
104	52sl_2007_20_commerce_foreign	
105	53_2007_20_private_sect	

106	54_2007_20_new_temp_resident	
107	54sl_2007_20_new_temp_resident	
108	55_2018_20_construction_permits_resid	
109	55sl_2018_20_construction_permits_resid	
110	56_2018_20_construction_permits_hotel	
111	56sl_2018_20_construction_permits_hotel	
112	57_2018_20_construction_permits_engin	
113	57sl_2018_20_construction_permits_engin	
114	58_2007_20_new_perm_resident	
115	58sl_2007_20_new_perm_resident	
116	59_2007_20_new_dwelings	
117	59sl_2007_20_new_dwelings	
118	60_2016_20_park_and_ride	
119	61_2007_20_guesthouse	
120	62_2007_14_tourists_km2	
121	62sl_2007_14_tourists_km2	
122	63_2007_14_tourists_capita	
123	63sl_2007_14_tourists_capita	
124	64_2007_20_bed_places_capita	
125	64sl_2007_20_bed_places_capita	
126	65_2007_20_bed_places_km2	Correlation coefficient over 0,9 with var. 62
127	65sl_2007_20_bed_places_km2	
128	66_2007_14_nights_capita	Correlation coefficient over 0,9 with var. 63
129	66sl_2007_14_nights_capita	
130	67_2012_20_agritourism_lodg	Correlation coefficient over 0,9 with var. 68
131	67sl_2012_20_agritourism_lodg	
132	68_2012_20_agritourism_beds	
133	68sl_2012_20_agritourism_beds	
134	69_2012_20_agritourism_all_year	
135	69sl_2012_20_agritourism_all_year	
136	70_2007_20_hotels	
137	70sl_2007_20_hotels	
138	71_2007_20_shelters	
139	71sl_2007_20_shelters	
140	72_2007_20_all_year_bed_places	
141	72sl_2007_20_all_year_bed_places	
142	73_2007_14_intern_tourism	
143	73sl_2007_14_intern_tourism	
144	74_2018_20_restaurants	
145	74sl_2018_20_restaurants	
146	75_2018_20_bars	Correlation coefficient over 0,9 with var. 74
147	75sl_2018_20_bars	
148	76_2008_20_sport_memb	
149	76sl_2008_20_sport_memb	

150	77_2007_20_events	
151	77sl_2007_20_events	
152	78_2007_20_libraries	
153	78sl_2007_20_libraries	
154	79_2007_20_library_borrowers	
155	79sl_2007_20_libraries_borrowers	
156	80_2007_20_library_loans	
157	80sl_2007_20_library_loans	
158	81_2007_20_artist_memb	
159	81sl_2007_20_artist_memb	
160	82_2007_20_group_memb	
161	82sl_2007_20_group_memb	Correlation coefficient over 0,9 with var. 82 (only normalized variabes)
162	83_2007_20_groups	Correlation coefficient over 0,9 with var. 82
163	83sl_2007_20_groups	
164	84_2014_18_outdoor_gym	
165	84sl_2014_18_outdoor_gym	
166	85_2007_20_migration	
167	85sl_2007_20_migration	
168	86_2007_20_population_dens	
169	87_2007_20_demograph_depend	
170	87sl_2007_20_demograph_depend	
171	88_2016_20_urban	
172	88sl_2016_20_urban	
173	89_2007_20_population	
174	90_2007_20_nat_increase	Correlation coefficient below -0,9 with var. 97
175	90sl_2007_20_nat_increase	
176	91_2007_20_births	
177	91sl_2007_20_births	
178	92_2007_20_deaths	
179	92sl_2007_20_deaths	
180	93_2007_20_femin	
181	94_2010_20_population_19_24	
182	94sl_2010_20_population_19_24	
183	95_2010_20_population_25-44	
184	96_population_45-64	
185	97_population_over_65	
186	97sl_population_over_65	Correlation coefficient over 0,9 with var. 87sl
187	98_health_dep	
188	98sl_health_dep	
189	99_2007_20_pharm	
190	99sl_2007_20_pharm	
191	100_2008_20_family_all	
192	100sl_2008_20_family_all	

193	101_2008_20_social_assist_inc	Correlation coefficient over 0,9 with var. 102
194	101sl_2008_20_social_assist_inc	
195	102_2009_19_social_assit	
196	102sl_2009_19_social_assit	Correlation coefficient over 0,9 with var. 101sl
197	103_2007_20_unemp	
198	103sl_2007_20_unemp	
199	104_2007_20_employed	
200	104sl_2007_20_employed	
201	105_2007_19_primary_educ_ratio	
202	105sl_2007_19_primary_educ_ratio	
203	106_2010_19_pupils_primary	
204	106sl_2010_19_pupils_primary	
205	107_2007_19_preschool_educ	
206	107sl_2007_19_preschool_educ	
207	108_2014_18_nursery_places	
208	108sl_2014_18_nursery_places	
209	109_2010_20_State Forests	
210	109sl_2010_20_State Forests	
211	110_2010_20_NP_forests	
212	110sl_2010_20_NP_forests	
213	111_2011_20_private_forests	
214	111sl_2011_20_private_forests	
215	112_2007_20_agric_gmina_board	
216	112sl_2007_20_agric_gmina_board	
217	113_2007_20_high_educ_board	
218	113sl_20_high_educ_board	
219	114_mpzp_forest_non_forest	
220	114sl_mpzp_forest_non_forest	
221	115_2009_20_mpzp_agric_non_agric	
222	115sl_2009_20_mpzp_agric_non_agric	
223	116_2009_20_mpzp_share	
224	116sl_2009_20_mpzp_share	
225	117_2009_20_decision_on_publ_invest	
226	117sl_2009_20_decision_on_publ_invest	
227	118_2009_20_decision_on_single_house	
228	118sl_2009_20_decision_on_single_house	
229	119_2009_20_decision_on_multi_house	
230	119sl_2009_20_decision_on_multi_house	
231	120_2009_20_suikzp_agric_non_agric	
232	120sl_2009_20_suikzp_agric_non_agric	
233	121_2009_20_suikzp_forest_non_forest	
234	121sl_2009_20_suikzp_forest_non_forest	
235	122_2007_20_private_forest_removals	
236	122sl_2007_20_private_forest_removals	
237	123_2007_20_forest_cov	

238	123sl_2007_20_forest_cov	
239	124_2012_14_agric_land_capita	
240	124sl_2012_14_agric_land_capita	
241	125_2012_14_agric_lands	
242	125sl_2012_14_agric_lands	
243	126_2012_14_recreational areas	
244	126sl_2012_14_recreational areas	
245	127_2012_14_roads	
246	127sl_2012_14_roads	
247	128_2012_14_other_transport	
248	128sl_2012_14_other_transport	
249	129_2012_14_arable_lands	
250	129sl_2012_14_arable_lands	
251	130_2012_14_arable_lands	
252	130sl_2012_14_arable_lands	
253	131_2012_14_pastures	
254	131sl_2012_14_pastures	
255	132_2012_14_forests	
256	132sl_2012_14_forests	
257	133_2012_14_urbanized	
258	133sl_2012_14_urbanized	
259	134_2012_14_built_up	
260	134sl_2012_14_built_up	Correlation coefficient over 0,9 with var. 133sl
261	135_2012_14_indust	
262	135sl_2012_14_indust	
263	136_2012_14_fl_water	
264	136sl_2012_14_fl_water	
265	137_2012_14_water_bodies	
266	137sl_2012_14_water_bodies	
267	138_2012_14_wastelands	
268	138sl_2012_14_wastelands	
269	139_2012_14_urban_PAs	
270	139sl_2012_14_urban_PAs	
271	140_2010_av_agr_land	
272	141_2010_private_farms_over_15	Correlation coefficient over 0,9 with var. 143
273	142_2010_agr_hold_to_1	
274	143_2010_agr_hold_over_10	
275	144_2010_agric_in_private_farms	
276	145_2010_private_agric_share	
277	146_2010_private_meadows_share	
278	147_2010_fallows	
279	148_2010_perm_crops	
280	149_2010_tractors_private	
281	150_2010_tractors_ha	
282	151_2010_cattle	

283	152_2010_pigs	
284	153_2010_poultry	
285	154_2010_farms_up_1	
286	155_2010_farms_1_5	
287	156_2010_farms_5_10	
288	157_2010_farms_over_10	
289	158_2010_mineral_fert	
290	159_2010_nitro_fertilizers	Correlation coefficient over 0,9 with var. 160
291	160_2010_phos_fertilizers	
292	161_2010_agric_income_farm	
293	162_2010_retirement_income_farm	
294	163_2010_cereals	
295	164_2010_maize	
296	165_2010_potatoes	
297	166_2010_indust_crops	
298	167_2010_sugar_beets	
299	168_2010_rapseed	
300	169_2010_edible_puls	
301	170_2010_vegetables	
302	171_2007_20_PA_share	
303	171sl_2007_20_PA_share	
304	172_2007_20_NP_share	
305	173_2007_20_reserves_share	
306	173sl_2007_20_reserves_share	
307	174_2007_20_LP_share	
308	174sl_2007_20_LP_share	
309	175_2007_20_PLA_share	
310	175sl_2007_20_PLA_share	
311	176_2007_20_water_tr_capacity	
312	176sl_2007_20_water_tr_capacity	
313	177_2007_20_water_tr_popuation	
314	177sl_2007_20_water_tr_popuation	
315	178_2007_20_water_BZT	Correlation coefficient over 0,9 with var. 182
316	178sl_2007_20_water_BZT	
317	179_2007_20_water_ChZT	Correlation coefficient over 0,9 with var. 182
318	179sl_2007_20_water_ChZT	
319	180_2007_20_water_suspension	Correlation coefficient over 0,9 with var. 182
320	180sl_2007_20_water_suspension	
321	181_2007_20_water_nitrogen	Correlation coefficient over 0,9 with var. 182
322	181sl_2007_20_water_nitrogen	
323	182_2007_20_water_phosph	
324	182sl_2007_20_water_phosph	
325	183_2007_20_treated_wastewater_capita	

326	183sl_2007_20_treated_wastewater_capita	
327	184_2007_20_wastewater_facilit_popul	Correlation coefficient over 0,9 with var. 29
328	184sl_2007_20_wastewater_facilit_popul	
329	185_2007_20_treated_wastewater_share	
330	185sl_2007_20_treated_wastewater_share	
331	186_2007_20_green_areas_share	
332	186sl_2007_20_green_areas_share	
333	187_2007_19_real_estate_green	
334	187sl_2007_19_real_estate_green	

[1]Note: We constructed correlation tables of all the variables (first – normalized, then standardized ones) and we inspected pairs of variables with correlation coefficients bigger than |0,900|. We deleted one of such two variables if: 1) both variables were referred to the same factor(s) described in the source papers (we excluded the one less often used in the papers), or 2) substance and processual indicators of the same variable were correlated (we excluded the processual one).

Supplementary Information 4 - methodology

Step D (all the referred steps are presented on Fig. 1. in the main text)

In order to meet assumptions for the PCA/EFA (Salama, Hassanien and Fahmy 2010), we normalised all the values, also leaving a set of raw values for their further standardisation. This was crucial for trend values that included both positive and negative values, and normalization or standardisation processes generated different pairwise correlations within the datasets.

Step G

Before every PCAs/EFAs, we were performing test analyses to determine the number of PCs/Fs, using a method of scree plots interpretation (Abdi and Williams 2010). The interpretation of PCs was carried out on rotated component matrixes (a Varimax rotation applied; Jolliffe and Cadima 2016)), while Fs – on a pattern matrix of factors ((a principal axis method, Promax rotation applied, Kappa = 4; Yong and Pearce 2013). If the interpretative power of the results were poor, we were redetermining the number of PCs/Fs, by inspecting the total variance explained (Abdi and Williams 2010) and using results of other procedures as benchmarks. Finally, we were saving component / factor scores for each of the units analysed (that is, for Lesser Poland municipalities).

We used the following sequence of criteria for the dimension reduction of variables: 1) loadings for the determined PCs/Fs lower than |0,2| → 2) the loadings lower than |0,3| → 3) correlation coefficient with another variable of the same factor higher than |0,800|. Sometimes the KMO target was not achieved despite a significant reduction in a number of variables. Then, the decisions were made to stop the procedure, especially as the KMO threshold we had applied was liberal anyways. We found a KMO=0,5 as already acceptable, considering the large difference in character of the variables and approval of such a threshold by some authors (Yong and Pearce 2013).

In <*2-step EFA*> procedures, we extracted factors using a principal component analysis method. A principal axis method failed as communalities of some variables exceeded 1.0

Step I

For clustering, we used Ward's method, as it 1) has already been introduced into the field of conservation conflict (Kubo and Shoji 2016, Loc et al. 2021), 2) allows one to obtain potentially similar numbers of units across clusters, and 3) offers the possibility of a visual inspection of the clustering process.

When establishing the number of clusters, as a matter of reference, we were also analysing distance coefficients in the agglomeration schedule, looking for a drop in distance between the coefficients (Yim and Ramdeen 2015); however, it either provided too general final result (3 or 4 clusters only, with Kraków clustered with other municipalities) or no visible gap between the coefficients were detected.

Step J

The municipalities were ultimately assigned to a cluster group that had been assigned most often across different versions of the analysis. However, in the case of seven municipalities, an arbitrary decision had to be made due to the equal number of the two most frequent assignments. Ultimately, the units were classified in the 'rural loc. under transition' cluster as best describing boundary objects.

Step N

For a case study, we coded the data downloaded from Google search results. Most of the codes were assigned based on analysis of a Google title or description only, but in some cases, scanning the whole content was necessary. We used a single search record as a measurement unit, but for some of PDF/DOC files, we found acceptable to assign more than one code to the search record, as they contained more complex descriptions of the conflict context, addressing more than one conflict issue. In five cases, the records were coded as non-relevant: 1) they did not actually concern TNP or 2) conflicts, 3) they did not match the set timeframe, 4) they did not contain any content apart from the bibliographic note, and 5) they were written in another language than Polish.

Supplementary Information 5 – results

Summary of the analysis (see tables below for details)

Out of 18 analyses performed, in 13 cases the KMO values exceeded 0,5, which allowed the interpretation of the PCA/EFA results. Measures were unacceptable for every <*μ&a; all var.*> analysis, despite the exclusion of 135+ variables throughout the procedure. The KMO values were acceptable but still low for <*2-step; pre-def.*> procedures as they never exceeded 0,6. For <*1-step PCAs/EFAs*>, the measures were generally higher, especially for PCAs/EFAs performed only on variables classified as social. The highest KMO value (0,832) was recorded for a <*μ only 1-step 'social' EFA*>.

For every <*2-step pre-def. PCAs/EFAs*> or <*all var. PCAs/EFAs*>, a scree plot analysis allowed for clear distinguishment of 5 PCs/Fs. This was also the case for <*1-step; pre-def.*> analyses on variables classified as economic. For other groups of determinants, the number of PCs/Fs varied across the analyses (4 or 5 social PCs/Fs; 2 or 4 institutional PCs/Fs; 2-6 environmental PCs/Fs). Also, decisions made on the final number of these PCs/Fs were less evident based on scree plot analysis, and they were often adjusted with the use of other criteria.

In 10 cases, the factor scores from the PCA/EFA procedures allowed for depicting clusters of Lesser Poland municipalities similar in terms of their PA conflict determinants. In three other cases, the clustering procedures were performed, but they did not end up with distinguishing Kraków a separate cluster, which was a requirement for proceeding with the interpretation. The number of clusters varied from 6 <*μ&a; 1-step, pre-def. PCA*> to 13 <*μ only; 1-step, pre-def., EFA*>.

	Normalized data											
	With trend data						Without trend data					
	PCA			FA			PCA			FA		
	All	Pre-def. 1-level	Pre-def. 2-level	All	Pre-def. 1-level	Pre-def. 2-level	All	Pre-def. 1-level	Pre-def. 2-level	All	Pre-def. 1-level	Pre-def. 2-level
How many steps	12	Ec-2<br>S-2<br>I-2<br>En-2	2	12	Ec-4<br>S-2<br>I-4<br>En-2	2	3	Ec-2<br>S-2<br>I-2<br>En-2	2	3	Ec-2<br>S-2<br>I-2<br>En-2	1
Total no. of excluded variables	142	Ec-0<br>S-0<br>I-0<br>En-0	0	142	Ec-42<br>S-0<br>I-4<br>En-0	0 (46)	11	Ec-0<br>S-0<br>I-0<br>En-0	0	11	Ec-0<br>S-0<br>I-0<br>En-0	0
Final KMO measure	0,464	Ec-0,585<br>S-0,711<br>I-0,689<br>En-0,557	0,557	0,464	Ec-0,546<br>S-0,606<br>I-0,514<br>En-0,589	0,561	0,606	Ec-0,738<br>S-0,799<br>I-0,725<br>En-0,712	0,525	0,606	Ec-0,718<br>S-0,832<br>I-0,616<br>En-0,719	0,464
No. of clusters	n/a	6	7	n/a	8	7	9	NC	11	NC	13	n/a
Spatial distribution of clusters	n/a	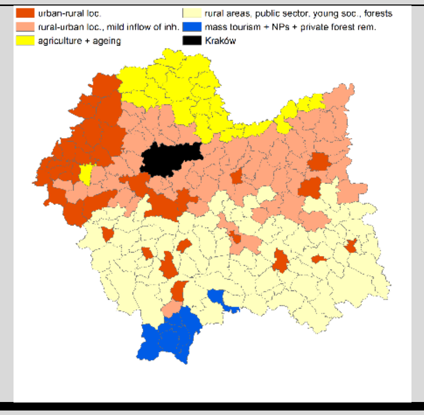	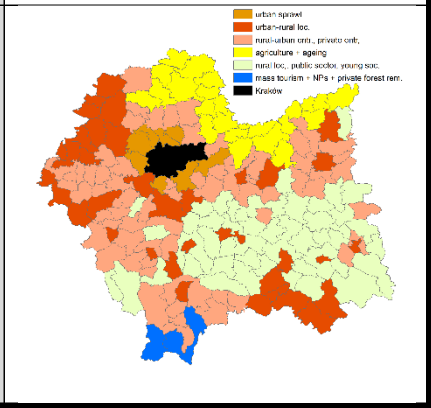	n/a	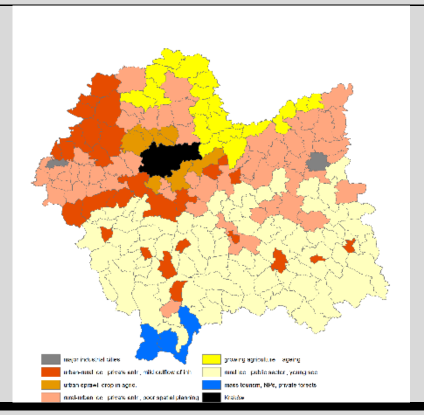	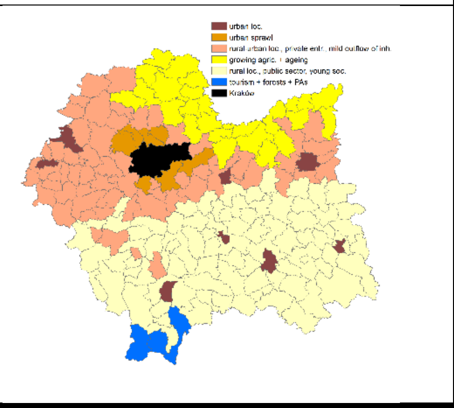	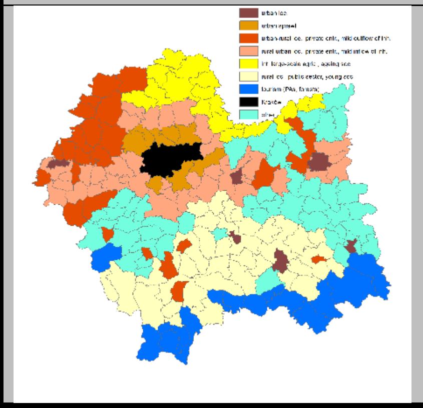	n/a	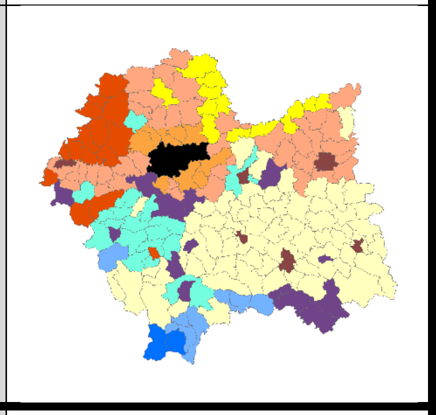	n/a	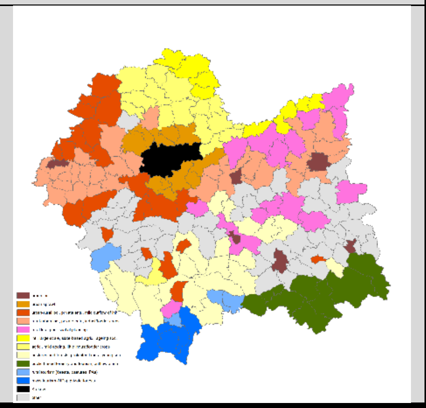	n/a

Clusters[1]	With trend PCA All	PCA 1-level		PCA 2-level		FA All	FA 1-level		FA 2-level		Without trend PCA All		PCA 1-level	PCA 2-level	FA All	FA 1-level		FA 2-level
Kraków	n/a	(drop in water pollution, spatial planning dev., poor cult. activity)<br>3rd (out of "urban-rural loc." cluster)	1	4th (out of "urban sprawl" cluster)	1	n/a	(drop in water pollution)<br>5th (out of "urban sprawl" cluster)	1	4th (out of "urban sprawl" cluster)	1	6th (out of "urban loc." cluster)	1	n/a	YES	n/a	11th (out of "urban loc." cluster)	1	n/a
Industrial cities (growing pollution, mild outflow, ageing)	n/a					n/a	4th	2	3rd	8	1st	6	n/a	YES	n/a	2nd	6	n/a
Urban loc.	n/a	1st	34	2nd	38	n/a							n/a		n/a			n/a
Urban-rural loc., private entr., mild outflow of inh.	n/a					n/a	2nd	27			3rd	25	n/a	YES	n/a	5th	21	n/a
Rural-urban loc., private entr., industrial/fodder crops	n/a			3rd	61	n/a	1st	49	1st	58	2nd	33	n/a		n/a	1st	27	n/a
Urban sprawl	n/a	2nd	54	1st	8	n/a	(drop in agriculture)<br>3rd	7	2nd	7	4th	8	n/a	YES (small-scale, private-based agriculture)	n/a	4th	11	n/a
Agriculture, mild ageing, little	n/a	1st	21	1st	23	n/a	(growing agric., drop in empl. in administration sector)		(growing agric., drop in empl. in administration sector)		1st	17	n/a		n/a	1st	15	n/a

	industrial/fodder crops																		
	Intensive, large-scale public-based agr., ageing soc.,	n/a					n/a	1st	19	1st	26			n/a	YES	n/a	7th	9	n/a
	Forests, pastures, landscape prot., young soc., empl. in construction sect.	n/a	1st	66	(partially within rural-urban loc.)		n/a	1st	74	1st	79	5th	34	n/a	INSTEAD - rural areas with higher role of public sector	n/a	8th	18	n/a
	Rural areas w. poor spatial planning	n/a			1st	48	n/a	(together with rural-urban loc.)				1st	44	n/a		n/a	6th	15	n/a
	other	n/a					n/a							n/a	INSTEAD - rural areas with higher role of private sector	n/a	1st	43	n/a
	Public-based forestry & tourism, outflow of inh.	n/a			(partially within urban-rural loc.)		n/a					1st	14	n/a	INSTEAD – a cluster similar to urban-rural loc.	n/a	9th	8	n/a
	Rural tourism, PAs, forests	n/a			(partially within rural-urban loc.)		n/a							n/a	YES	n/a	3rd	4	n/a
	Mass tourism, NPs, private forest rem.	n/a	1st	6	1st	3	n/a	1st	3	1st	3			n/a	YES	n/a	1st	4	n/a
Principal components / factors[2]	"urban" (spec. serv., infr., pop. density, empl., tourism, social cap., greenery, pollution, indust.)	n/a	n/a		1.		n/a	n/a		2.		1.		n/a	1.	1.	n/a		n/a
	"agric." (empl. in admin., ageing, large prop., euthroph.)	n/a	n/a		2. (drop in empl. in administration)		n/a	n/a		1. (growing agriculture, drop in empl. in administration, milder ageing)		2.		n/a	2. (medium-sized properties)	2.	n/a		n/a
	"small entr." (little public aid, working class, poverty)	n/a	n/a		5. (high cultural activity)		n/a	n/a		4. (ageing)		3.		n/a	5. *reversed* – public sector, large-scale properties	3	n/a		n/a
	"tourist" (grant app., poor gas infr., NPs, private forest rem.)	n/a	n/a		4.		n/a	n/a		3. (growing tourism)		4. (forests in general)		n/a	4.	4. (forests in gen.)	n/a		n/a
	"sprawl" (building inv., inflow of inh., standards of living)	n/a	n/a		3.		n/a	n/a		5.		5.		n/a	3. *reversed* – outflow of inh. + social problems	5	n/a		n/a
Economic PCs/F[2]s	Urban econ. (infr., spec. services, own revenue)	n/a	1. (drop in own revenue)		n/a		n/a	1.		n/a		n/a		1.	n/a	n/a	1.		n/a
	Agric. economy (admin. sector)	n/a	3. (drop in admin. sector)		n/a		n/a	2. (drop in admin. sector)		n/a		n/a		3.	n/a	n/a	2.		n/a
	Tourism industry (grant app., poor gas infr.)	n/a	2.		n/a		n/a	3.		n/a		n/a		2.	n/a	n/a	3.		n/a
	Public sector (revenue)	n/a	5.		n/a		n/a	5.		n/a		n/a		4.	n/a	n/a	4.		n/a
	Residential inv. (entr., spec. serv.)	n/a	4.		n/a		n/a	4.		n/a		n/a		5.	n/a	n/a	5.		n/a

Social PCs/Fs[2]	Urban society (pop. density, empl., femin., services, social cap., tourism, well-being)	n/a	1. (rise in demographic dependency)	n/a	n/a	2.	n/a	n/a	1.	n/a	n/a	1.	n/a
	Ageing	n/a	2.	n/a	n/a	1. (high drop in empl. in construction sector)	n/a	n/a	2.	n/a	n/a	2.	n/a
	Working class	n/a		n/a	n/a	3. (standards of liv., rise in empl. in administration)	n/a	n/a		n/a	n/a	3.	n/a
	Tourism	n/a	4.	n/a	n/a	5.	n/a	n/a	n/a	n/a	n/a	4.	n/a
	Inflow of inh. (standards of liv.)	n/a	3. (high employment)	n/a	n/a	4. (natural increase)	n/a	n/a	3. *reversed* (social assist., unemp.)	n/a	n/a	5.	n/a
	Cultural activity	n/a	5.	n/a	n/a	n/a	n/a	n/a	4.	n/a	n/a	n/a	n/a
Institutional PCs/Fs[2]	Large properties	n/a	1.	n/a	n/a	1.	n/a	n/a	1.	n/a	n/a	1.	n/a
	Poor spatial planning	n/a	2.	n/a	n/a	2.	n/a	n/a	2.	n/a	n/a	2.	n/a
	Strategic decisions on land-use change	n/a	3.	n/a	n/a	n/a	n/a	n/a	n/a	n/a	n/a	n/a	n/a
	Spatial planning dev.	n/a	4.	n/a	n/a	n/a	n/a	n/a	n/a	n/a	n/a	n/a	n/a
	PA share	n/a	n/a	n/a	n/a	3. (hotel permits)	n/a	n/a	n/a	n/a	n/a	3.	n/a
	NP share	n/a	n/a	n/a	n/a		n/a	n/a	n/a	n/a	n/a	4. (hotel permits)	n/a
	Small properties + private forests	n/a	n/a	n/a	n/a	4.	n/a	n/a	n/a	n/a	n/a	n/a	n/a
Environmental PCs/Fs[2].	Urban env. (greenery, pollution, industry, infr.)	n/a	1.	n/a	n/a	1.	n/a	n/a	1.	n/a	n/a	1.	n/a
	Growing agric.	n/a	n/a	n/a	n/a	3.	n/a	n/a	n/a	n/a	n/a	n/a	n/a
	Agric. env (eutrophication)	n/a	2. *reversed* (forests)	n/a	n/a	2.	n/a	n/a	2.	n/a	n/a	2.	n/a
	Industrial/fodder crops	n/a	5.	n/a	n/a		n/a	n/a	n/a	n/a	n/a	5.	n/a
	Growing urbanization	n/a		n/a	n/a	n/a	n/a	n/a	n/a	n/a	n/a	n/a	n/a
	Growing water pollution	n/a	3.	n/a	n/a	4.	n/a	n/a	n/a	n/a	n/a	n/a	n/a
	Wastelands + NPs + private forest removals	n/a	4.	n/a	n/a	5.	n/a	n/a	3.	n/a	n/a	6.	n/a
	Forests, LPs	n/a	n/a	n/a	n/a	n/a	n/a	n/a	n/a	n/a	n/a	4.	n/a
	Pastures, other PAs	n/a	n/a	n/a	n/a	n/a	n/a	n/a	n/a	n/a	n/a	3.	n/a

[1] clusters, order of their distinguishment and number of municipalities assigned to a cluster
[2] the numbers indicate the PCs/Fs order in terms of the share of total variance explained

		Standardized data - with trend data								
		PCA					FA			
		All	Pre-def. 1-level		Pre-def. 2-level		All	Pre-def. 1-level	Pre-def. 2-level	
How many steps		12	Ec-2<br>S-2<br>I-2<br>En-2		2		12	Ec-6<br>S-2<br>I-4<br>En-2	2	
Total no. of excluded variables		137	Ec-0<br>S-0<br>I-0<br>En-0		0		137	Ec-45<br>S-0<br>I-11<br>En-0	0 (46)	
Final KMO measure		0,473	Ec-0,607<br>S-0,711<br>I-0,689<br>En-0,582		0,546		0,473	Ec-0,559<br>S-0,587<br>I-0,607<br>En-0,596	0,529	
No of clusters		n/a	6		7		n/a	NC	7	
Spatial distribution of clusters		n/a	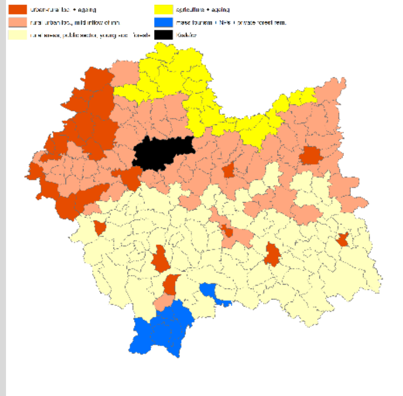		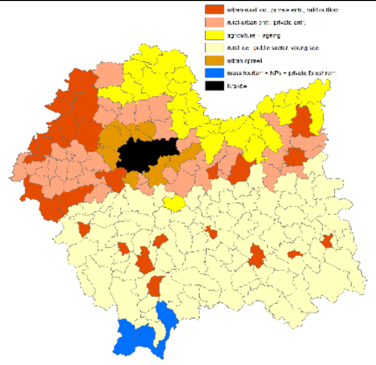		n/a	n/a	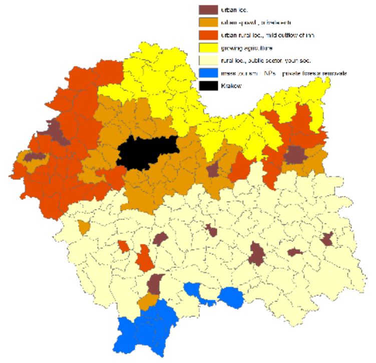	
Clusters[1]	Kraków	n/a	*(drop in water pollution, spatial planning dev., poor cult. activity)*<br>3rd (out of "urban-rural loc." cluster)	1	4th (out of "urban sprawl" cluster)	n/a	n/a	n/a	4th (out of "urban loc." cluster)	1
	Industrial cities (growing pollution, mild outflow, ageing)	n/a					n/a	n/a	3rd	10
	Urban loc.	n/a	1st	24	3rd	27	n/a	n/a		
	Urban-rural loc., private entr., mild outflow of inh.	n/a					n/a	n/a	2nd	32
	Rural-urban loc., private entr., industrial/fodder crops	n/a	2nd	60	1st	34	n/a	n/a	1st	31

	Urban sprawl	n/a			2nd	8	n/a	n/a		
	Agriculture, mild ageing, little industrial/fodder crops	n/a	1st	19	1st	26	n/a	n/a	1st	26
	Intensive, large-scale public-based agr., ageing soc.,	n/a					n/a	n/a		
	Forests, pastures, landscape prot., young soc., empl. in construction sect.	n/a	1st	72	1st	83	n/a	n/a	1st	75
	Rural areas w. poor spatial planning	n/a	(together with rural-urban loc.)				n/a	n/a		
	other	n/a					n/a	n/a		
	Public-based forestry & tourism, outflow of inh.	n/a					n/a	n/a		
	Rural tourism, PAs, forests	n/a					n/a	n/a		
	Mass tourism, NPs, private forest rem.	n/a	1st	6	1st	3	n/a	n/a	1st	7
Principal components / factors[2]	"urban" (spec. serv., infr., pop. density, empl., tourism, social cap., greenery, pollution, indust.)	n/a	n/a		1.		n/a	n/a	2.	
	"agric." (empl. in admin., ageing, large prop., euthroph.)	n/a	n/a		2. (drop in empl. in administration)		n/a	n/a	1. (drop in empl. in administration, poor spatial planning)	
	"small entr." (little public aid, working class, poverty)	n/a	n/a		5. *reversed* (public sector, high revenue)		n/a	n/a	5. *reversed* (public sector, no ageing)	
	"tourist" (grant app., poor gas infr., NPs, private forest rem.)	n/a	n/a		4.		n/a	n/a	3. (growing tourism)	
	"sprawl" (building inv., inflow of inh.,	n/a	n/a		3.		n/a	n/a	4.	

	standards of living)						
Economic PCs/Fs[2]	Urban econ. (infr., spec. services, own revenue)	n/a	1. (drop in own revenue)	n/a	n/a	1.	n/a
	Agric. economy (admin. sector)	n/a	3. (drop in admin. sector)	n/a	n/a	2. (drop in admin. sector)	n/a
	Tourism industry (grant app., poor gas infr.)	n/a	2.	n/a	n/a	3.	n/a
	Public sector (revenue)	n/a	5.	n/a	n/a	5.	n/a
	Residential inv. (entr., spec. serv.)	n/a	4.	n/a	n/a	4.	n/a
Social PCs/Fs[2]	Urban society (pop. density, empl., femin., services, social cap., tourism, well-being)	n/a	1. (rise in demographic dependency)	n/a	n/a	2.	n/a
	Ageing	n/a	2.	n/a	n/a	1. (high drop in empl. in construction sector, working class, standards of liv.)	n/a
	Working class	n/a		n/a	n/a	3. (only rise in empl. in administration, standards of liv)	n/a
	Tourism	n/a	4.	n/a	n/a	5. (agrotourism)	n/a
	Inflow of inh. (standards of liv.)	n/a	3. (high employment)	n/a	n/a	4. (natural increase)	n/a
	Cultural activity	n/a	5.	n/a	n/a	n/a	n/a
Institutional PCs/Fs[2]	Large properties	n/a	1.	n/a	n/a	1.	n/a
	Poor spatial planning	n/a	2.	n/a	n/a	2.	n/a
	Strategic decisions on land-use change	n/a	n/a	n/a	n/a	n/a	n/a
	Spatial planning dev.	n/a	n/a	n/a	n/a	n/a	n/a
	PA share	n/a	n/a	n/a	n/a	n/a	n/a
	NP share	n/a	n/a	n/a	n/a	n/a	n/a
	Small properties + private forests	n/a	n/a	n/a	n/a	n/a	n/a
Environmental PCs/Fs[2]	Urban env. (greenery, pollution, industry, infr.)	n/a	1.	n/a	n/a	1.	n/a
	Growing agric.	n/a	n/a	n/a	n/a	n/a	n/a
	Agric. env (eutrophication)	n/a	2. *reversed* (forests)	n/a	n/a	2. *reversed*	n/a

	Industrial/fodder crops	n/a	5.	n/a	n/a	n/a	n/a
	Growing urbanization	n/a		n/a	n/a	n/a	n/a
	Growing water pollution	n/a	3.	n/a	n/a	n/a	n/a
	Wastelands + NPs + private forest removals	n/a	4.	n/a	n/a	n/a	n/a
	Forests, LPs	n/a	n/a	n/a	n/a	n/a	n/a
	Pastures, other PAs	n/a	n/a	n/a	n/a	n/a	n/a

[1] clusters, order of their distinguishment and number of municipalities assigned to a cluster
[2] the numbers indicate the PCs/Fs order in terms of the share of total variance explained

B. The role of trend values in the final results

In a synthetic stage, the role of trend values was reduced to deepening interpretation of PCs/Fs or clusters that were already established in procedures that did not use any trend data. However, PCs/Fs that describe processes rather than conditions (e.g., 'sprawl' PCs/Fs and their analogues in certain groups of determinants) explained larger share of total variance in <μ&a> procedures than in <μ only> analyses. Similarly, 'sprawl' clusters were formed at earlier stages of the <μ&a> clustering processes, compared to their <μ only> analogues.

More visible role of trend variables concerned <1-step; pre-def.> procedures at the level of certain groups of determinants. For example, distinguishing 'growing water pollution' PC/F in <1-step, pre-def. – env.; μ&a> procedures allowed for capturing a separate cluster of cities with growing industry. In <μ only> versions of the analyses, industry was too closely connected with 'urban' PCs/Fs to form a separate one. Conversely, the strong role of 'sprawl' PCs/Fs in <μ&a> procedures diminished the importance of variables describing social problems (share of people benefiting from social assistance, unemployment rate, etc.), being negatively correlated with the precious. Consequently, a separate 'social problems' PC was distinguished only in a <1-step; pre-def. – soc., μ only PCA> procedure, where variables that typically loaded 'inflow' PCs/Fs were less numerous.

Supplementary Information 6 – Internet content coding

Tab. S6.1. Code tree of conflict issues in Tatra National Park based on Internet content analysis

Name of the code	No of instances
Total number of coded records	620
Non-relevant records	447
Relevant records	173
Land-use conflict in general	1
Variety of stakeholders in general	1
Stakeholders of a Morskie Oko road	2
grazing	1
„oscypek" (local smoked cheese) as a cultural heritage	1
Conflicts with local authorities	0
Over a NP buffer zone	1
Over road maintenance	0
The use of road salt	1
Over a Morskie Oko road	1
Over a cross on the summit of Rysy	1
Tourist-related	0
Tourism vs conservation in general	9
Mass character of tourism	2
Consequences of tourism	0
trampling	1
Littering	1
synanthropization of fauna	6
Variety of forms of tourism	1
Over an Orla Perć high-mountain track equipment	1
Over a horse transport on the Morskie Oko road	13
Over a New Years' Eve event in Zakopan	4
Over a rebuilt of a chapel in Chochołowska valley	1
Over sleigh parties by the border of TNP	3
Over running sport events in TNP	1
Over ski touring/freeriding in TNP	4
vs Alpine skiers	1
Conflicts with climbers	4
Over climbing camps in TNP	1
Over climbing registers	1

Over climbing in Western Tatras	3
Property issues	0
Over a Central Sports Centre hotel by the borders of TNP	5
Over the properties of Polish Tourist and Sightseeing Society in TNP	21
Over skiing infrastructure in TNP	2
Over a ski complex on the slopes of Kasprowy Wierch	54
Transboundary context of the conflict	1
Over forest management in TNP	0
Social vs. conservation functions of forests	3
Virtual character of the conflict	1
Over private forests in TNP	1
Over forests of a Forest Community of 8 Eligible Villages in Witów	3
Conflicts with local communities	1
Hindrances to economic activities in general	1
Over a cross illumination on the summit of Giewont in TNP	3
Over private properties in TNP	9
Fence construction	1

Tab. S6.2. A full list of URLs with content classified as relevant:

No.	URL
1	http://agro.icm.edu.pl/agro/element/bwmeta1.element.agro-6eaca267-f1b0-4ea1-9110-cec751d85617/c/13.pdf
2	http://alw.pl/category/reklama/
3	http://cejsh.icm.edu.pl/cejsh/element/bwmeta1.element.hdl_11089_17045
4	http://cejsh.icm.edu.pl/cejsh/element/bwmeta1.element.hdl_11089_17045/c/fsgo_8_Wioletta_Kucina_185_210.pdf
5	http://denali.geo.uj.edu.pl/publikacje.php?pdf=000191_10&notka=TWFyY2luIFJlY2hjaW59c2tpIDIwMTMgUm99en1uaWNlIHcgZm9ybWFjaCBnb3Nwb2Rhcm93YW5pYSBuYSBvYnN6YXJ6ZSBUYXRyemFufXNraWVnbyBQYXJrdSBOYXJvZG93ZQ==
6	http://krakow.rdos.gov.pl/niedzwiedz-w-beskidzie-malym
7	http://nietylko.design/wp-content/uploads/2016/05/Nie-tylko-design-transkrypcja-010.pdf
8	http://orka2.sejm.gov.pl/StenoInter7.nsf/0/7F1A0DBDE3073E8CC1257B18007EEC81/%24File/34_b_ksiazka.pdf
9	http://paek.ukw.edu.pl/wydaw/vol25/myga_piatek_jankowski.pdf
10	http://pza.org.pl/download/312575.pdf
11	http://pza.org.pl/download/3146292.pdf
12	http://spinanie.pl/spor-pza-tpn-episkopat-proponuje-mediacje/
13	http://tpn.pl/filebrowser/biuletyn/biuletyn_tpn_3_24_2012.pdf

14	http://wspinanie.pl/wp-content/uploads/2015/06/List-KW-Warszawa-do-Delegatow-WZD-PZA-oraz-Zarzadu-PZA-05.2015-i-zalaczniki.pdf
15	http://www.barbarzynca.org/1-20-2014/spor-o-oscypka-tradycja-jako-praktykowanie-lokalnosci
16	http://www.dbc.wroc.pl/Content/25652/Kulczyk-Dynowska_Turystyka_w_Gminach_Tatrzanskich_2014.pdf
17	http://www.folia-turistica.pl/attachments/article/430/FT_22_2010.pdf
18	http://www.hotelinfo24.pl/news,12717,2,Tradycyjne_zakopianskie_kuligi_moga_wrocic_na_Droge_pod_Reglami.html
19	http://www.hydro.geo.uj.edu.pl/~j.pociask/40_tendencje.pdf
20	http://www.npl.ibles.pl/sites/default/files/aneks_2.pdf
21	http://www.tur-info.pl/a/58675,,tpn-pkl-demontaz-kolejka-kasprowy.html
22	http://www.unesco.pl/sourceskraj/Kultura_a_zrownowazony_rozwoj.pdf
23	http://www.wielkopolski.ptl.pl/uploads/TEST%20TK/Publikacje/Ochrona-zwierzat.pdf
24	http://yadda.icm.edu.pl/yadda/element/bwmeta1.element.ekon-element-000171504686
25	https://24tp.pl/?mod=news&strona=1&kat=7&id=9273&typ=w
26	https://24tp.pl/n/17991
27	https://agro.icm.edu.pl/agro/element/bwmeta1.element.agro-182ba272-4c91-4d59-9b27-72d0369ac651/c/giergiczny_zwijacz-kozica.pdf
28	https://archiwum.podhaleregion.pl/index.php/z-podhala/23179-spor-o-kolejke-na-kasprowy-wierch-na-linii-tpn-pkl
29	https://archiwum.rp.pl/artykul/129109-Radni-Zakopanego-kontra-premier.html
30	https://bazhum.muzhp.pl/media/files/Prawne_Problemy_Gornictwa_i_Ochrony_Srodowiska/Prawne_Problemy_Gornictwa_i_Ochrony_Srodowiska-r2016-t-n2/Prawne_Problemy_Gornictwa_i_Ochrony_Srodowiska-r2016-t-n2-s99-117/Prawne_Problemy_Gornictwa_i_Ochrony_Srodowiska-r2016-t-n2-s99-117.pdf
31	https://bip.zakopane.eu/zalacznik/14684
32	https://businessinsider.com.pl/wiadomosci/fiasko-rozmow-ws-kolejki-na-kasprowy-wierch/vbk6n8d
33	https://cotg.pttk.pl/wp-content/uploads/2020/05/bezpieczenstwo_aspekty.pdf
34	https://docplayer.pl/49580505-Folia-geographica-socio-oeconomica-8-wioletta-kucina.html
35	https://docplayer.pl/69514614-Dobrostan-koni-pracujacych-na-trasie-do-morskiego-oka.html
36	https://docplayer.pl/amp/221036699-Tatrzanski-park-narodowy-konflikt-ochrony-przyrody-i-turystyki-1.html
37	https://dspace.uni.lodz.pl/bitstream/handle/11089/33938/121-128_FELCZAK.pdf?sequence=1&isAllowed=y
38	https://dyskusje24.pl/szukaj/forum/kolejka+na+kasprowy
39	https://dziennikpolski24.pl/goralskie-kuligi-pod-reglami-zakopane-rozmawia-z-tpn/ar/3292840
40	https://dziennikpolski24.pl/spor-o-droge-do-morskiego-oka-tpn-powinien-placic-za-naprawy/ar/10423650
41	https://dziennikpolski24.pl/tag/tatrzanski-park-narodowy
42	https://encyklopedia.interia.pl/geografia-nauki-pokrewne/krainy-geograficzne/news-tatrzanski-park-narodowy,nId,1996928
43	https://forsal.pl/artykuly/1483348,tatrzanskie-schroniska-dolina-koscieliska-kondratowa-pttk-tpn.html
44	https://gazetakrakowska.pl/tpn-oszukal-z-powodu-konia/ar/159766

45	https://gazetakrakowska.pl/tpn-szuka-pieniedzy-wiec-postanowil-zarabiac-min-na-reprezentantach-polski/ar/c1-14950898
46	https://gazetakrakowska.pl/znow-spor-o-konie-w-morskim-oku/ar/339030
47	https://geex.x-kom.pl/wiadomosci/hulajnoga-elektryczna-nad-morskie-oko-czy-zastapia-one-konie-i-meleksy/
48	https://glos24.pl/zakopane-znow-spor-o-kolejke
49	https://gory.pracownia.org.pl/upload/filemanager/gory.pracownia.org.pl/Dokumenty/Tatry_PNRWI_TPN_stanowisko_Kasprowy_360_15.05.2017.pdf
50	https://gospodarka.dziennik.pl/news/artykuly/581759,kolejka-na-kasprowy-wierch-tpn-pkl.html
51	https://info.wiara.pl/doc/3053933.TPN-Podswietlanie-krzyza-na-Giewoncie-nielegalne
52	https://innpoland.pl/153177,spor-pkl-z-tpn-nie-bedzie-inwestycji-bo-wladze-parku-wypowiedzialy-umowe
53	https://krakow.gosc.pl/doc/4371750.Boj-o-schroniska
54	https://krakow.tvp.pl/34369932/podroznik-marek-kaminski-wyruszyl-dookola-tatr
55	https://krakow.tvp.pl/34994775/tpn-i-pttk-tocza-spor-o-tatrzanskie-schroniska
56	https://krakow.wyborcza.pl/krakow/7,44425,22962678,kolejke-na-kasprowy-moze-przejac-tpn-analiza.html?disableRedirects=true
57	https://krakow.wyborcza.pl/krakow/7,44425,26479126,tatry-pttk-zachowa-grunty-pod-schroniskami-tpn-zyska-ponad.html
58	https://kultura.wiara.pl/doc/1485750.Drewniany-konflikt
59	https://kw.warszawa.pl/file/original/jptxsnsdrtbh/
60	https://kw.warszawa.pl/forum?ftype=topic&fekey=tpbscfmnnhwg&gpage=37
61	https://lovekrakow.pl/aktualnosci/wojna-o-turystow-na-kasprowym-wierchu_20417.html
62	https://m.podhale24.pl/aktualnosci/artykul/50233
63	https://m.podhale24.pl/aktualnosci/artykul/55400
64	https://markowepodroze.blogspot.com/2017/12/wyprawa-nad-morskie-oko-tatry-polska.html
65	https://napieraj.pl/szymon-ziobrowski-musimy-bronic-przyrody-zebyscie-mieli-gdzie-biegac/
66	https://nettg.pl/gornictwo/171908/pttk-zachowa-wszystkie-grunty-pod-schroniskami-a-zrzeknie-sie-wspolwlasnosci-ponad-tysiaca-hektarow-terenu-w-tatrach
67	https://next.gazeta.pl/next/7,151003,23656519,tatrzanski-park-narodowy-wzywa-do-demontazu-kolejki-na-kasprowy.html
68	https://nieruchomosci.dziennik.pl/news/artykuly/7995572,spor-pttk-tpn-historyczny-dzien-nieruchomosci-grunty-ziemia-schronisko.html
69	https://nietylko.design/010-joanna-rutkowska-badanie-uslug-w-tpn/
70	https://noizz.pl/big-stories/konie-nad-morskim-okiem-nadal-zameczane-sa-na-smierc-pojawi-sie-woz-hybrydowy/tqpyqnl
71	https://nowytarg.naszemiasto.pl/zakopianski-spor-o-drogi-solic-czy-sypac-zwirem/ar/c4-1739329
72	https://pch24.pl/spor-o-krzyz-na-rysach/
73	https://pfrsa.pl/aktualnosci/polski-fundusz-rozwoju-przejmuje-polskie-koleje-linowe.html

74	https://podhale24.pl/aktualnosci/artykul/24181/Bedzie_narodowa_skladka_na_zakup_kolejki_na_Kasprowy_Wierch.html
75	https://poland.us/strona,25,13250,0,czy-polacy-wykupia-kolejke-na-kasprowy.html
76	https://portaltatrzanski.pl/aktualnosci/tpn-i-pttk-dogadaly-sie-w-sprawie-gruntow-po-niespelna-30-latach,3708
77	https://rcin.org.pl/Content/79383/KR038_97845_r2007_IntegOP-Adamski-27-28.pdf
78	https://rebus.us.edu.pl/bitstream/20.500.12128/3377/1/Hibszer_Konflikty_czlowiek_przyroda_w_polskich_parkach.pdf
79	https://rodzina.wiara.pl/doc/4386189.Boj-o-schroniska
80	https://ruj.uj.edu.pl/xmlui/bitstream/handle/item/60748/bukowski_erozja_spoleczno-kulturowego_zwiazku_wspolnoty_etnicznej_2010.odt?sequence=2&isAllowed=y
81	https://ruj.uj.edu.pl/xmlui/handle/item/217127
82	https://serwisy.gazetaprawna.pl/transport/artykuly/927657,kolejna-na-kasprowy-gubalowke-zakopane-transport.html
83	https://sip.lex.pl/orzeczenia-i-pisma-urzedowe/orzeczenia-sadow/ii-sa-kr-1478-18-wyrok-wojewodzkiego-sadu-522728154
84	https://sportsinwinter.pl/czy-puchar-swiata-w-zakopanem-jest-zagrozony-wrocil-stary-spor/
85	https://tpn.pl/filebrowser/files/Wspieraj/tlumaczenie_wspolpraca_rmnp.pdf
86	https://tpn.pl/nowosci/swiatlo-na-giewoncie
87	https://tpn.pl/upload/filemanager/Ochrona%20%C5%9Bcis%C5%82a%20w%20parkach%20(2019)%20-%20e-book.pdf
88	https://tpn.pl/upload/filemanager/sekcja3_low-res.pdf
89	https://turysci.pl/tatry-041120-pk-wielki-historyczny-moment
90	https://turystyka.wp.pl/spor-o-nowa-kolejke-na-kasprowy-wierch-6044395852366465a
91	https://turystyka.wp.pl/tatry-koniec-wieloletniego-sporu-o-grunty-historyczny-moment-6572322801191616a
92	https://tvn24.pl/biznes/z-kraju/koniec-sporu-pttk-i-tatrzanskiego-parku-narodowego-o-grunty-w-tatrach-podpisano-porozumienie-4741243
93	https://tvn24.pl/polska/tatrzanski-park-narodowy-spiera-sie-o-grunty-pod-schroniskami-pttk-ra795915-2575611
94	https://vetpol.org.pl/dmdocuments/ZW-lipiec-2019.pdf
95	https://wgsr.uw.edu.pl/wgsr/wp-content/uploads/2018/11/GorczycaPIS41.pdf
96	https://wiadomosci.gazeta.pl/tatrzanski-park-narodowy
97	https://wiadomosci.onet.pl/krakow/zakopane-trwa-spor-o-kolejke-na-kasprowy-wierch-posiedzenie-w-styczniu/fdb89ge
98	https://wiadomosci.radiozet.pl/Polska/Ministerstwo-Srodowiska-wlacza-sie-w-spor-o-kolejke-na-Kasprowy-Wierch
99	https://wmeritum.pl/kolejka-na-kasprowy-wierch-zostanie-zdemontowana-tatrzanski-park-narodowy-pozywa-polskie-koleje-linowe/239651
100	https://wspinka.org/sites/default/files/pza-tpn-2009-m.pdf
101	https://wste.edu.pl/kronika/911-badania-czasowo-przestrzennego-ruchu-turystycznego-w-rejonie-kasprowego-wierchu/

102	https://wszechnicapolska.edu.pl/dokumenty/wydawnictwo/2011-J-Radziejowski-Obszary-chronionej-przyrody.pdf
103	https://www.bankier.pl/wiadomosc/PTTK-zachowa-grunty-pod-schroniskami-TPN-zyska-ponad-1-tys-ha-gruntow-7994337.html
104	https://www.bdpn.pl/dokumenty/roczniki/rb25/panel10.pdf
105	https://www.biznesinfo.pl/tatry-051120-kb-koniec-sporu
106	https://www.dbc.wroc.pl/Content/24715
107	https://www.dziennik.pl/tagi/tpn
108	https://www.fakt.pl/pieniadze/finanse/tatrzanski-park-narodowy-pozywa-kolejke-linowa/hhtwh1l
109	https://www.festiwalbiegowy.pl/biegajacy-swiat/sylwester-pod-wielka-krokwia-biegacze-gorscy-martwia-sie-o-przyrode-aktaualizacja#.Ydciw1Xdg2w
110	https://www.fly4free.pl/kolejka-kasprowy-wierch-zakopane/
111	https://www.forumkolejowe.pl/showthread.php?tid=6354
112	https://www.goryonline.com/tatrzanski-skituring---rob-to-bezpiecznie-i-z-glowa-,2005096,i.html
113	https://www.gov.pl/attachment/eb43767d-d5c5-4c9c-b205-2243e15fad80
114	https://www.kp.org.pl/pl/informacje/2448-400-ha-ochrony-biernej-mniej-w-lasach-tatrzanskiego-pn-interesy-gorali-wazniejsze-od-przyrody
115	https://www.miesiecznik.znak.com.pl/6732011andrzej-bukowskidemokracja-lokalna-a-dobro-wspolne-uwagi-sceptyka-2/
116	https://www.money.pl/archiwum/wiadomosci_agencyjne/pap/artykul/zakopane;ekolodzy;nie;chca;kolejki;na;kasprowy,164,0,307108.html
117	https://www.money.pl/gospodarka/wiadomosci/artykul/kasprowy-wierch-kolejka,131,0,2416771.html
118	https://www.money.pl/gospodarka/wiadomosci/artykul/kolejka-na-kasprowy-pfr-polskie-koleje-linowe,242,0,2418162.html
119	https://www.narty.pl/tresc-artykulu/konflikt-pkl-z-tpn-o-kolej-na-kasprowy-wierch-glos-pkl
120	https://www.naszkasprowy.pl/prywatyzacja-pkl,76,Obawiam_sie_agresji_ze_strony_nowego_wlasciciela.html
121	https://www.newsweek.pl/marta-fogler-o-tym-co-przeszkadza-w-rozwoju-infrastruktury-tatr/6q0h6kt
122	https://www.newsweek.pl/polska/spoleczenstwo/tatrzanski-pn-zada-rozbiorki-kolejki-na-kasprowy/2xgmc9j
123	https://www.newsweek.pl/polska/tatrzanski-park-narodowy-wozy-wozace-turystow-do-morskiego-oka/xx3qv4h
124	https://www.niedziela.pl/artykul/139663/nd/Kolejka-na-Kasprowy-Wierch-wrocila-do
125	https://www.onet.pl/#e404
126	https://www.onet.pl/#e404
127	https://www.pap.pl/aktualnosci/news%2C749789%2Ctatry-pttk-zachowa-grunty-pod-schroniskami-park-narodowy-zyska-ponad-1-tys
128	https://www.polsatnews.pl/wiadomosc/2018-03-15/spor-o-kolejke-na-kasprowy-wierch-rozmowy-tatrzanskiego-pn-z-pkl-zakonczone-fiaskiem/
129	https://www.polsatnews.pl/wideo/polskie-koleje-linowe-nie-zatrzymaja-kolejki-na-kasprowy-i-nie-zaplaca-dzierzawy-tatrzanskiemu-parkowi-narodowemu-spor-trwa_6570123/
130	https://www.polskieradio.pl/7/129/Artykul/933305,Spor-o-konie-spor-o-logo

131	https://www.powiat.tatry.pl/index.php/news/samorzad/716-przelomowe-porozumienie-dotyczace-polskiej-czesci-tatr
132	https://www.przegladsportowy.pl/sporty-zimowe/jak-w-zakopanem-zaniedbano-sporty-zimowe/f9hcvx1
133	https://www.przyrodnicze.org/puszcza/ludzie-mogliby-zyc-spokojnie-w-krainie-niedzwiedzi
134	https://www.radiokrakow.pl/aktualnosci/zakopane/co-dalej-z-kolejka-na-kasprowyjest-odpowiedz-tpn/
135	https://www.radiokrakow.pl/aktualnosci/zakopane/spor-tpn-z-pkl-nie-trafi-na-wokande/
136	https://www.radiokrakow.pl/spor-o-pkl/
137	https://www.radiomaryja.pl/informacje/spor-o-kolejke-na-kasprowy-wierch/
138	https://www.ratujkonie.pl/konie-z-morskiego-oka/ratujmy_konie_z_morskiego_oka/
139	https://www.rdn.pl/news/tatrzanski-park-narodowy-pkl-spotkaly-sie-sadzie-sprawie-kolejki-kasprowy-wierch
140	https://www.researchgate.net/profile/Bartlomiej-Walas/publication/331385253_MODEL_OPTYMALIZACJI_FUNKCJONOWANIA_PARKOW_NARODOWYCH_W_POLSCE_W_OTOCZENIU_SPOLECZNO-GOSPODARCZYM_WYZSZA_SZKOLA_TURYSTYKI_I_EKOLOGII_-SUCHA_BESKIDZKA/links/5c76da8c299bf1268d2afe22/MODEL-OPTYMALIZACJI-FUNKCJONOWANIA-PARKOW-NARODOWYCH-W-POLSCE-W-OTOCZENIU-SPOLECZNO-GOSPODARCZYM-WYZSZA-SZKOLA-TURYSTYKI-I-EKOLOGII-SUCHA-BESKIDZKA.pdf
141	https://www.researchgate.net/publication/284030409_Metody_zapobiegania_nielegalnej_dyspersji_turystow_i_zwiazanej_z_nia_erozji_gleby_w_Tatrzanskim_Parku_Narodowym_Methods_for_prevention_of_illegal_touristic_dispersion_related_to_soil_erosion_in_the_Ta
142	https://www.rmf24.pl/fakty/polska/news-jak-uniknac-ataku-niedzwiedzia,nId,1036254#crp_state=1
143	https://www.rmf24.pl/news-konflikt-tpn-i-cos-czy-zagrozi-pucharowi-swiata-w-zakopanem,nId,3096856#crp_state=1
144	https://www.rp.pl/spoleczenstwo/art1043461-tatrzanski-park-narodowy-przeciw-imprezie-tvp-pod-wielka-krokwia-zle-dla-przyrody
145	https://www.salon24.pl/u/magazyn/1002140,sylwester-2019-w-zakopanem
146	https://www.schronisko.net/art/aktualnosci/zakonczenie-sporu-tpn---pttk/bkbwa921
147	https://www.sejm.gov.pl/sejm7.nsf/biuletyn.xsp?documentId=BCD3612228BE1FECC1257DFC00506045
148	https://www.skokinarciarskie.pl/aktualnosci/14304,Tajner_o_konflikcie_z_COS_PZN_chce_przejac_Wielka_Krokiew
149	https://www.skyscrapercity.com/threads/ma%C5%82opolska-inwestycje-wydarzenia-wizje.287961/page-31
150	https://www.skyscrapercity.com/threads/zakopane-elektryczna-komunikacja-miejska.670676/
151	https://www.sport.pl/skoki/7,65074,25000728,puchar-swiata-w-zakopanem-jest-zagrozony-powrocil-stary-spor.html
152	https://www.tatry-przewodnik.com.pl/blog/?ssaki-w-tatrach
153	https://www.tokfm.pl/Tokfm/7,103085,25384878,tpn-o-sylwestrze-kurskiego-pod-wielka-krokwia-nikt-nas-nie.html
154	https://www.transport-publiczny.pl/mobile/transport-nad-morskie-oko-czy-w-ogole-potrzebny-46489.html
155	https://www.tvp.info/35814679/spor-o-kolejke-na-kasprowy-tatrzanski-park-narodowy-chce-ugody
156	https://www.tygodnikpowszechny.pl/tatry-coraz-bardziej-wspolne-17520

157	https://www.wnp.pl/logistyka/pkl-i-tpn-prowadza-spor-o-dzierzawe-znamy-rezultaty-rozmow,319603.html
158	https://www.wprost.pl/415845/tatrzanski-park-narodowy-idzie-do-sadu.html
159	https://www.wwf.pl/sites/default/files/2017-07/Ochrona%20gatunkowa%20rysia%2C%20wilka%20i%20nied%C5%BAwiedzia%20w%20Polsce%2C%20raport%20ko%C5%84cowy%202012_0.pdf
160	https://www.wydawnictwo.wsb.pl/sites/wydawnictwo.wsb.pl/files/czasopisma-tresc/07_Kasprowiak.pdf
161	https://www.zrodla.org/pdf/raport-podreczniki.pdf
162	https://wydarzenia.interia.pl/kraj/news-spor-o-tatrzanskie-schroniska-fiasko-negocjacji,nId,2470016
163	https://zakopane.naszemiasto.pl/afera-w-tatrzanskim-parku-narodowym-instytucja-wydala-z/ar/c1-4381527
164	https://zakopane.naszemiasto.pl/tatry-tpn-wreszcie-stal-sie-formalnym-wlascicielem-giewontu/ar/c1-7981457
165	https://z-ne.pl/s,doc,24474,1,1532,wraca_spor_o_orla_perc.html

References

Abdi, H. & Williams, L.J. Principal component analysis. *Wiley Interdiscip. Rev.-Comput. Stat.* **2**, 433-459 (2010).

Affek, A. Propozycje wskaźników środowiskowych do oceny zagospodarowania przestrzennego i ładu przestrzennego w gminach. *Biuletyn KPZK* **252**, 51-86 (2013).

Barczyk-Ciuła, J., M. Nogieć, W. Sroka & Wojewodzic, T. Pozarolnicza działalność gospodarcza w gminach położonych w zasięgu oddziaływania Krakowskiego Obszaru Metropolitalnego. *Roczniki Naukowe Ekonomii Rolnictwa i Rozwoju Obszarów Wiejskich* **105**, 47-57 (2018).

Bernat, S. Analysis of Social Conflicts in Poland's Soundscape as a Challenge to Socio-Acoustics. *Archives of Acoustics* **41**, 415-426 (2016).

Biebrzański Park Narodowy. *Zestawienie powierzchni według kategorii użytkowania stan na 31.12.2019 r. zgodnie ze sprawozdaniem GUS.* (2019). at <https://www.bip.biebrza.org.pl/plik,4869,zestawienie-powierzchni-wedlug-kategorii-uzytkowania-stan-na-31-12-2019-r-zgodnie-ze-sprawozdaniem-gus-pdf.pdf>

Bielecka, M. & Różyński, G. Management conflicts in the Vistula Lagoon area. *Ocean & Coastal Management* **101**, 24-34 (2014).

Binda, A. *Finanse gmin województwa małopolskiego w obliczu pandemii COVID-19*. (Urząd Marszałkowski Województwa Małopolskiego, 2020).

Borkowski, J. *et al.* High density of keystone herbivore vs. conservation of natural resources: Factors affecting red deer distribution and impact on vegetation in Słowiński National Park, Poland. *Forest Ecology and Management* **450**, 117503 (2019).

Brown, G. *et al.* Cross-cultural values and management preferences in protected areas of Norway and Poland. *Journal for Nature Conservation* **28**, 89-104 (2015).

Bucała-Hrabia, A. Land use changes and their catchment-scale environmental impact in the Polish Western Carpathians during transition from centrally planned to free-market economics. *Geographia Polonica* **91**, 171-196 (2018).

Cash, D. W. *et al.* Scale and Cross-Scale Dynamics: Governance and Information in a Multilevel World. *Ecology and Society* **11**, 8 (2006).

Chmielewski, T. J., Śleszyński, P., Chmielewski, S. & Kułak, A. *Ekologiczne i fizjonomiczne koszty bezładu przestrzennego*. (Instytut Geografii i Przestrzennego Zagospodarowania im. Stanisława Leszczyckiego PAN, Uniwersytet Przyrodniczy w Lublinie, Komitet Przestrzennego Zagospodarowania Kraju przy Prezydium PAN, 2018).

Chmielewski, W. & Głogowska, M. Implementation of the Natura 2000 Network in Poland – an Opportunity or a Treat to Sustainable Development of Rural Areas? Study on Local Stakeholders' Perception. *Eastern European Countryside* **21**, 153-169 (2015).

Chrzanowska, M. & Drejerska, N. Ocena rozwoju społeczno-gospodarczego gmin województwa mazowieckiego z wykorzystaniem metod analizy wielowymiarowej. *Wiadomości Statystyczne* **6,** 59-69 (2016).

Statistics, Poland. The NUTS classification in Poland (2022). at <https://stat.gov.pl/en/regional-statistics/classification-of-territorial-units/classification-of-territorial-units-for-statistics-nuts/the-nuts-classification-in-poland/>

Dominiak, J. & Konecka-Szydłowska, B. Kapitał społeczny w aglomeracji poznańskiej – zmiany w układzie rdzeń–peryferie. *Rozwój Regionalny i Polityka Regionalna* **50,** 101-121 (2020).

Działek, J. *Kapitał społeczny jako czynnik rozwoju gospodarczego w skali regionalnej i lokalnej w Polsce.* (Wydawnictwo Uniwersytetu Jagiellońskiego, 2011).

Dziekański, P. Przestrzenne zróżnicowanie infrastruktury gmin województwa świętokrzyskiego. *Zeszyty Naukowe Polskiego Towarzystwa Ekonomicznego w Zielonej Górze* **3**, 92-101 (2016).

European Commission. *The Natura 2000 Biogeographical regions* (2022). at <https://ec.europa.eu/environment/nature/natura2000/biogeog_regions/>

Generalna Dyrekcja Ochrony Środowiska. *Centralny Rejestr Form Ochrony Przyrody.* (2022). at <https://crfop.gdos.gov.pl/CRFOP/>

Gonia, A. & Podgórski, Z. Ocena stanu rozwoju funkcji turystycznej gmin województwa kujawsko-pomorskiego. *Czasopismo Geograficzne* **90**, 88-104 (2019).

Grodzinska-Jurczak, M. & Cent, J. Expansion of Nature Conservation Areas: Problems with Natura 2000 Implementation in Poland? *Environmental Management* **47**, 11-27 (2011).

Hajduk, S. Instrumenty zarządzania przestrzennego w aspekcie zrównoważonego rozwoju–wielowymiarowa analiza porównawcza miast wojewódzkich. *Rocznik Ochrona Środowiska* **20**, 1219-1233 (2018).

Hok, B. Ocena zamożności gmin na przykładzie wybranych gmin województwa zachodniopomorskiego. *Ekonomiczne Problemy Usług* **118**, 123-136 (2015).

Jankowiak, Ł. *et al.* Patterns of occurrence and abundance of roosting geese: the role of spatial scale for site selection and consequences for conservation. *Ecological Research* **30**, 833-842 (2015).

Janus, J. & Taszakowski, J. Propozycja wskaźnika określającego stopień rozdrobnienia gruntów oraz jego zastosowanie na przykładzie powiatu Dąbrowskiego. *Infrastruktura i Ekologia Terenów Wiejskich* **2**, 75–85 (2013).

Jolliffe, I.T. & Cadima, J. Principal component analysis: a review and recent developments. *Philos. Trans. R. Soc. A* **374**, 20150202 (2016).

Józefowicz, I. & Michniewicz-Ankiersztajn, H. Rozwój społeczno-gospodarczy a aktywność społeczności lokalnych w województwie wielkopolskim. *Rozwój Regionalny i Polityka Regionalna* **52** 29-42 (2020).

Kałuża, H. & Kałuża, J. Wpływ infrastruktury na rozwój przedsiębiorczości na przykładzie gmin powiatu płońskiego. *Studia i Prace WNEiZ* **47**, 117-125 (2017).

Kędzierska, M., Łuczak, M. J., Mazur-Łuczak, J., Nowosielska, A. & Nowosielski, M. *Społeczno-ekonomiczne wymiary ubóstwa w perspektywie różnic terytorialnych w Wielkopolsce. Raport z badania*. (Obserwatorium Integracji Społecznej, 2013).

Kieniewicz, S. *The emancipation of the Polish peasantry*. (University of Chicago Press, 1969).

Kołodziejczyk, D. Infrastruktura w rozwoju społeczno-gospodarczym gmin w Polsce. *Prace Naukowe Uniwersytetu Ekonomicznego we Wrocławiu* **360**, 198-206 (2014).

Kot-Niewiadomska, A. & Pawłowska, A. The Possibilities of Open-Cast Mining in Landscape Parks in Poland—A Case Study. *Resources* **9**, 122 (2020).

Kozera, A., Głowicka-Wołoszyn, R. & Wysocki, F. Samodzielność finansowa gmin wiejskich województwa wielkopolskiego w latach 2005-2013. *Stowarzyszenie Ekonomistów Rolnictwa i Agrobiznesu. Roczniki Naukowe* **XVII**, 198-204 (2015).

Kubacka, M. & Macias, A. The Functioning of Natura 2000 Areas in the Opinion of Different Groups From the Local Community: A Case Study From Poland. *Society & Natural Resources* **29**, 1186-1197 (2016).

Kubacka, M. The Role of Local Association of Communes in Environmental Management Systems: Selected Case Studies in the Wielkopolska Region. *Polish Journal of Environmental. Studies* **21**, 1287-1293 (2012).

Kubo, T & Shoji, Y. Demand for bear viewing hikes: Implications for balancing visitor satisfaction with safety in protected areas. *Journal of Outdoor Recreation and Tourism* **16**, 44-49 (2016).

Statistics, Poland. *Local Data Bank*. (2021). at <https://bdl.stat.gov.pl/BDL/start>

Loc, H.H., Park, E., Thu, T.N., Diep, N.T.H. & Can, N.T. An enhanced analytical framework of participatory GIS for ecosystem services assessment applied to a Ramsar wetland site in the Vietnam Mekong Delta. *Ecosyst. Serv.* **48**, 10124 (2021).

Logmani, J., Krott, M., Lecyk, M. T. & Giessen, L. Customizing elements of the International Forest Regime Complex in Poland? Non-implementation of a National Forest Programme and redefined transposition of NATURA 2000 in Bialowieza Forest. *Forest Policy and Economics* **74**, 81-90 (2017).

Lorencowicz, E., Mazurek, K. & Kocira, S. Próba aktualizacji metody określania intensywności organizacji rolnictwa. *Roczniki Naukowe Stowarzyszenia Ekonomistów Rolnictwa i Agrobiznesu* **XIX**, 92-98 (2017).

Maczka, K., Chmielewski, P., Jeran, A., Matczak, P. & van Riper, C. J. The ecosystem services concept as a tool for public participation in management of Poland's Natura 2000 network. *Ecosystem Services* **35**, 173-183 (2019).

Majchrzak, A. Zróżnicowanie rozwoju rolnictwa województwa wielkopolskiego w przekroju gmin. *Acta Scientarium Polonorum Oeconomica* **7**, 55-67 (2008).

Malinowski, M., Krakowiak-Bal, A., Sikora, J. & Woźniak, A. Ilości generowanych odpadów komunalnych w aspekcie typów gospodarczych gmin województwa małopolskiego. *Infrastruktura i Ekologia Terenów Wiejskich* **9**, 181-191 (2009).

Męczekalski, M., Dubownik, A. & Rudnicki, R. Rozwój agroturystyki w województwie kujawsko-pomorskim jako efekt absorpcji środków unijnych przeznaczonych na różnicowanie działalności gospodarczej na obszarach wiejskich. *Roczniki Naukowe Wyższej Szkoły Wychowania Fizycznego i Turystyki w Białymstoku* **4**, 51-60 (2017).

Michalska-Żyła, A. Jakość życia na poziomie lokalnym. *Acta Universitatis Lodziensis. Folia Sociologica* **56,** 53-66 (2016).

Miedziński, M. Niedoskonałości GUS w zakresie turystyki na przykładzie bazy noclegowej polskich powiatów nadmorskich. in: *Badania nad turystyką. Jeden cel, różne podejścia* (eds. Makowska-Iskierka, M.), 101-116. (Wyd. Uniwersytetu Łódzkiego, 2015).

Miłek, D. Ocena jakości życia w gminach regionu świętokrzyskiego. *Studia Prawno-Ekonomiczne* **107**, 305–320 (2018).

Nature Conservation Act (2004).

Niedziałkowski, K., Paavola J. & Jędrzejewska, B. Governance of biodiversity in Poland before and after the accession to the EU: the tale of two roads. *Environmental Conservation* **40**, 108-118 (2012a).

Niedziałkowski, K., Paavola J. & Jędrzejewska, B. Participation and Protected Areas Governance: the Impact of Changing Influence of Local Authorities on the Conservation of the Białowieża Primeval Forest, Poland. *Ecology and Society* **17,** 2 (2012b).

Niedziałkowski, K., Blicharska, M., Mikusiński, G. & Jędrzejewska, B. Why is it difficult to enlarge a protected area? Ecosystem services perspective on the conflict around the extension of the Białowieża National Park in Poland. *Land Use Policy* **38**, 314-329 (2014).

Ociepa-Kicińska, E. Rozwój społeczno-gospodarczy gmin Szczecińskiego Obszaru Metropolitalnego na tle województwa zachodniopomorskiego. *Rozwój Regionalny i Polityka Regionalna* **47**, 111-123 (2019).

Olko, J., Hędrzak, M., Cent, J. & Subel, A. Cooperation in the Polish national parks and their neighborhood in a view of different stakeholders – a long way ahead*? Innovation: The European Journal of Social Science Research* **24**, 295-312 (2011).

Paloniemi, R. *et al.* Public Participation and Environmental Justice in Biodiversity Governance in Finland, Greece, Poland and the UK. *Environmental Policy and Governance* **25**, 330-342 (2015).

Pałka, E. Diagnoza bazy agroturystycznej w województwie świętokrzyskim w aspekcie procesów integracyjnych. *Infrastruktura i Ekologia Terenów Wiejskich* **1**, 149–161 (2007).

Piwowarczyk, J. & Wróbel, B. Determinants of legitimate governance of marine Natura 2000 sites in a post-transition European Union country: A case study of Puck Bay, Poland. *Marine Policy* **71**, 310-317 (2016).

Plit, J. Regionalizacja współczesnych krajobrazów historyczno-kulturowych Polski. *Prace Komisji Krajobrazu Kulturowego* **27**, 79-94 (2015).

Regionalna Dyrekcja Ochrony Środowiska w Krakowie. *Formy ochrony przyrody*. (2022). at <https://www.gov.pl/web/rdos-krakow/formy-ochrony-przyrody>

Rechciński, M., Grodzińska-Jurczak, M & Tusznio, J. Konflikty wokół polskich obszarów chronionych w artykułach naukowych o międzynarodowym zasięgu. Ilościowy systematyczny przegląd literatury. *Przegląd Przyrodniczy* **XXIX**, 50-72 (2018).

Rosner, A. & Stanny, M. *Socio-economic development of rural areas in Poland*. (The European Fund for the Development of Polish Villages Foundation, Institute of Rural and Agricultural Development, Polish Academy of Sciences, 2017).

Salama, M.A., Hassanien, A.E., & Fahmy A.A. Deep Belief Network for clustering and classification of a continuous data. in: *The 10th IEEE International Symposium on Signal Processing and Information Technology*, 473-477 (IEEE, 2010).

Sejm Committees' Bureau. *Full record of the meeting of the Commission of The Environmental Protection, Natural Resources and Forestry on 14 December 2020* (Chancellery of the Sejm, 2020).

Smętkowski, M., Jałowiecki, B. & Gorzelak, G. *Obszary metropolitalne w Polsce : Problemy rozwojowe i delimitacja* (Centrum Europejskich Studiów Regionalnych i Lokalnych EUROREG, Uniwersytet Warszawski, 2009).

Solon, J. *et al.* Physico-geographical mesoregions of Poland: Verification and adjustment of boundaries on the basis of contemporary spatial data. *Geographia Polonica* **91**, 143-170 (2018).

Sołtys, J. & Dorocki, S. Wskaźnik przedsiębiorczości w jednostkach terytorialnych Polski – zróżnicowanie w czasie i przestrzeni. *Przedsiębiorczość - Edukacja* **12**, 18-35 (2016).

Sroka, W. *Zmiany struktur agrarnych w województwie małopolskim – rozwój czy stagnacja?* (Towarzystwo Ekonomistów Polskich, 2018).

Strzelecka, M., Tusznio, J., Rechcinski, M., Bockowski, M. & Grodzinska-Jurczak, M. Resident Perceptions of Distribution, Recognition and Representation Justice Domains of Environmental Policy-Making: The Case of European Ecological Network Natura 2000 in Poland. *Society & Natural Resources* **34**, 248-268 (2020).

Synówka-Bejenka, E. Nowa propozycja wskaźnika rozwoju funkcji turystycznej. *Studia Ekonomiczne. Zeszyty Naukowe Uniwersytetu Ekonomicznego w Katowicach* **335**, 64-76 (2017).

Szmytkie, R. & Tomczak, P. Kierunki rozwoju obszarów wiejskich województwa dolnośląskiego po akcesji do Unii Europejskiej. *Studia KPZK* **167**, 398-417 (2016).

Śleszyński, P. Zmiany strukturalne i przestrzenno-funkcjonalne w rozwoju przedsiębiorczości po przystąpieniu Polski do Unii Europejskiej. *Studia Regionalne i Lokalne* **37**, 5-26 (2009).

Śleszyński, P., Deręgowska, A., Kubiak, Ł., Sudra, P. & Zielińska, B. *Analiza stanu i uwarunkowań prac planistycznych w gminach w 2017 roku* (Instytut Geografii i Przestrzennego Zagospodarowania PAN na zlecenie Ministerstwa Inwestycji i Rozwoju, 2018).

Śleszyński, P., Gibas, P. & Sudra, P. The Problem of Mismatch between the CORINE Land Cover Data Classification and the Development of Settlement in Poland. *Remote Sensing* **12**, 2253 (2020).

Śmietanka, T. Konkurencyjność gminy Kozienice w ujęciu podregionalnym i lokalnym (na podstawie GUS 2010-2016 i SWOT 2018). *Prace Naukowe Uniwersytetu Ekonomicznego we Wrocławiu* **527**, 264-299 (2018).

UNEP-WCMC and IUCN. *Protected Planet: The World Database on Protected Areas (WDPA) and World Database on Other Effective Area-based Conservation Measures (WD-OECM)*. (UNEP-WCMC and IUCN, 2022).

Warczewska, B. Rozwój infrastruktury turystycznej na przykładzie dolnośląskich parków krajobrazowych. *Prace Naukowe Uniwersytetu Ekonomicznego we Wrocławiu* **473**, 579-586 (2017).

Wasilewski, M., Szulczewska, B. & Giedych, R. Visitors' Perception of Urban Nature Reserves in Poland. *Sustainability* **11**, 3768 (2019).

Wiątkowski, M., Rosik-Dulewska, C. & Tomczyk, P. Hydropower Structures in the Natura 2000 Site on the River Radew: an Analysis in the Context of Sustainable Water Management. *Rocznik Ochrona Środowiska* **19**, 65-80 (2017).

Wilkaniec, A., Gałecka-Drozda, A., Raszeja, E. & Szczepańska, M. Urbanisation Processes in Puszcza Zielonka Landscape Park in Poland - and Its Buffer Zone in the Context of Protection of Natural Structures. *Applied Ecology and Environmental Research* **18**, 697-712 (2020).

Wojewódzka-Wiewiórska, A. Strukturalny wymiar kapitału społecznego w Polsce. *Prace Naukowe Uniwersytetu Ekonomicznego we Wrocławiu* **347,** 522-532 (2014).

Yim, O. & Ramdeen, K.T. Hierarchical Cluster Analysis: Comparison of Three Linkage Measures and Application to Psychological Data. *The Quantitative Methods for Psychology* **11**, 8-21 (2015).

Yong, A.G. & Pearce, S. A Beginner's Guide to Factor Analysis: Focusing on Exploratory Factor Analysis. *Tutor. Quantit. Method. Psychol.* **9**, 79-94 (2013).

Zawadka, J. Metoda oceny poziomu rozwoju agroturystyki w jednostkach samorządu terytorialnego Stowarzyszenie Ekonomistów Rolnictwa i Agrobiznesu. *Stowarzyszenie Ekonomistów Rolnictwa i Agrobiznesu. Roczniki Naukowe* **XV**, 235-239 (2013).

Zawilińska, B. Residents' Attitudes Towards a National Park Under Conditions of Suburbanisation and Tourism Pressure: A Case Study of Ojców National Park (Poland). *European Countryside* **12**, 119-137 (2020).
